# Intramolecular Stabilization of Naphtho[2,1‑*b*:3,4‑*b*′]dithiophenes: Synthesis and Analysis of Imine-Based Chromophores

**DOI:** 10.1021/acs.joc.5c02486

**Published:** 2025-12-16

**Authors:** Emmanuel B. A. Adusei, Sarah Ibrahim, Kara Jenneker, Calvin D. Goldsmith, Danielle Dragoi, Matthias Zeller, Zacharias J. Kinney

**Affiliations:** a Department of Chemistry, 6918 Oakland University, Rochester, Michigan 48309, United States; b Department of Chemistry, 8522 Purdue University, West Lafayette, Indiana 47907, United States

## Abstract

The role of noncovalent interactions in stabilizing and organizing complex structures throughout nature is indisputable. Of the various classes of noncovalent interactions, those that involve secondary bonding – attractive interactions between σ-hole and nucleophile – are of interest in the design of materials due to their strength and programmability. This report takes the approach of placing nucleophilic imines in close proximity to fused thiophene moieties within naphtho­[2,1-*b*:3,4-*b*′]­dithiophene (α NDT) cores, where an intramolecular N···S interaction is poised to yield rigid chromophores. These types of intramolecular N···S interactions have been observed in the solid-state for several decades, but their solution-state analysis remains rare. Here we detail how crystallography, ^1^H/^13^C NMR spectroscopy, and molecular modeling work synergistically to describe the strength and impact of intramolecular N···S interactions on α NDT chromophores **α­(1)_2_**. The remote substituents on the aryl amines (**1**) employed as condensation partners have minimal structural impact on the **α­(1)**_**2**_ series, but the photophysical properties of strongly electron-deficient (**1d** and **1dd**) or polarizing (**1c** and **1cc**) end-caps are enhanced in comparison to their neutral (**1a**) and weak (**1b** and **1bb**) counterparts. This design strategy to incorporate intramolecular N···S interactions highlights how NDTs can be incorporated into complex architectures in a programmable manner.

## Graphical abstract

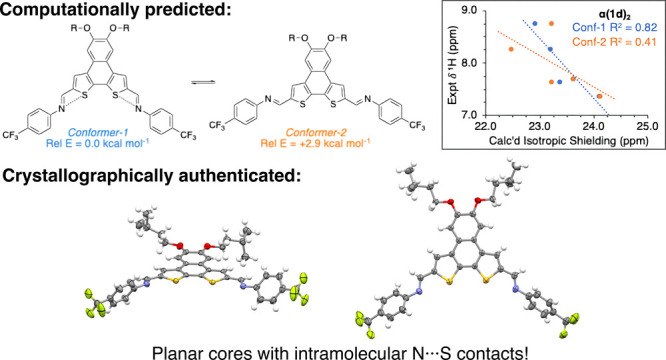

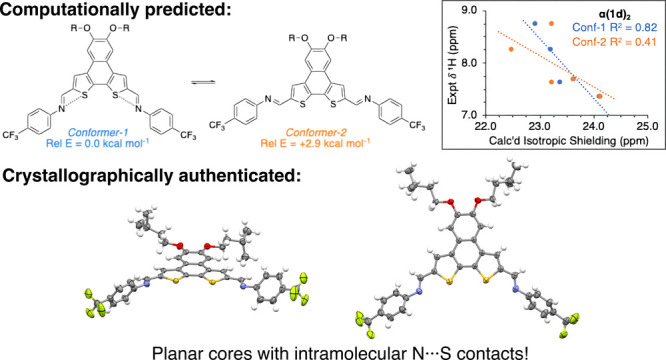

## Introduction

Heteroarenes containing thiophene moieties have been established as viable building blocks throughout organic electronics,^1,2^ with naphthodithiophenes^3^ finding applications in organic light-emitting diodes (OLEDs),^4^ organic field-effect transistors (OFETs),^5–7^ hole-transporting materials,^8,9^ and as a photoactive material within solar cells.^10–12^ As currently defined there are three isomeric classes of naphthodithiophenes: linear, angular, and bent.^3,13,14^ The bent NDTs are underrepresented as luminescent small molecules,^8,9,14^ presumably due to limited access to versatile building blocks that allow for late-stage derivatization^15,16^ and the generally poor emissive properties of thiophenes.^17^ Recently our laboratory has explored end-capping the isomeric bent NDTs – naphtho­[2,1-*b*:3,4-*b’*]­dithiophene (α) and naphtho­[1,2-*b*:4,3-*b’*]­dithiophene (β) – and probing their photophysical properties.^14^ The α series – fashioned with phenyl, *p*-tolyl, *p*-methoxyphenyl, and *p*-trifluoromethylphenyl end-caps – yielded photoactive molecules with strong defined emission (Φ ≈ 0.20–0.40, general depiction in Figure 1a). While insightful, this method of end-capping leaves little room for further derivatization or enhancement of their photoactive properties. Therefore, it is of interest to develop methods to access versatile α monomers that can be utilized in a variety of ways (i.e., late-stage derivatization of small molecules, functionalization postreaction, or incorporation into polymeric systems).

**Figure 1:**
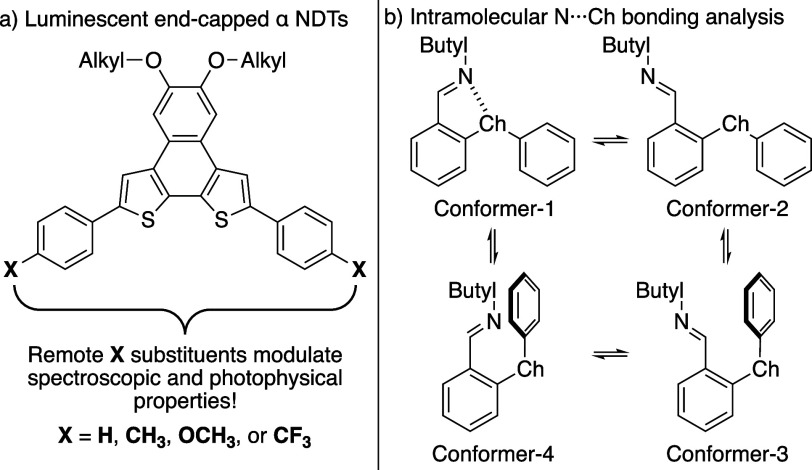
a) Luminescent α NDTs with phenylene-based end-caps; b) Work from You and Co-workers investigating intramolecular chalcogen (Ch) bonding involving imines.

In this regard imines^18^ offer several unique advantages: these dynamic linkages are predictable (i.e., the *E* conformation is thermodynamically more favorable than the *Z* conformation) and if placed adjacent to the thiophene cores are known to prefer the cisoid orientation; thus allowing the imine nitrogen to interact with the interior sulfur σ-hole.^19,20^ Figure 1b highlights work from You and co-workers elucidating the conformational landscape of chalcogen bonding^21,22^ between chalcogen bond donors (imines) and various chalcogen bond accepting chalcones (based on sulfur, selenium, and tellurium).^23^ Of the four conformers available the most populated configuration is conformer-1, wherein the imine nitrogen is aligned with the chalcogen σ-hole forming a five membered ring held together via an intramolecular N···S interaction. In light of this finding, we hypothesized that α NDTs equipped with imines could form intramolecular N···S contacts, yielding rigidified photoactive molecules.

Herein we describe the synthesis of α NDTs functionalized with a range of variable electronic amine-based end-caps. Due to the orientation of the imine units and the planar nature of the NDT core, there are two potential *E* conformers available (shown in Figure 2, top). The remote substituents on the aniline-derived end-caps (Figure 2, bottom) are vital in modulating the photophysical properties of the molecules, termed **α­(1)** **_2_**, and can influence the *E*/*Z* dynamics of the imine linkage itself. To observe how the remote substituents communicate with the NDT core a multi-tier approach has been taken that blends solution-state properties (NMR, UV–visible, and emission spectroscopy) with crystallographic and computational analysis. In total, the conformational landscape for the synthesized **α­(1)** **_2_** imine series is quite distinct, with a single orientation being preferred in both the solution- and solid-states.^24^

**Figure 2:**
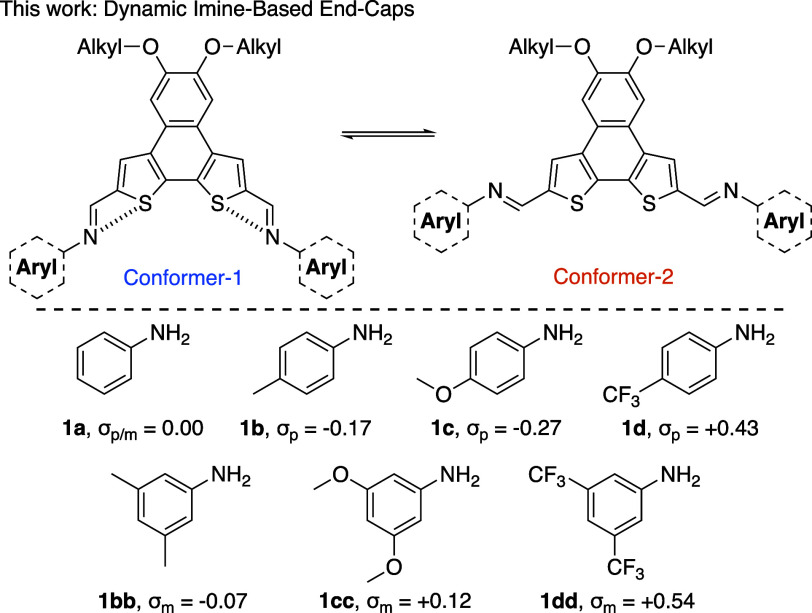
Top: general depiction of the two *E*-imine conformations available to α NDTs. Bottom: amines used in this study.

## Results and Discussion

### Synthesis of **α­(CHO)**_2_ and **α­(1)_2_** Series

Dialdehyde, **α­(CHO)** **_2_** shown in Scheme 1, can be reached in four steps from 4,5-dibromocatechol^25^ (four-step yield ∼ 10%, full details provided in the SI). Isopentyloxy groups were chosen to balance solution processability and crystallizability while minimizing solution-state aggregation.^26^ Due to the vast array of intramolecular N···S interactions observed in the solid-state for benzothienyl–aryl imines^27–35^ it was imperative to maintain crystallizability for the **α­(1)** **_2_** series. The synthetic path to solubilized **α** has been described in the literature (albeit with different solubilizing groups^14,36–38^ ), leaving only the addition of formyl groups to the 2,9-positions for optimization. Dialdehyde synthesis was achieved by probing two aldehyde sources – *N*,*N*-dimethylformamide (DMF) and 1-formylpiperidine – with dry DMF providing the best conversion of dilithiated **α** to **α­(CHO)** **_2_** (42% yield). With the newly installed *bis*-formyl groups at the 2,9-positions, **α­(CHO)** **_2_** is primed to undergo imine condensation with commercially available aniline derivatives (Figure 2, bottom): aniline (**1a**), 4-methylaniline (**1b**), 4-methoxyaniline (**1c**), 4-(trifluoromethyl)­aniline (**1d**), 3,5-dimethylaniline (**1bb**), 3,5-dimethoxyaniline (**1cc**), and 3,5-*bis*(trifluoromethyl)­aniline (**1dd**). This series of aniline derivatives have a range of electronic properties to modulate the strength of intramolecular N···S interactions or tune the photophysical properties of the di-imine targets.

**Scheme 1:**
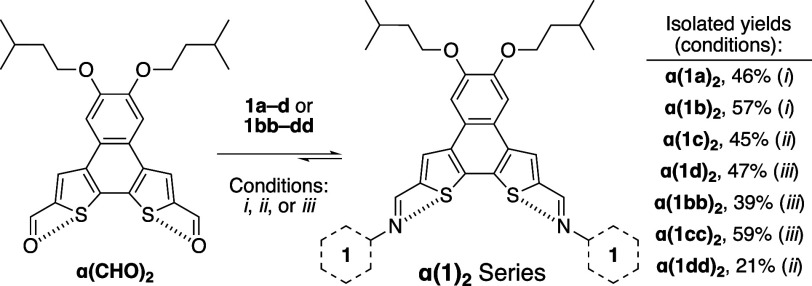
Synthesis of **α­(1)** **_2_** Series^a^

a Conditions: (*i*) TFA, CHCl_3_, 4 Å mol. sieves; (*ii*) Sc­(OTf)_3_, MeCN, CHCl_3_, 4 Å mol. sieves; (*iii*) toluene, AcOH, Δ.

One of the central benefits of imine condensations are the variety of well-established methodologies available to drive these dynamic reactions forward: (*i*) acid-catalyzed condensation in the presence of molecular sieves, (*ii*) scandium triflate-mediated condensation with molecular sieves,^39^ and (*iii*) thermal azeotropic removal of water.^18^ In general condition *ii* proved effective to access the target di-imines **α­(1)** **_2_** in modest yields (≈ 33% average for the series, see Table S1). The Sc­(OTf)_3_ conditions were particularly efficient at activating the electron deficient aryl amines (**1d** and **1dd**), which did not proceed to any appreciable extent with trifluoroacetic acid (TFA) as catalyst. For cases in which conditions *i* or *ii* yielded poor results (<15% isolated product) thermal conditions (conditions *iii*) were probed to ensure that the best methods were being applied. In the cases of **α­(1d)** **_2_**, **α­(1bb)** **_2_**, and **α­(1cc)** **_2_** the isolated yields for conditions *iii* were elevated to be in-line with the modest yields of the efficient coupling partners **α­(1a-c)** **_2_**. Due to the sensitivity of imines to hydrolysis the **α­(1)** **_2_** series was purified via gel permeation chromatography (GPC) using toluene as eluent. Representative chromatograms of **α­(1)** **_2_** illustrate conversion efficiency and the effectiveness of the purification method, albeit with the caveat that this only accounts for the toluene soluble components of the crude reaction mixtures (see SI).

### Crystallographic Analysis of **α­(1)**_**2**_ Series

To verify the presence of the proposed intramolecular N···S interactions we sought to examine the **α­(1)** **_2_** series in the solid-state. Due to the poor alignment of the imine lone pair and the σ-hole of the sulfur atom any intramolecular interactions observed would be defined as attractive electrostatic interactions (due to the *cis*-effect)^40^ and not chalcogen bonding (where the N···S–C angle must be close to 180°).^22^ Vapor diffusion of petroleum ether into solutions of **α­(1b)** **_2_** (THF), **α­(1c)** **_2_** (DCM), **α­(1d)** **_2_** (THF), and **α­(1cc)** **_2_** (THF) produced yellow crystals of sufficient quality for single crystal X-ray diffraction. Figure 3 portrays ORTEP diagrams for single crystals of **α­(1b)** **_2_**, **α­(1c)** **_2_**, **α­(1d)** **_2_**, and **α­(1cc)** **_2_** with key atoms labeled (see SI for full details). In each case the intramolecular N···S distance (∼ 3 Å) falls within the sum of the van der Waals radii (∼ 3.3 Å).

**Figure 3:**
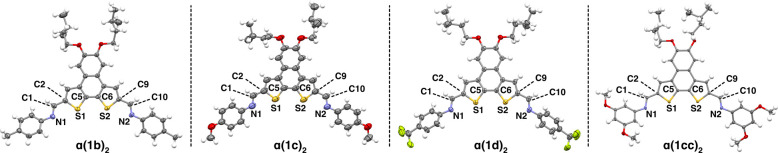
ORTEP diagrams (ellipsoids drawn at 50% probability) of **α­(1b)_2_**, **α­(1c)_2_**, **α­(1d)_2_**, and **α­(1cc)_2_**. Average select distances (Å): **α­(1b)_2_** N···S, 3.00; **α­(1c)_2_** N···S, 2.98; **α­(1d)_2_** N···S, 2.94; **α­(1cc)_2_** N···S, 3.05. Average select bond angles (°): **α­(1b)_2_** N–C–C–S, 0.6; **α­(1c)_2_** N–C–C–S, −2.5; **α­(1d)_2_** N–C–C–S, 4.9; **α­(1cc)**_2_ N–C–C–S, 1.2. Additional details given in the SI.

As anticipated the four-atom arrangement is too compact to allow for quality orbital overlap, with the N···S–C bond angle (∼ 140° in all cases) being mis-aligned for strong contact between imine lone pair and S–C σ-hole. However, the strength of the intramolecular N···S interaction is apparent due to the high degree of planarity of the four-atom arrangement, with the N–C–C–S dihedral being essentially planar (±5°). These metrics are comparable to known four-atom benzothienyl-aryl imine crystal structures^27–35^ and their benzothienyl-pyridyl analogues.^41–44^

Unfortunately, all attempts to crystallize **α­(1a)** **_2_** proved futile, with hydrolysis byproducts such as **α­(1a)­(CHO)** being isolated instead of the target molecule (see SI for crystallographic analysis of this particular byproduct). This byproduct is quite unique: the single imine linkage is orientated in the nonintramolecular N···S conformation while the aldehyde is cisoid to the interior sulfur (i.e., there is an intramolecular O···S contact with a distance of ∼ 3 Å). Overall, the **α­(1)** **_2_** series exhibits modest stability under ambient conditions, with decomposition via hydrolysis being detected for both the *m*-aryl and *p*-aryl derivatives after weeks of storage.

### Solution-State Analysis of **α­(1)**_**2**_ Series

With the presence of these intramolecular N···S interactions authenticated in the solid-state we sought to probe their propensity in the solution-state. In solution the imine linkages are proposed to freely rotate between conformer-1 and conformer-2 (Figure 2, top), with the NMR spectra correlating to the average conformation. Inspection of the ^1^H NMR spectra, shown in Figure 4, reveals that the entire **α­(1)** **_2_** series have well-resolved signals that are quite uniform in nature, suggesting rapid imine rotation on the NMR time scale. Inspection of resonances i and 2b, which are most impacted by the distribution between the two lowest energy rotamers (conformer-1 and conformer-2), highlight that these molecules are predominately in the same rotamer configuration: the gold highlighted signals of the imine proton have Δδ < 0.10 ppm and the gray highlighted signals of the interior thienyl 2b proton have Δδ < 0.20 ppm. This level of modulation of the 2b resonance is reasonable based on previous results with phenylene end-caps,^14^ with concentration effects having minimal impact on the observed chemical shift. This modulation of end-cap communication to the core can also be observed in the imine ^13^C resonance, which correlates well with the remote substituent Hammett parameter^45^ (Figure 4, inset). Aside from these resonances being in similar locations, it is difficult to surmise which conformation is preferred in solution, thus additional analysis is required to properly assign the dominant solution-state population.

**Figure 4:**
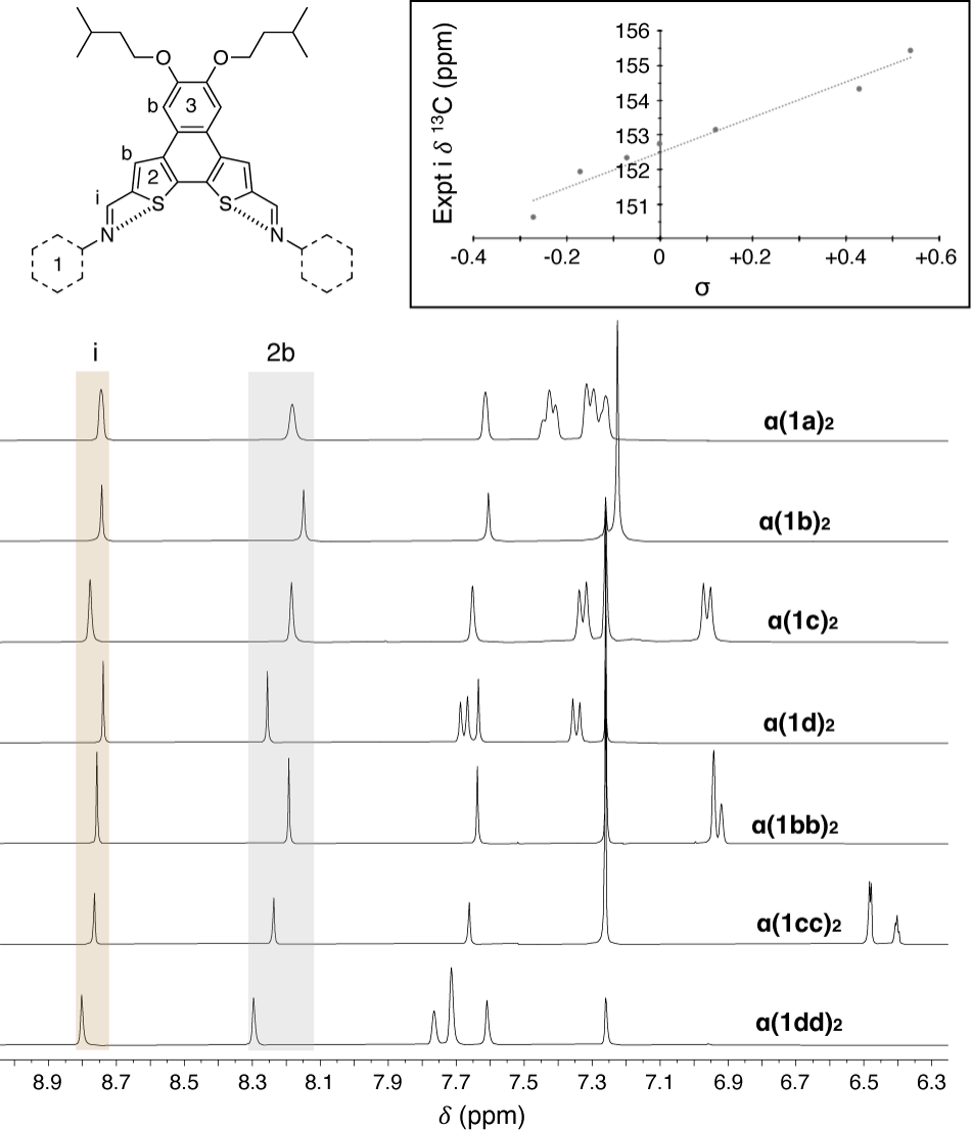
Partial ^1^H NMR spectra of the **α­(1)_2_** series (400 MHz, CDCl_3_). Gold highlighted i resonance covers Δδ < 0.1 ppm, gray highlighted 2b resonance covers Δδ < 0.2 ppm. Inset: Correlation of ^13^C imine resonance and Hammett parameter of remote substituent (R^2^ ≈ 0.96).

To ascertain which conformation is preferred in solution we explored the **α­(1)_2_** series computationally. To reduce variability the isopentyloxy groups were simplified to methoxy groups for the computational analysis – defined as α*(1)*_2_ – with the goal of keeping all the aromatic resonances in similar environments. Both conformer-1 and conformer-2 (generic representations of α*(1)*_2_ are shown in Figure 5, top) were treated under identical conditions in the gas phase to optimize their geometries and perform vibrational frequency calculations using the dispersion corrected ωB97-XD functional^46^ with a cc-pVDZ basis set,^47^ both of which are well-established for systems of this size and atom composition.^48^ Comparison of the resulting geometries reveals that conformer-1 is preferred by ∼ 3 kcal mol^–1^ over conformer-2 in all cases, with minimal perturbation from the remote substituents on the aryl amines (see SI for full details). The validity of these weak interactions (estimated to be ∼ 1.5 kcal mol^–1^ per N···S based on energetic difference between conformer-1 and conformer-2) was probed by natural bond orbital (NBO)^49,50^ analysis for conformer-1. The lone pair of the imine nitrogen, although mis-aligned for strong interaction with the σ-hole of the S–C bond, was found to be stabilizing by ∼ 1 kcal mol^–1^ per N···S interaction for the α*(1)*_2_ series with no dependence on the remote substituent (see SI for exact values obtained from NBO analysis of N···S–C interactions). While it is promising that this computational result matches the conformation observed via crystallography, the goal is to understand the propensity of N···S interactions in solution.

**Figure 5:**
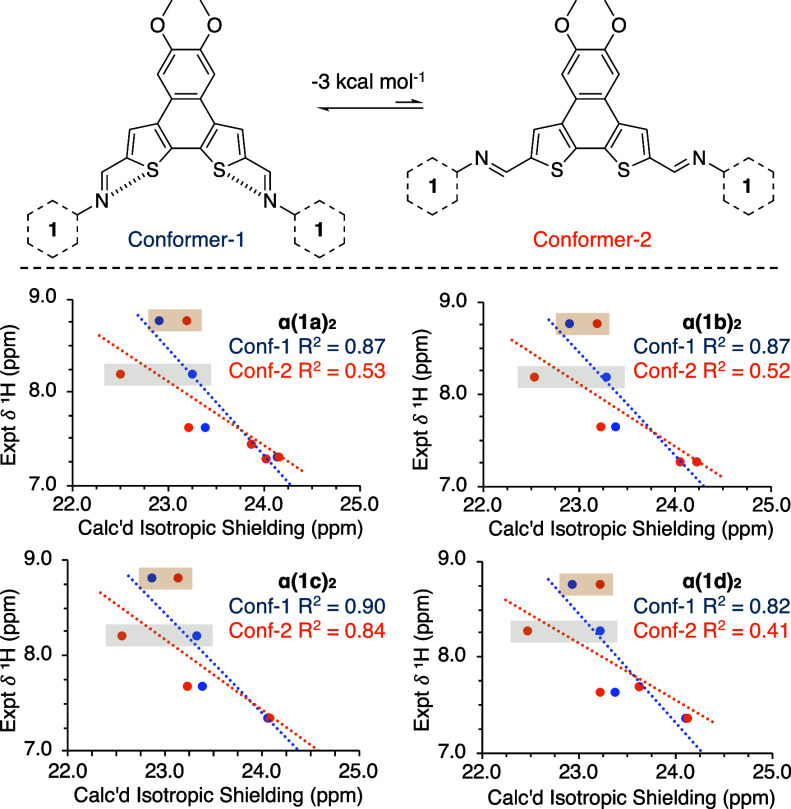
Computational analysis of *α­(1a-d)*_2_ series. Top: general representations of two available *E* conformations. Bottom: Experimental vs calculated ^1^H NMR plots reveal strong correlations for conformer-1 (blue) and poor fits for conformer-2 (orange). Gold and gray shaded regions represent the imine (i) and interior thienyl proton (2b). Computational method: PCM­(CHCl_3_)/ωB97-XD/cc-pVDZ.

The ^1^H and ^13^C spectra for the α*(1)*_2_ series were calculated utilizing the Gauge Including Atomic Orbitals (GIAO) protocol^51,52^ as implemented in Gaussian 16 with a polarizable continuum model (PCM)^53^ to ensure the calculated spectra are corrected for the experimental solvent used (CHCl_3_ as solvent, see SI for additional details).^54^ For comparison of the experimental and computed spectra we focused on the resonances extracted from ^1^H–^13^C HSQC spectra, allowing for both the ^1^H and ^13^C spectra to be used in the analysis.

The ^1^H spectra are vital in this approach: Figure 5 reveals that for the *p*-aryl imine series the calculated chemical shifts of conformer-1 are a quality fit for the observed resonances (average R^2^ ≈ 0.86), while conformer-2 is generally a poor fit (average R^2^ ≈ 0.57). The comparison of experimental ^13^C resonances with the calculated isotropic shieldings reveal quality fits for all resonances, a feature that was expected based on the carbons being in essentially the same environment in both conformer-1 and conformer-2 (see SI for full ^1^H and ^13^C computational analysis of both the *p*-aryl and *m*-aryl imines). In total, blending the observed experimental resonances and the calculated isotropic shieldings yields a solution-state preference for the N···S conformer-1 across the **α­(1)** **_2_** series.

### Photophysical Properties **α­(1)**_**2**_ Series

With the authenticated structures in hand our focus shifted to examining the photophysical properties of the **α­(1)_2_** series. Imines notoriously diminish the emission of their respective molecules due to energy loss via nonradiative decay pathways.^55^ With these systems displaying intramolecular N···S contacts we hypothesized this may allow for a modest emission response. UV–visible absorption spectra for the **α­(1)_2_** series, shown in Figures S59 and S60, have nearly identical vibronic structure that are comparable to previously reported phenylene-based end-caps,^8,9,14^ with the core difference being the n-π* low energy shoulder being less prominent (i.e., it appears mixed with the λ_max_ attributed to the π-π* transition). Table 1 details several key metrics obtained throughout the analysis, wherein the λ_max_ and estimated E_g,opt_ ^56^ reside in a tight range no matter the aryl amine substituent. The emission spectra of the **α­(1)_2_** series are quite distinct when comparing non-polar substituents to their polarizable counterparts. The non-polar derivatives **α­(1a)_2_**, **α­(1b)_2_**, and **α­(1bb)_2_** yield essentially no response (ϕ_PL_ < 0.1%). The other derivatives, whether having electron donating (**α­(1c)**_**2**_) or withdrawing (**α­(1d)_2_**, **α­(1cc)_2_**, and **α­(1dd)_2_**) substituents yield emission with modest vibronic structure with weak quantum yields. These emission spectra are substantially overlapped with the absorbance, indicative of an anti-Stokes shift. This is reminiscent of the triplet–triplet annihilation anti-Stokes shift^57^ observed by Yang and co-workers on BODIPY-based chromophores.^58^ Molecules displaying this type of emission response, even in low efficiencies, are of interest as bioimaging agents.^59^

**Table 1:** Photophysical Metrics of α(1)_2_ Series

**α(1)_2_**	ε^a^ (M^–1^ cm^–1^)	λ_abs,max_ ^b^ (nm)	E_g,opt_ ^c^ (eV)	λ_em_ ^d^ (nm)	ϕ_PL_
**α(1a)**_**2**_	1.02 × 10^4^	417	2.58	–	–
**α(1b)**_**2**_	7.61 × 10^3^	421	2.62	–	–
**α(1c)**_**2**_	1.55 × 10^3^	431	2.56	406	0.4%
**α(1d)**_**2**_	9.42 × 10^3^	420	2.60	468	0.2%
**α(1bb)**_**2**_	4.07 × 10^3^	419	2.62	–	–
**α(1cc)**_**2**_	3.32 × 10^4^	421	2.61	416	0.6%
**α(1dd)**_**2**_	7.40 × 10^3^	426	2.58	440	0.2%

a UV-visible and emission spectra were measured as solutions in dry, degassed toluene under inert atmosphere.

b Highest intensity peak above 315 nm.

c E_g,opt_ estimated using 0nset (Ref 56).

d λ_ex_ = 365 nm.

Recently the photoisomerization of imines from *E*-to-*Z* with visible light (405–450 nm) has been studied in detail by Greenfield and co-workers.^60–62^ These imine-systems (aryliminopyrazoles, AIPs) feature electron donating groups *ortho* to the aldehyde coupling partner. This orientation allows for temporary stabilization of the *Z* configuration upon photoexcitation, which then can be converted thermally to the more stable *E* configuration. Phenylene-thienyl imines have been observed to undergo rapid *E*/*Z* photoisomerization as well, with the maximum amount of photoconversion to *Z* being 20% after irradiating at 365 nm.^63^ During the course of analysis under standard conditions the **α­(1)** **_2_** series does not exhibit ^1^H resonances indicative of the *Z* configuration.

However, the **α­(1)_2_** series described in this report could conceivably undergo *E*-to-*Z* photoisomerization at λ_ex_ = 365 nm, therefore we carried out preliminary irradiation experiments followed by prompt ^1^H NMR acquisition. Electron deficient end-caps are known to induce photoisomerization in benzylimines,^64^ yet in **α­(1dd)_2_** the presence of the *Z* configuration of the imine proton (or any additional signals) was not detected. This result mirrors that of Barik and Skene with imine-linked fluorenes, whose polyaromatic hydrocarbon cores elevate the inversion barrier of photoisomerization such that it is not feasible under these conditions.^65^ The poor emission of imines is often derived from the low rotation barrier about the imine linkage,^66^ which is likely the reason for the large portion of nonradiative decay in these systems (e.g., computational results depict the energetic differences between conformer-1 and conformer-2 to be <3 kcal mol^–1^, which is far smaller than the photoisomerization inversion barrier).

## Conclusion

This series of di-imine functionalized α NDT building blocks represents a modular unit for photoactive molecules bearing dynamic linkages. By strategically placing the imines in close proximity to the interior sulfur atoms an attractive electrostatic interaction forms between the nitrogen lone pair and the σ-hole of the nearby S–C moiety; ultimately rigidifing the **α­(1)** **_2_** series such that their ^1^H NMR spectra are representative of a single conformation (conformer-1) in solution. This conformation was also observed in the solid-state for **α­(1b)** **_2_**, **α­(1c)** **_2_**, **α­(1d)** **_2_**, and **α­(1cc)** **_2_**, with an average intramolecular N···S distance of ∼ 3 Å. The remote substituents of the aryl amines modulate the imine ^13^C resonance in a predictable fashion that correlates with each substituents’ respective Hammett parameter. Photophysical analysis of the **α­(1)** **_2_** series depict a set of molecules that have modest molar absorptivities (>10^3^ M^–1^ cm^–1^) and poor quantum yields (<1%). These attributes – in combination with the observed anti-Stokes shifts in **α­(1c)** **_2_** and **α­(1cc** **)** **_2_** – depict these chromophores as being useful photoactive materials. Efforts to further modulate the photophysical properties of N···S rigidified bent NDTs in both small molecules and complex molecular systems are currently underway.

## Supplementary Material





## Data Availability

The data underlying this study are available in the published article and its Supporting Information.

## Notes

Experimental procedures, NMR and photophysical spectra, computational data, and crystallographic results (PDF)
Computational geometries (RTF)
