# Supplementary material for: Intramolecular Stabilization of Naphtho[2,1‑b:3,4‑b′]dithiophenes: Synthesis and Analysis of Imine-Based Chromophores
Source: J Org Chem. 2025 Dec 16;91(1):394–400. doi: 10.1021/acs.joc.5c02486 (PMC12797285; doi:10.1021/acs.joc.5c02486)
Supplement: Supplementary file 1 [file jo5c02486_si_001.pdf]

Intramolecular Stabilization of Naphtho[2,1-*b*:3,4-*b'*]dithiophenes: Synthesis and Analysis of Imine-Based Chromophores

Emmanuel B.A. Adusei,[a] Sarah Ibrahim,[a] Kara Jenneker,[a] Calvin D. Goldsmith,[a] Danielle Dragoi,[a] Matthias Zeller,[b] and Zacharias J. Kinney[a]*

Supporting Information

*email: kinney@oakland.edu

1)	Experimental Procedures	S2
2)	NMR Spectra	S7
3)	GPC Chromatograms	S38
4)	Photophysical Analysis of **α(1)₂**	S39
5)	Computational Analysis	S40
6)	Crystallographic Analysis	S47
7)	Author Contributions	S57
8)	References	S58

1) Experimental Procedures

Unless otherwise noted, all starting materials, reagents, and solvents were purchased from commercial sources and used without further purification. Anhydrous solvents (dichloromethane, hexanes, tetrahydrofuran, toluene) were purified using an Inert PureSolv solvent purification system equipped with an alumina column. 4Å molecular sieves were activated via vacuum oven (150 °C, >16 hours) overnight prior to use. Purification of all di-imine products was accomplished with a Shimadzu Nexera Gel Permeation Chromatography (GPC) equipped with a Shodex organic size exclusion column (100 Å styrogel stationary phase, viable molecular weight range of 400–6000 g/mol) and a toluene mobile phase (flow rate 5 mL/min). NMR spectra were measured on a Bruker Avance III 400 MHz spectrometer, with all samples referenced to the residual solvent signal. Structural assignments were made with additional information obtained from gCOSY, gHSQC, gHMBC, and gNOESY experiments. High resolution mass spectra were obtained by dissolving samples in acetonitrile and analyzed on a Shimadzu LCMS-9050 Q-TOF (ionization method: APCI+). Melting points were measured with a Stuart SMP10 melting point apparatus for **3**, **α**, and **α(CHO)₂**. The procedure for **2** is supplied below for clarity with reference to the original spectral analysis.[2,3] All procedures below were carried out multiple times with the yields given representing the median outcome of an experiment. Common solvent and reagent abbreviations: chloroform-*d* ($CDCl_3$), dichloromethane (DCM), ethyl acetate (EtOAc), tetrahydrofuran (THF), and *N*-bromosuccinimide (NBS).

Synthesis of precursors and ***α(CHO)₂***

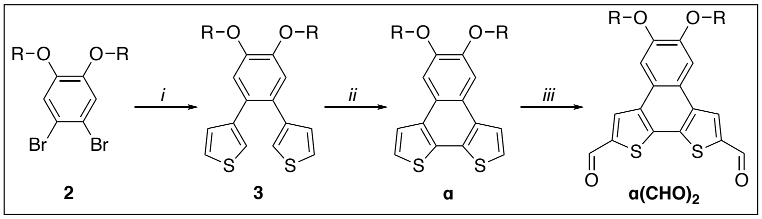

Scheme S1. Synthetic path to **α(CHO)₂**; details for conditions *i*, *ii*, and *iii* given below with their respective targets.

1,2-dibromo-4,5-di-isopentyloxybenzene, **2**

4,5-dibromocatechol (5.06 g, 18.9 mmol) was massed into a 250 mL round bottom flask and dissolved in 2-butanone (100 mL). A stir bar was added, followed by K_2CO_3 (8.00 g, 57.9 mmol) and a reflux condenser. Reaction was flushed with argon for 5 minutes, then 1-bromo-3-methylbutane (5.20 mL, 41.3 mmol) was added in one portion. Reaction temperature was then raised to 80 °C and allowed to stir. After 24 hours the reaction flask was cooled to RT and poured into H_2O (50 mL), extracted with EtOAc (2x50 mL), washed with brine (2x50 mL), dried over anhydrous Na_2SO_4, decanted, and concentrated to yield crude product. Purification via silica gel flash column with Hexanes:EtOAc (49:1) as mobile phase afforded 1,2-dibromo-4,5-di-isopentyloxybenzene (**2**) as a colorless oil, 6.1899 g (80%). Spectral details match literature values.[2,3]

1,2-di(3-thienyl)-4,5-di-isopentyloxybenzene, **3**

A 100 mL Schlenk tube was charged with **2** (3.156 g, 7.72 mmol), 3-thiophene boronic acid (3.006 g, 23.5 mmol), and $Pd(PPh_3)_2Cl_2$ (833 mg, 1.19 mmol), and a stir bar added. The reaction flask was then backfilled with argon (x3), after which 40 mL dry THF (30 mL) and degassed 2 M K_2CO_3 (15.4 mL, 30.8 mmol) were added under positive pressure of argon. The tube was sealed under argon and allowed to stir in an oil bath at 80 °C overnight. After overnight stirring the reaction tube was cooled to RT, poured into H_2O (25 mL), extracted with EtOAc (2x50 mL), washed with brine (2x50 mL), dried over anhydrous Na_2SO_4, decanted, and concentrated to yield crude product. The crude product was purified via silica gel flash column with Hexanes:EtOAc (49:1) mobile phase followed by recrystallization from ethanol to afford 1,2-di(3-thienyl)-4,5-di-isopentyloxybenzene (**3**) as a white solid, 1.9476 g (70%): mp 64-67 °C; ^1H NMR (400 MHz, $CDCl_3$) δ 7.17 (dd, *J* = 4.9, 3.0 Hz, 2H), 7.02 (dd, *J* = 3.0, 1.3 Hz, 2H), 6.97 (s, 2H), 6.78 (dd, *J* = 4.9, 1.3 Hz, 2H), 4.08 (t, *J* = 6.7 Hz, 4H), 1.87 (dq, *J* = 13.3, 6.7 Hz, 2H), 1.75 (q, *J* = 6.7 Hz, 4H), 0.98 (d, *J* = 6.6 Hz, 12H); $^{13}C\{^1H\}$ NMR (101 MHz, $CDCl_3$) δ 148.4, 142.0, 129.1, 128.0, 124.5, 122.3, 115.7, 67.9, 38.1, 25.1, 22.6; HRMS (APCI+) calcd for $C_{24}H_{31}O_2S_2$ $(M+H)^+$ 415.1765, found 415.1768.

5,6-*bis*(isopentyloxy)naphtho[2,1-*b*:3,4-*b′*]dithiophene, **α**

A 500 mL Schlenk flask was charged with **3** (439 mg, 1.06 mmol) and a stir bar, then was sparged with argon for 10 minutes. After 10 minutes dry DCM (42 mL) was added and the reaction flask was equipped with a pressure equalizing funnel under positive pressure of argon. The reaction flask was then cooled to 0 °C and equilibrated for 15 minutes. A solution of $FeCl_3$ (362 mg, 2.23 mmol) dissolved in $MeNO_2$ (9 mL) was added to the pressure equalizing funnel

and added dropwise over five minutes to the reaction flask. After complete addition of the $FeCl_3$ solution the ice bath was removed and the reaction was allowed to come to RT. After 1 hour the reaction was quenched with MeOH (90 mL $MeNO_2$) and concentrated. The crude reaction was resuspended in DCM (50 mL) and H_2O (50 mL), extracted with DCM (2x50 mL), washed with brine (2x50 mL), dried over anhydrous Na_2SO_4, decanted, and concentrated to yield crude product. The residue was purified via silica gel flash column with Hexanes:DCM (4:1) mobile phase to give 5,6-*bis*(isopentyloxy)naphtho[2,1-*b*:3,4-*b'*]dithiophene (**α**) as an off-white solid, 190 mg (43%): mp 125-126 °C; ^{1}H NMR (400 MHz, $CDCl_3$) δ 7.86 (d, *J* = 5.3 Hz, 2H), 7.69 (s, 2H), 7.45 (d, *J* = 5.3 Hz, 2H), 4.25 (t, *J* = 6.6 Hz, 4H), 1.99 (dt, *J* = 13.3, 6.6 Hz, 2H), 1.87 (q, *J* = 6.7 Hz, 4H), 1.07 (d, *J* = 6.6 Hz, 12H); ^{13}C{^{1}H} NMR (101 MHz, $CDCl_3$) δ 148.8, 133.8, 130.3, 123.3, 122.6, 122.4, 106.7, 67.6, 38.0, 25.2, 22.7; HRMS (APCI+) calcd for $C_{24}H_{29}O_2S_2$ $(M+H)^+$ 413.1608, found 413.1609.

5,6-*bis*(isopentyloxy)-2,9-di-(formyl)naphtho[2,1-*b*:3,4-*b'*]dithiophene, **α(CHO)₂**

A 100 mL Schlenk flask was massed with **α** (0.58 mmol), a stir bar, dry THF (20 mL) and sparged with argon for 15 minutes. The flask was cooled to -78 °C and allowed to equilibrate for 20 minutes while being shielded from light. *n*-Butyllithium (0.76 mL, 1.22 mmol) was added dropwise to the reaction and stirred for 30 minutes. Degassed dry DMF (0.40 mL, 5.20 mmol) was added dropwise and allowed to stir under inert atmosphere of argon for 30 minutes. The dry ice bath was removed and the reaction was warmed to RT, then was quenched with 5 mL dilute HCl solution after 40 minutes, poured into H_2O (15 mL), extracted with EtOAc (2x50 mL), washed with brine (2x50 mL), dried over anhydrous Na_2SO_4, decanted, and concentrated. The crude product was recrystallized from toluene to yield 5,6-*bis*(isopentyloxy)-2,9-di-(formyl)naphtho[2,1-*b*:3,4-*b'*]dithiophene (**α(CHO)$_2$**) as a yellow solid, 114 mg (42%): mp 210 °C (decomposition); ^{1}H NMR (400 MHz, $CDCl_3$) δ 10.09 (s, 2H), 8.23 (s, 2H), 7.32 (s, 2H), 4.19 (t, *J* = 6.6 Hz, 4H), 1.93 (dq, *J* = 13.0, 6.6 Hz, 2H), 1.84 (q, *J* = 6.6 Hz, 4H), 1.05 (d, *J* = 6.5 Hz, 12H); ^{13}C{^{1}H} NMR (101 MHz, $CDCl_3$) δ 183.4, 151.3, 141.6, 141.2, 131.7, 131.3, 122.6, 105.9, 67.8, 37.6, 25.3, 22.7.; HRMS (APCI+) calcd for $C_{26}H_{29}O_4S_2$ $(M+H)^+$ 469.1507, found 469.1514.

*Synthesis of **α(1)$_2$** Series*

Purification of all di-imine **α(1)$_2$** products was accomplished via a semi-preparative Shimadzu Nexera Gel Permeation Chromatography (GPC) equipped with a Shodex organic size exclusion column (100 Å styrogel stationary phase, rapid preparative column dimensions 20 x 600 mm, viable molecular weight range of 400–6000 g/mol) and a toluene mobile phase (flow rate 5 mL/min).

5,6-*bis*(isopentyloxy)-2,9-di-(*N*-(phenyl)methaniminyl) naphtho[2,1-*b*:3,4-*b'*]dithiophene, **α(1a)$_2$**

Conditions *i*: A 6-dram vial was massed with **α(CHO)$_2$** (22.3 mg, 0.05 mmol), deacidified $CHCl_3$ (to 10 mM NDT, 5 mL), and 4 Å activated molecular sieves. After thoroughly mixing aniline (**1a**, 0.010 mL, 0.11 mmol) and TFA (0.071 mL, 4.76×10^{-3} mmol) were added. The reaction vial was capped, inverted several times to mix, and left undisturbed overnight (>16 hours). The reaction mixture was transferred into a clean 100 mL round bottom flask containing 1 mL triethylamine to quench the residual acid and concentrated to yield the crude product. **α(1a)$_2$** was isolated as a yellow solid, 13.6 mg (46%).

Conditions *ii*: A 6-dram vial was massed with **α(CHO)$_2$** (22.6 mg, 0.05 mmol), deacidified $CHCl_3$ (to 10 mM NDT, 5 mL), and 4 Å molecular sieves. After thoroughly mixing aniline (**1a**, 0.014 mL, 0.15 mmol), $Sc(OTf)_3$ (6.9 mg, 0.014 mmol), and MeCN (5 % of $CHCl_3$) were added. The reaction vial was capped, inverted several times to mix, and left undisturbed overnight (>16 hours). The reaction mixture was transferred into a clean 100 mL round bottom flask containing 1 mL triethylamine to quench the residual acid and concentrated to yield the crude product. **α(1a)$_2$** was obtained as a yellow solid, 11.7 mg (39%).

Analysis **α(1a)$_2$**: ^{1}H NMR (400 MHz, $CDCl_3$) δ 8.75 (s, 2H), 8.18 (s, 2H), 7.61 (s, 2H), 7.46 – 7.38 (m, 4H), 7.33 – 7.28 (m, 4H), 7.28 – 7.22 (m, 2H), 4.24 (t, *J* = 6.6 Hz, 4H), 1.96 (dp, *J* = 13.1, 6.6 Hz, 2H), 1.85 (q, *J* = 6.6 Hz, 4H), 1.04 (d, *J* = 6.6 Hz, 12H); ^{13}C{^{1}H} NMR (101 MHz, $CDCl_3$) δ 152.7, 151.0, 149.5, 141.8, 135.3, 132.5, 129.2, 127.3, 126.5, 122.8, 121.2, 106.3, 67.7, 38.0, 25.3, 22.7; HRMS (APCI+) calcd for $C_{38}H_{39}N_2O_2S_2$ $(M+H)^+$ 619.2452, found 619.2440.

5,6-*bis*(isopentyloxy)-2,9-di-(*N*-(4-methylphenyl)methaniminyl) naphtho[2,1-*b*:3,4-*b'*]dithiophene, **α(1b)$_2$**

Conditions *i*: A 6-dram vial was massed with **α(CHO)$_2$** (20.0 mg, 0.043 mmol), deacidified $CHCl_3$ (to 10 mM NDT, 4.5 mL), and 4 Å molecular sieves. After thoroughly mixing *p*-toluidine (**1b**, 11.2 mg, 0.11 mmol) and TFA (0.03 mL, 4.2×10^{-3} mmol) were added. The reaction vial was capped, inverted several times to mix, and left undisturbed

overnight (>16 hours). The reaction mixture was transferred into a clean 100 mL round bottom flask containing 1 mL triethylamine to quench the residual acid and concentrated to yield the crude product. **α(1b)₂** was isolated as a yellow solid, 15.6 mg (57%).

Conditions *ii*: A 6-dram vial was massed with **α(CHO)₂** (22.6 mg, 0.05 mmol), deacidified $CHCl_3$ (to 10 mM NDT, 5 mL), and 4 Å molecular sieves. After thoroughly mixing *p*-toluidine (**1b**, 12.2 mg, 0.11 mmol), $Sc(OTf)_3$ (4.7 mg, 9.55 x 10^{-3} mmol), and MeCN (5 % of $CHCl_3$) were added. The reaction vial was capped, inverted several times to mix, and left undisturbed for overnight (>16 hours). The reaction mixture was transferred into a clean 100 mL round bottom flask containing 1 mL triethylamine to quench the residual acid and concentrated to yield the crude product. **α(1b)₂** was isolated as a yellow solid, 13.5 mg (43%).

Analysis **α(1b)₂**: ^{1}H NMR (400 MHz, $CDCl_3$) δ 8.74 (s, 2H), 8.15 (s, 2H), 7.61 (s, 2H), 7.23 (s, 8H), 4.23 (t, *J* = 6.6 Hz, 4H), 2.39 (s, 6H), 1.96 (dp, *J* = 13.2, 6.6 Hz, 2H), 1.84 (q, *J* = 6.6 Hz, 4H), 1.04 (d, *J* = 6.6 Hz, 12H); ^{13}C{^{1}H} NMR (101 MHz, $CDCl_3$) δ 151.9, 149.4, 148.4, 141.9, 136.4, 135.3, 132.4, 129.8, 127.0, 122.8, 121.1, 106.3, 67.7, 38.0, 25.3, 22.7, 21.1; HRMS (APCI+) calcd for $C_{40}H_{43}N_2O_2S_2$ $(M+H)^+$ 647.2765, found 647.2768.

5,6-*bis*(isopentyloxy)-2,9-di-(*N*-(4-methoxyphenyl)methaniminyl)naphtho[2,1-*b*:3,4-*b'*]dithiophene, **α(1c)₂**
Conditions *i*: A 6-dram vial was massed with **α(CHO)₂** (25.0 mg, 0.05 mmol), deacidified $CHCl_3$ (to 10 mM NDT, 5 mL), and 4 Å molecular sieves. After thoroughly mixing *p*-anisidine (**1c**, 15.4 mg, 0.13 mmol) and TFA (0.04 mL, 5.6 x 10^{-3} mmol) were added. The reaction vial was capped, inverted several times to mix, and left undisturbed overnight (>16 hours). The reaction mixture was transferred into a clean 100 mL round bottom flask containing 1 mL triethylamine to quench the residual acid and concentrated to yield the crude product. **α(1c)₂** was obtained as a yellow solid, 10.9 mg (30%).

Conditions *ii*: A 6-dram vial was massed with **α(CHO)₂** (22.5 mg, 0.05 mmol), deacidified $CHCl_3$ (to 10 mM NDT, 5 mL), and 4 Å molecular sieves. After thoroughly mixing *p*-anisidine (**1c**, 14.1 mg, 0.11 mmol), $Sc(OTf)_3$ (6.2 mg, 0.013 mmol), and MeCN (5 % of $CHCl_3$) were added. The reaction vial was capped, inverted several times to mix, and left undisturbed overnight (>16 hours). The reaction mixture was transferred into a clean 100 mL round bottom flask containing 1 mL triethylamine to quench the residual acid and concentrated to yield the crude product. **α(1c)₂** was isolated as a yellow solid, 14.8 mg (45%).

Analysis **α(1c)₂**: ^{1}H NMR (400 MHz, $CDCl_3$) δ 8.78 (s, 2H), 8.18 (s, 2H), 7.65 (s, 2H), 7.33 (d, *J* = 8.3 Hz, 4H), 6.96 (d, *J* = 8.6 Hz, 4H), 4.25 (t, *J* = 6.8 Hz, 4H), 3.86 (s, 6H), 2.03 – 1.92 (m, 2H), 1.85 (q, *J* = 6.6 Hz, 4H), 1.04 (d, *J* = 6.4 Hz, 12H); ^{13}C{^{1}H} NMR (101 MHz, $CDCl_3$) δ 158.7, 150.6, 149.4, 143.9, 142.1, 135.3, 132.3, 126.6, 122.8, 122.5, 114.5, 106.4, 67.8, 55.5, 38.0, 25.3, 22.7; HRMS (APCI+) calcd for $C_{40}H_{43}N_2O_4S_2$ $(M+H)^+$ 679.2664, found 679.2668.

5,6-*bis*(isopentyloxy)-2,9-di-(*N*-(4-trifluoromethylphenyl)methaniminyl)naphtho[2,1-*b*:3,4-*b'*]dithiophene, **α(1d)₂**
Conditions *i*: A 6-dram vial was massed with **α(CHO)₂** (20.0 mg, 0.043 mmol), deacidified $CHCl_3$ (to 10 mM NDT, 4.5 mL), and 4 Å molecular sieves. After thoroughly mixing 4-aminobenzotrifluoride (**1d**, 15.1 mg, 0.094 mmol) and TFA (0.03 mL, 4.2 μmol) were added. The reaction vial was capped, inverted several times to mix, and left undisturbed overnight (>16 hours). The reaction mixture was transferred into a clean 100 mL round bottom flask containing 1 mL triethylamine to quench the residual acid and concentrated to yield the crude product. **α(1d)₂** was obtained as a yellow solid, 6 mg (2%).

Conditions *ii*: A 6-dram vial was massed with **α(CHO)₂** (22.7 mg, 0.05 mmol), deacidified $CHCl_3$ (to 10 mM NDT, 5 mL), and 4 Å molecular sieves. After thoroughly mixing 4-aminobenzotrifluoride (**1d**, 23.4 mg, 0.15 mmol), $Sc(OTf)_3$ (5.4 mg, 0.011 mmol), and MeCN (5 % of $CHCl_3$) were added. The reaction vial was capped, inverted several times to mix, and left undisturbed overnight (>16 hours). The reaction mixture was transferred into a clean 100 mL round bottom flask containing 1 mL triethylamine to quench the residual acid and concentrated to yield the crude product. **α(1d)₂** was isolated as a yellow solid, 15.1 mg (41%).

Conditions *iii*: A 50 mL round bottom flask was massed with **α(CHO)₂** (20.8 mg, 0.05 mmol), 4-aminobenzotrifluoride (**1d**, 18.2 mg, 0.11 mmol), toluene (to 10 mM NDT, 5 mL), acetic acid (0.03 mL, 4.43 x 10^{-3} mmol), and a stir bar. Reaction was left to stir vigorously for 10 minutes under argon, and heated until reflux with dean-stark trap for >16 hours. Reaction was cooled and transferred into a clean 100 mL round bottom flask containing

1 mL triethylamine to quench the residual acetic acid and concentrated to yield the crude product. **α(1d)₂** was obtained as a yellow solid, 15.6 mg (47%).

Analysis **α(1d)$_2$**: ^{1}H NMR (400 MHz, $CDCl_3$) δ 8.74 (s, 2H), 8.26 (s, 2H), 7.68 (d, *J* = 8.3 Hz, 4H), 7.63 (s, 2H), 7.35 (d, *J* = 8.1 Hz, 4H), 4.24 (t, *J* = 6.6 Hz, 4H), 1.95 (dq, *J* = 13.1, 6.6 Hz, 2H), 1.85 (q, *J* = 6.7 Hz, 4H), 1.04 (d, *J* = 6.5 Hz, 12H); ^{13}C{^{1}H} NMR (101 MHz, $CDCl_3$) δ 154.3 (q, *J* = 3.7 Hz), 154.0, 149.7, 141.4, 135.6, 132.9, 128.4, 128.4, 128.1, 125.6, 122.9, 121.3, 106.3, 67.8, 37.9, 25.3, 22.7, 1.0; HRMS (APCI+) calcd for $C_{40}H_{37}F_6N_2O_2S_2$ $(M+H)^+$ 755.2200, found 755.2210.

5,6-*bis*(isopentyloxy)-2,9-di-(*N*-(4-trifluoromethylphenyl)methaniminyl)naphtho[2,1-*b*:3,4-*b'*]dithiophene, **α(1bb)$_2$**
Conditions *i*: A 6-dram vial was massed with **α(CHO)$_2$** (21.7 mg, 0.05 mmol), deacidified $CHCl_3$ (to 10 mM NDT, 5 mL), and 4 Å molecular sieves. After thoroughly mixing 3,5-dimethylaniline (**1bb**, 12.6 mg, 0.11 mmol) and TFA (0.03 mL, 4.76 x 10^{-3} mmol) were added. The reaction vial was capped, inverted several times to mix, and left undisturbed overnight (>16 hours). The reaction mixture was transferred into a clean 100 mL round bottom flask containing 1 mL triethylamine to quench the residual acid and concentrated to yield the crude product. **α(1bb)$_2$** was obtained as a yellow solid, 4.1 mg (13%).

Conditions *ii*: To a 6-dram vial was massed with **α(CHO)$_2$** (22.2 mg, 0.05 mmol), deacidified $CHCl_3$ (to 10 mM NDT, 5 mL), and 4 Å molecular sieves. After thoroughly mixing 3,5-dimethylaniline (**1bb**, 13.6 mg, 0.11 mmol), $Sc(OTf)_3$ (5.0 mg, 0.010 mmol), and MeCN (5 % of $CHCl_3$) were added. The reaction vial was capped, inverted several times to mix, and left undisturbed overnight (>16 hours). The reaction mixture was transferred into a clean 100 mL round bottom flask containing 1 mL triethylamine to quench the residual acid and concentrated to yield the crude product. **α(1bb)$_2$** was obtained as a yellow solid, 10.7 mg (33%).

Conditions *iii*: A 50 mL round bottom flask was massed with **α(CHO)$_2$** (21.3 mg, 0.05 mmol), 3,5-dimethylaniline (**1bb**, 12.2 mg, 0.10 mmol), toluene (to 10 mM NDT, 5 mL), acetic acid (0.020 mL, 5.06 x 10^{-3} mmol), and a stir bar. Reaction was left to stir vigorously for 10 minutes under argon, and heated until reflux with dean-stark trap for >16 hours. Reaction was allowed to cool and transferred into a clean 100 mL round bottom flask containing 1 mL triethylamine to quench the residual acetic acid and concentrated to yield the crude product. **α(1bb)$_2$** was isolated as a yellow solid, 12.1 mg (39%).

Analysis **α(1bb)$_2$**: ^{1}H NMR (400 MHz, $CDCl_3$) δ 8.76 (s, 2H), 8.19 (s, 2H), 7.64 (s, 2H), 6.94 (s, 4H), 6.92 (s, 2H), 4.24 (t, *J* = 6.6 Hz, 4H), 2.37 (s, 12H), 1.96 (dt, *J* = 13.2, 6.6 Hz, 2H), 1.85 (q, *J* = 6.7 Hz, 4H), 1.04 (d, *J* = 6.5 Hz, 12H).; ^{13}C{^{1}H} NMR (101 MHz, $CDCl_3$) δ 152.3, 151.0, 149.4, 141.9, 138.9, 135.3, 132.5, 128.2, 127.1, 122.8, 118.9, 106.3, 67.7, 38.0, 25.3, 22.7, 21.3; HRMS (APCI+) calcd for $C_{42}H_{47}N_2O_2S_2$ $(M+H)^+$ 675.3078, found 675.3087.

5,6-*bis*(isopentyloxy)-2,9-di-(*N*-(4-trifluoromethylphenyl)methaniminyl)naphtho[2,1-*b*:3,4-*b'*]dithiophene, **α(1cc)$_2$**
Conditions *i*: A 6-dram vial was massed with **α(CHO)$_2$** (21.9 mg, 0.05 mmol), deacidified $CHCl_3$ (to 10 mM NDT, 5 mL), and 4 Å molecular sieves. After thoroughly mixing 3,5-dimethoxylaniline (**1cc**, 16.3 mg, 0.11 mmol) and TFA (0.027 mL, 4.59 x 10^{-3} mmol) were added. The reaction vial was capped, inverted several times to mix, and left undisturbed overnight (>16 hours). The reaction mixture was transferred into a clean 100 mL round bottom flask containing 1 mL triethylamine to quench the residual acid and concentrated to yield the crude product. **α(1cc)$_2$** was obtained as a yellow solid, 4.8 mg (14%).

Conditions *ii*: A 6-dram vial was massed with **α(CHO)$_2$** (22.4 mg, 0.05 mmol), deacidified $CHCl_3$ (to 10 mM NDT, 5 mL), and 4 Å molecular sieves. After thoroughly mixing 3,5-dimethoxylaniline (**1cc**, 16.8 mg, 0.12 mmol), $Sc(OTf)_3$ (5.5 mg, 0.01 mmol), and MeCN (5 % of $CHCl_3$) were added. The reaction vial was capped, inverted several times to mix, and left undisturbed overnight (>16 hours). The reaction mixture was transferred into a clean 100 mL round bottom flask containing 1 mL triethylamine to quench the residual acid and concentrated to yield the crude product. **α(1cc)$_2$** was obtained as a yellow solid, 4.2 mg (12%).

Conditions *iii*: A 50 mL round bottom flask was massed with **α(CHO)$_2$** (22.8 mg, 0.05 mmol), 3,5-dimethoxylaniline (**1cc**, 16.8 mg, 0.11 mmol), toluene (to 10 mM NDT, 5 mL), acetic acid (0.020 mL, 5.06 x 10^{-3} mmol), and a stir bar. Reaction was left to stir vigorously for 10 minutes under argon, and heated until reflux with dean-stark trap for >16 hours. Reaction cooled and transferred into a clean 100 mL round bottom flask containing 1 mL triethylamine to

quench the residual acetic acid and concentrated to yield the crude product. **α(1cc)₂** was isolated as a yellow solid, 21.3 mg (59%).

Analysis **α(1cc)$_2$**: ^{1}H NMR (400 MHz, $CDCl_3$) δ 8.77 (s, 2H), 8.24 (s, 2H), 7.66 (s, 2H), 6.48 (d, J = 2.2 Hz, 4H), 6.40 (t, J = 2.2 Hz, 2H), 4.25 (t, J = 6.6 Hz, 4H), 3.85 (s, 12H), 1.95 (dq, J = 13.1, 6.5 Hz, 2H), 1.85 (q, J = 6.6 Hz, 4H), 1.04 (d, J = 6.6 Hz, 12H); ^{13}C{^{1}H} NMR (101 MHz, $CDCl_3$) δ 161.3, 153.1, 153.0, 149.5, 141.6, 135.4, 132.7, 127.6, 122.9, 106.3, 99.3, 99.0, 67.7, 55.5, 38.0, 25.3, 22.7; HRMS (APCI+) calcd for $C_{42}H_{47}N_2O_6S_2$ $(M+H)^+$ 739.2875, found 739.2881.

5,6-*bis*(isopentyloxy)-2,9-di-(*N*-(4-trifluoromethylphenyl)methaniminyl)naphtho[2,1-*b*:3,4-*b'*]dithiophene, **α(1dd)$_2$**
Conditions *ii*: A 6-dram vial was massed with **α(CHO)$_2$** (22.1 mg, 0.05 mmol), deacidified $CHCl_3$ (to 10 mM NDT, 5 mL), and 4 Å molecular sieves. After thoroughly mixing 3,5-*bis*(trifluoromethyl)aniline (**1dd**, 43.5 mg, 0.19 mmol), $Sc(OTf)_3$ (6.0 mg, 0.01 mmol), and MeCN (5 % of $CHCl_3$) were added. The reaction vial was capped, inverted several times to mix, and left undisturbed overnight (>16 hours). The reaction mixture was transferred into a clean 100 mL round bottom flask containing 1 mL triethylamine to quench the residual acid and concentrated to yield the crude product. **α(1dd)$_2$** was isolated as a yellow solid, 8.8 mg (21%).

Conditions *iii*: A 50 mL round bottom flask was massed with **α(CHO)$_2$** (21.3 mg, 0.05 mmol), 3,5-*bis*(trifluoromethyl)aniline (**1dd**, 26.2 mg, 0.11 mmol), toluene (to 10 mM NDT, 5 mL), acetic acid (0.017 mL, 4.52 x 10^{-3} mmol), and a stir bar. Reaction was left to stir vigorously for 10 minutes under argon, and heated until reflux with dean-stark trap for >16 hours. Reaction was cooled and transferred into a clean 100 mL round bottom flask containing 1 mL triethylamine to quench the residual acetic acid and concentrated to yield the crude product. **α(1dd)$_2$** was obtained as a yellow solid, 2.5 mg (6%).

Analysis **α(1dd)$_2$**: ^{1}H NMR (400 MHz, $CDCl_3$) δ 8.80 (s, 2H), 8.30 (s, 2H), 7.77 (s, 2H), 7.71 (s, 4H), 7.61 (s, 2H), 4.23 (t, J = 6.7 Hz, 4H), 1.94 (dt, J = 13.3, 6.7 Hz, 2H), 1.84 (q, J = 6.7 Hz, 4H), 1.04 (d, J = 6.5 Hz, 12H); ^{13}C{^{1}H} NMR (101 MHz, $CDCl_3$) δ 155.4, 152.2, 149.8, 140.9, 135.7, 133.1, 132.8 (q, J = 33.6 Hz), 129.4, 122.3 (q, J = 273.2 Hz), 121.4 (d, J = 3.6 Hz), 119.6 (t, J = 3.3 Hz), 106.1, 67.8, 37.9, 25.3, 22.7; HRMS (APCI+) calcd for $C_{42}H_{34}F_{12}N_2O_2S_2$ $(M+H)^+$ 891.1948, found 891.1935.

Table S1. Yield Summation, α(1)$_2$ Series

Derivative	Conditions *i*<br>TFA, $CHCl_3$, Mol. Sieves	Conditions *ii*<br>$Sc(OTf)_3$, MeCN, $CHCl_3$, Mol. Sieves	Conditions *iii*<br>Toluene, AcOH, Δ
**α(1a)$_2$**	46%	39%	–
**α(1b)$_2$**	57%	43%	–
**α(1c)$_2$**	30%	45%	–
**α(1d)$_2$**	2%	41%	47%
**α(1bb)$_2$**	13%	33%	39%
**α(1cc)$_2$**	14%	12%	59%
**α(1dd)$_2$**	–	21%	6%

2) NMR Spectra
Precursors and **α(CHO)₂**

1,2-di(3-thienyl)-4,5-di-isopentyloxybenzene, **3**

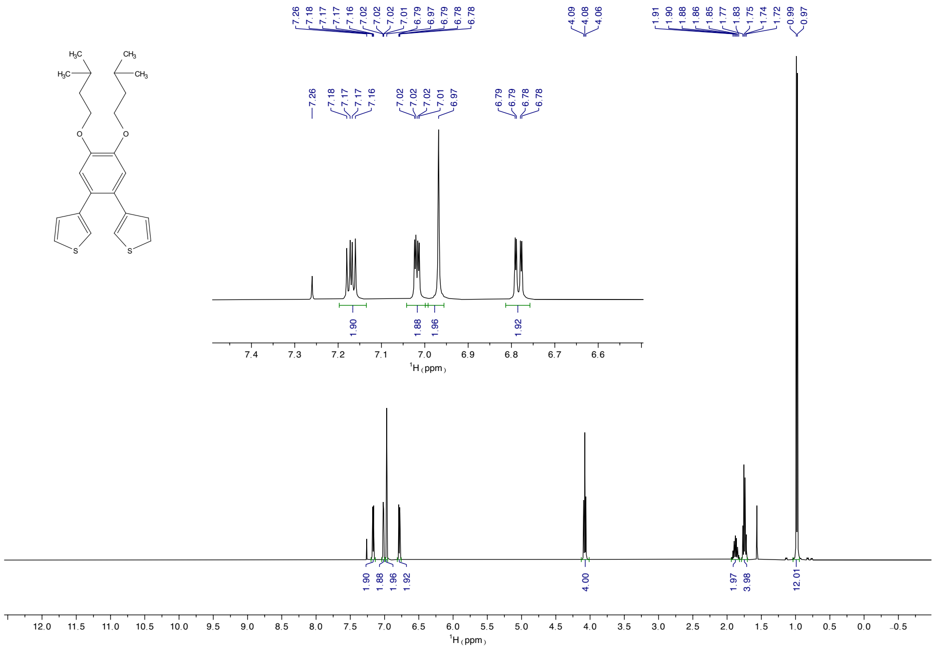

Figure S1. ^{1}H NMR spectrum of **3** ($CDCl_3$, 400 MHz, 298 K).

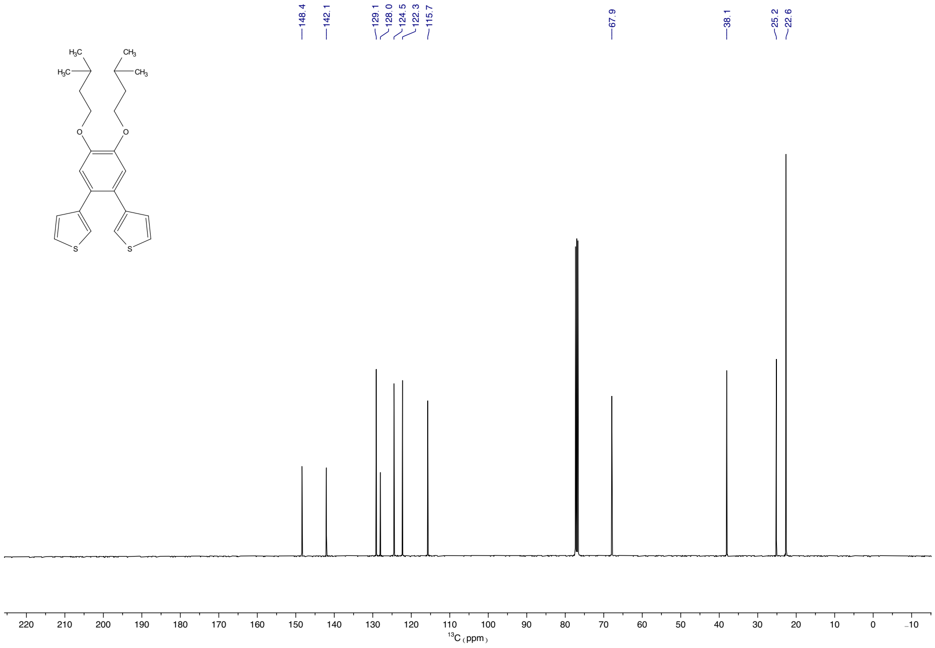

Figure S2. $^{13}C\{^{1}H\}$ NMR spectrum of **3** ($CDCl_3$, 400 MHz, 298 K).

5,6-bis(isopentyloxy)naphtho[2,1-*b*:3,4-*b'*]dithiophene, **α**

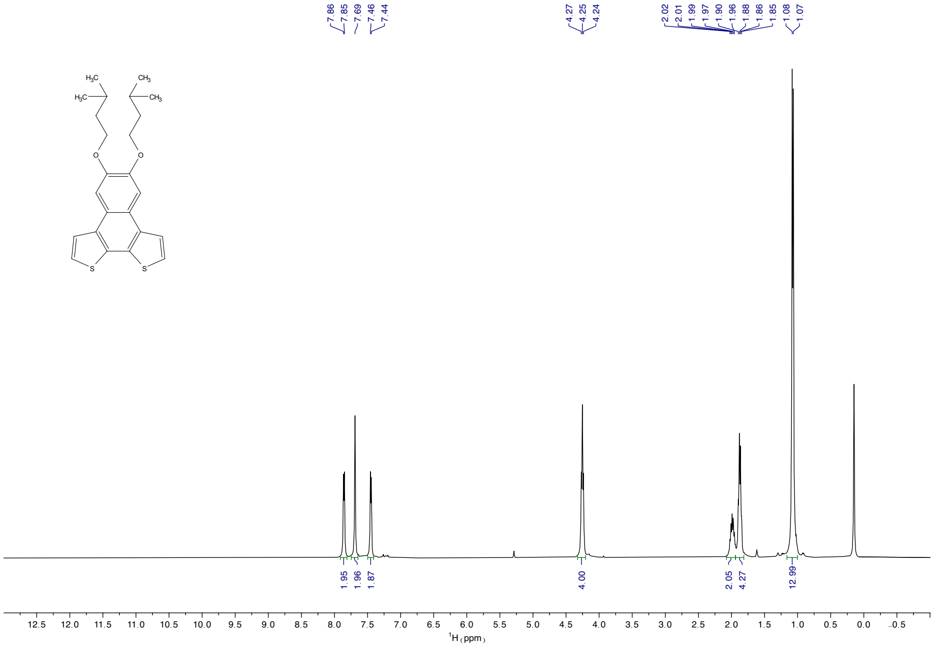

Figure S3. ^{1}H NMR spectrum of **α** ($CDCl_3$, 400 MHz, 298 K).

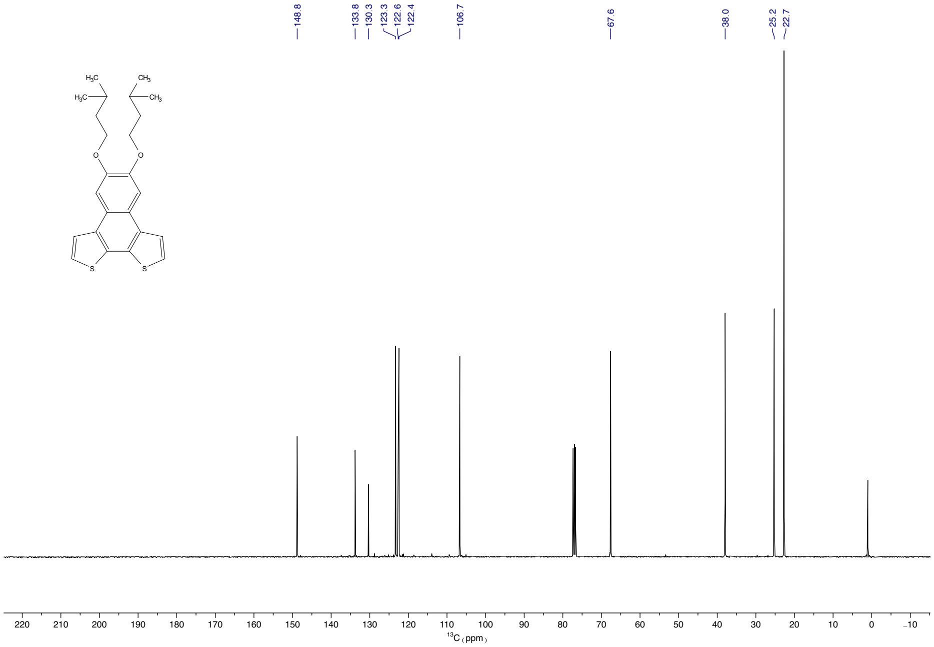

Figure S4. $^{13}C\{^{1}H\}$ NMR spectrum of **α** ($CDCl_3$, 101 MHz, 298 K).

5,6-bis(isopentyloxy)-2,9-diformylnaphtho[2,1-*b*:3,4-*b'*]dithiophene, **α(CHO)₂**

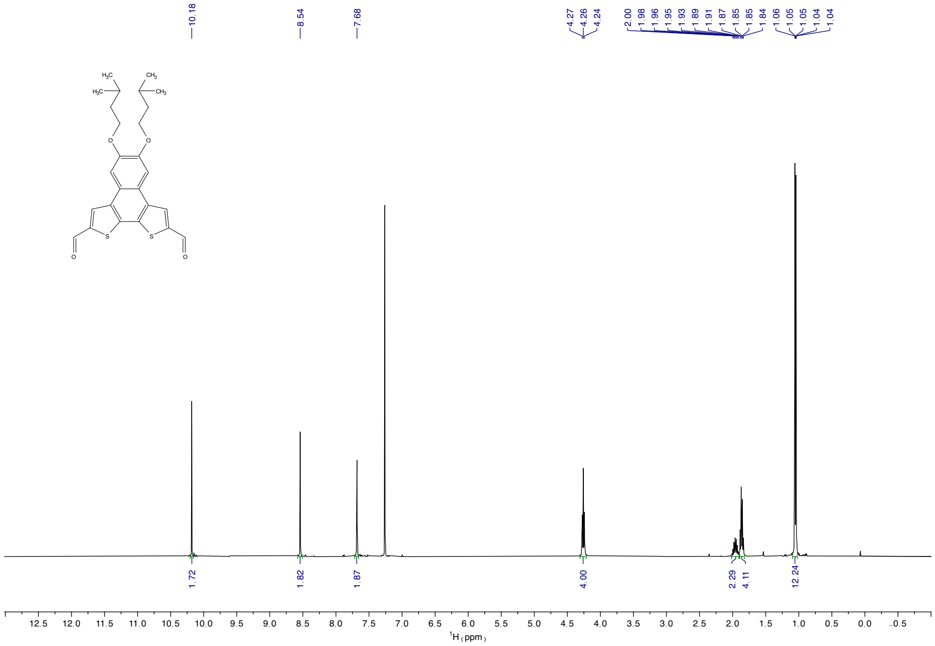

Figure S5. ^{1}H NMR spectrum of **α(CHO)$_2$** ($CDCl_3$, 400 MHz, 298 K).

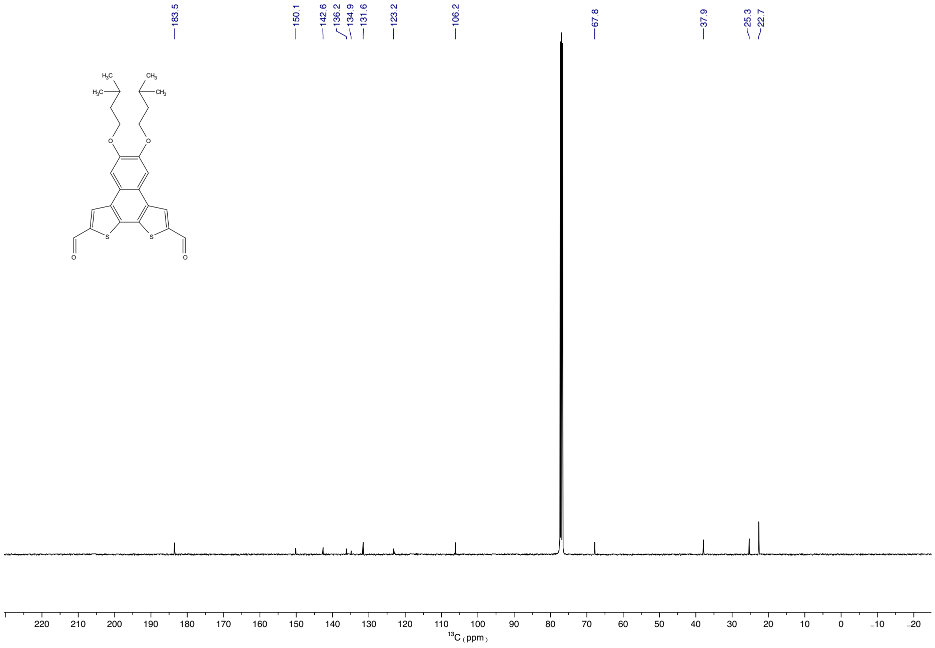

Figure S6. $^{13}C\{^1H\}$ NMR spectrum of **α(CHO)$_2$** ($CDCl_3$, 101 MHz, 298 K).

α(1)₂ Series

5,6-bis(isopentyloxy)-2,9-di-(*N*-(phenyl)methaniminyl)naphtho[2,1-*b*:3,4-*b'*]dithiophene, **α(1a)₂**

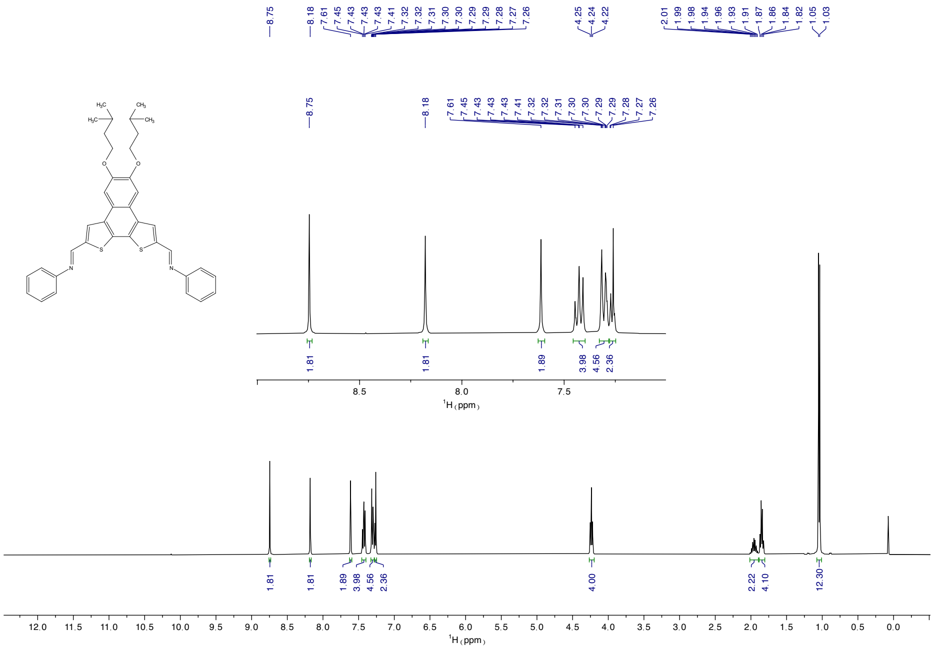

Figure S7. ¹H NMR spectrum of **α(1a)₂** ($CDCl_3$, 400 MHz, 298 K).

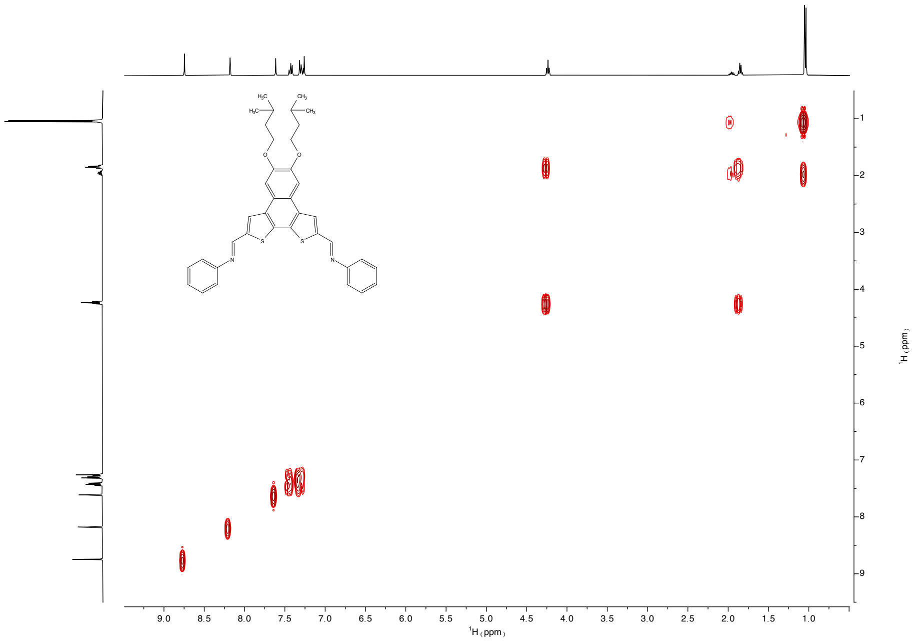

Figure S8. ¹H-¹H COSY spectrum of **α(1a)₂** ($CDCl_3$, 400 MHz, 298 K).

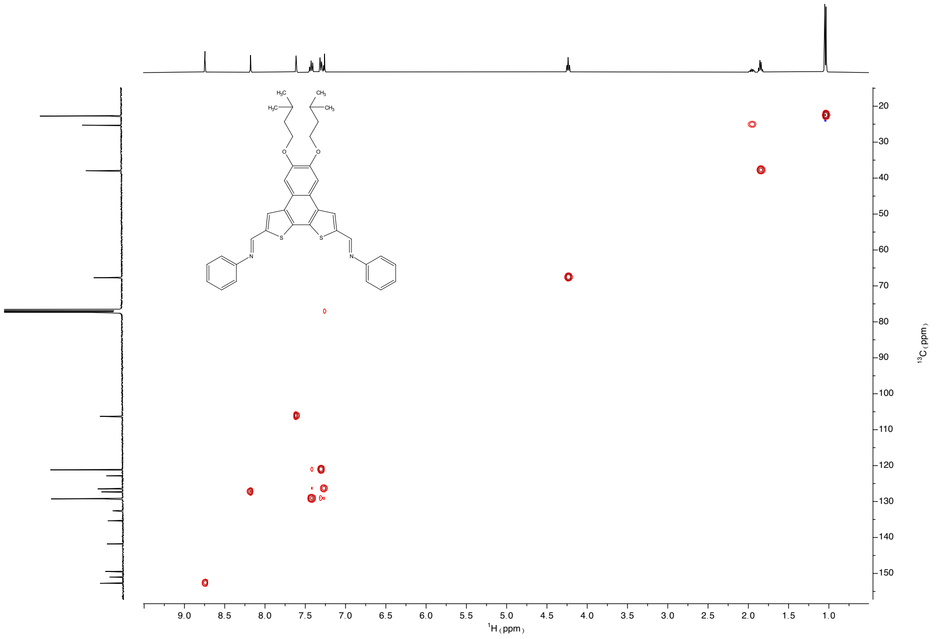

Figure S9. ^{1}H-^{13}C HSQC spectrum of **α(1a)₂** (CDCl$_3$, 400 MHz, 298 K).

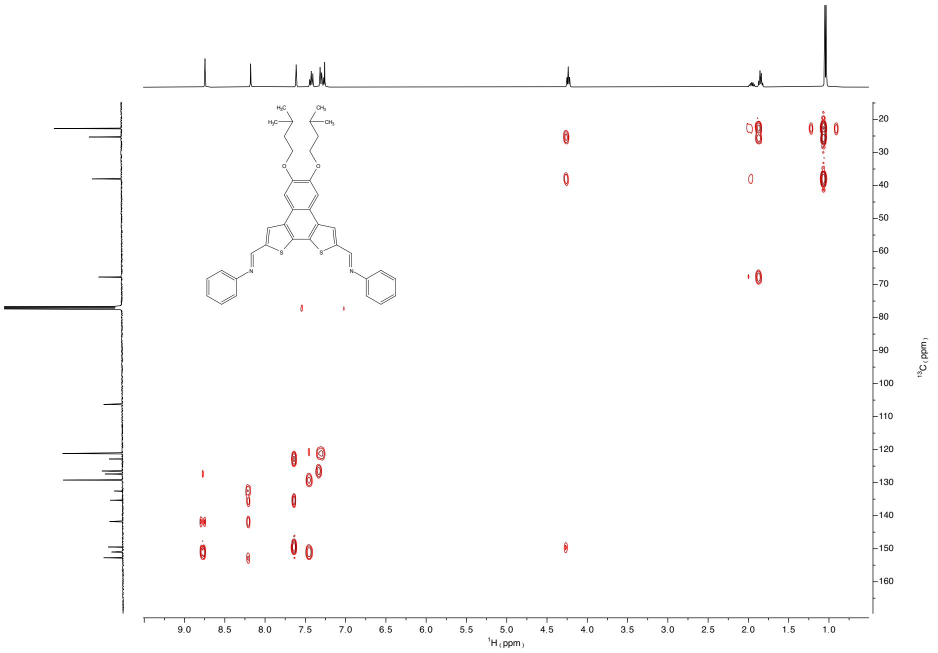

Figure S10. ^{1}H-^{13}C HMBC spectrum of **α(1a)₂** (CDCl$_3$, 400 MHz, 298 K).

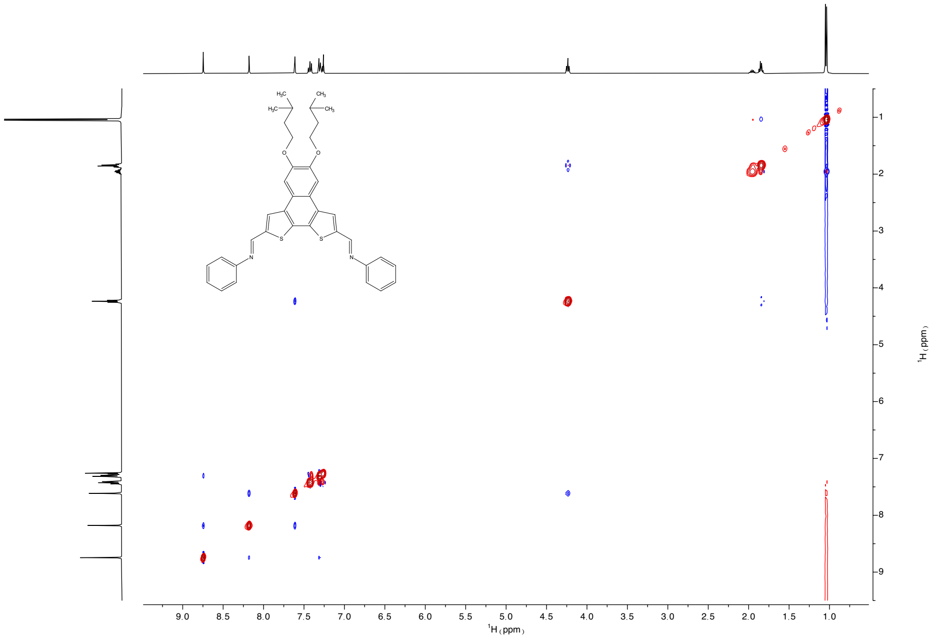

Figure S11. ^{1}H-^{1}H NOESY spectrum of **α(1a)$_2$** ($CDCl_3$, 400 MHz, 298 K).

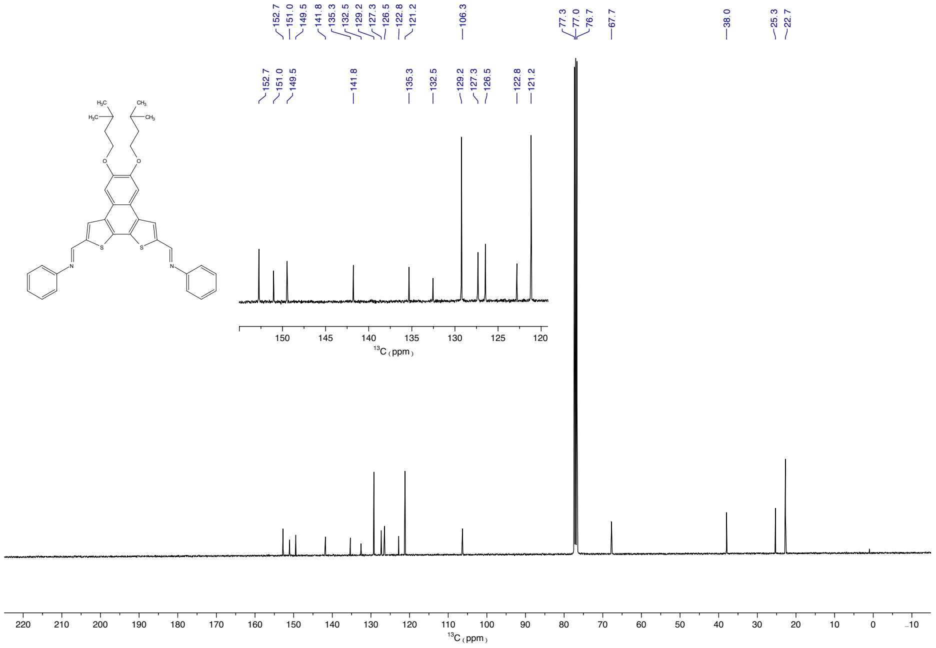

Figure S12. ^{13}C{^{1}H} NMR spectrum of **α(1a)$_2$** ($CDCl_3$, 101 MHz, 298 K).

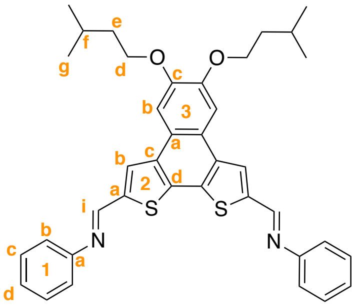

Assignment	^{1}H δ	^{13}C δ
1a	-	151.0
1b	7.28-7.33	121.2
1c	7.38-7.46	129.2
1d	7.22-7.28	126.5
i	8.75	152.7
2a	-	141.8
2b	8.18	127.3
2c	-	135.3
2d	-	132.5
3a	-	122.8
3b	7.61	106.3
3c	-	149.5
3d	4.24	67.7
3e	1.85	38.0
3f	1.96	25.3
3g	1.04	22.7

Figure S13. **α(1a)$_2$** with assignment of ^{1}H and ^{13}C resonances.

5,6-bis(isopentyloxy)-2,9-di-(*N*-(*p*-tolyl)methaniminyl)naphtho[2,1-*b*:3,4-*b'*]dithiophene, **α(1b)₂**

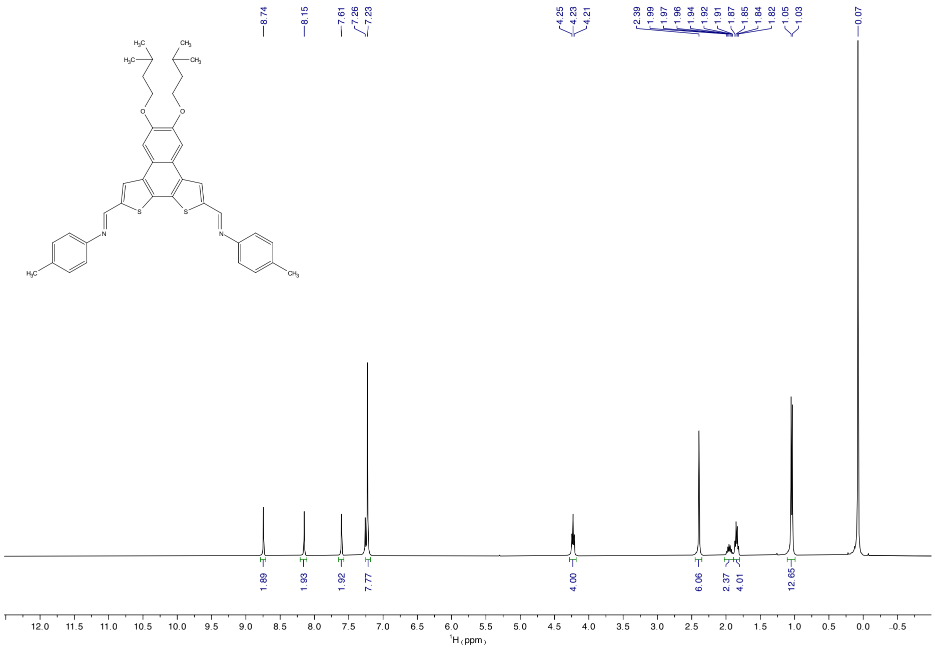

Figure S14. ^{1}H NMR spectrum of **α(1b)$_2$** ($CDCl_3$, 400 MHz, 298 K).

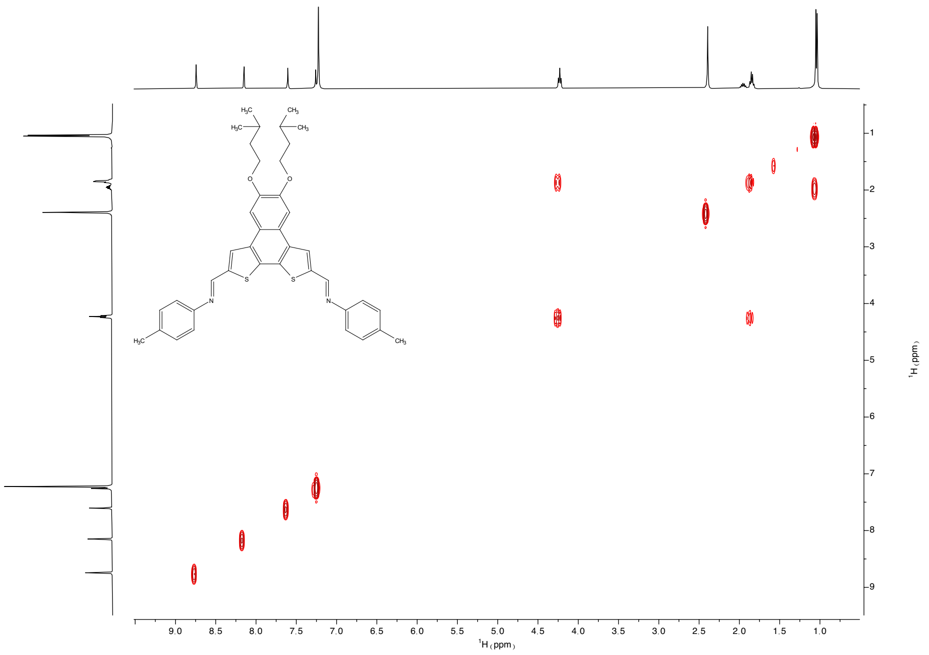

Figure S15. ^{1}H-^{1}H COSY spectrum of **α(1b)$_2$** ($CDCl_3$, 400 MHz, 298 K).

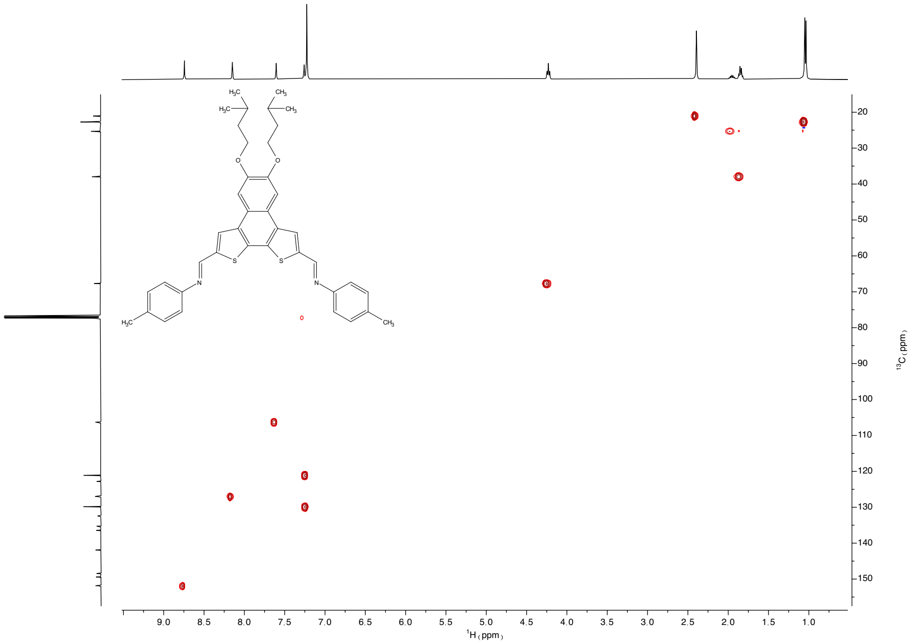

Figure S16. ^{1}H-^{13}C HSQC spectrum of **α(1b)₂** (CDCl₃, 400 MHz, 298 K).

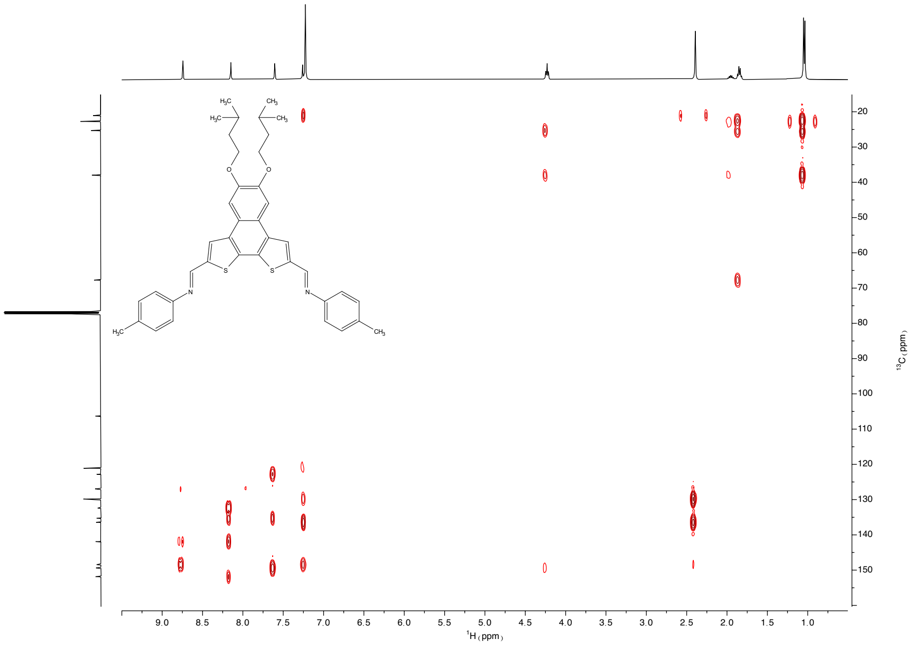

Figure S17. ^{1}H-^{13}C HMBC spectrum of **α(1b)₂** (CDCl₃, 400 MHz, 298 K).

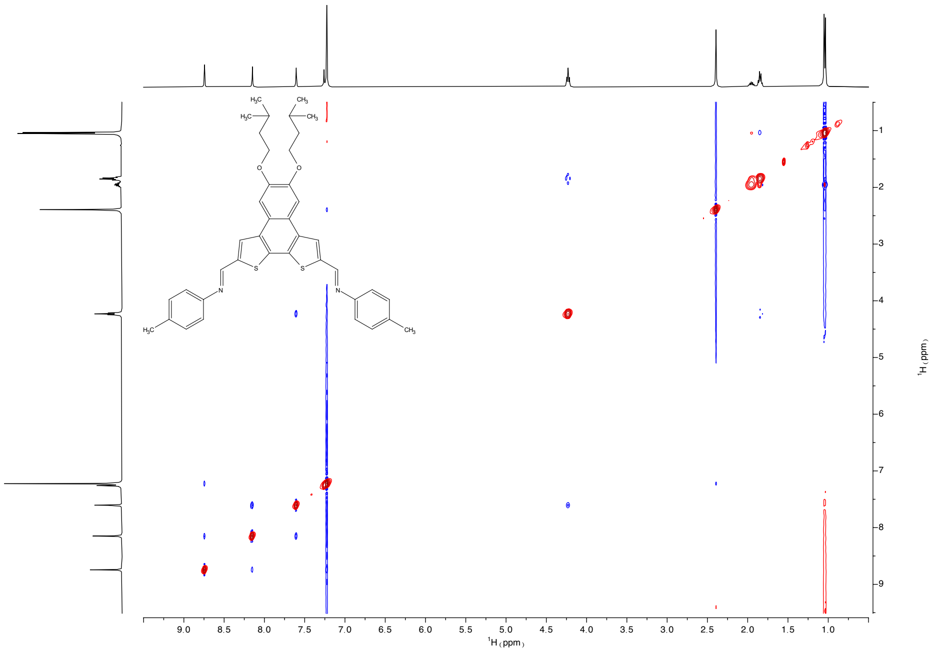

Figure S18. ^{1}H-^{1}H NOESY spectrum of **α(1b)₂** (CDCl₃, 400 MHz, 298 K).

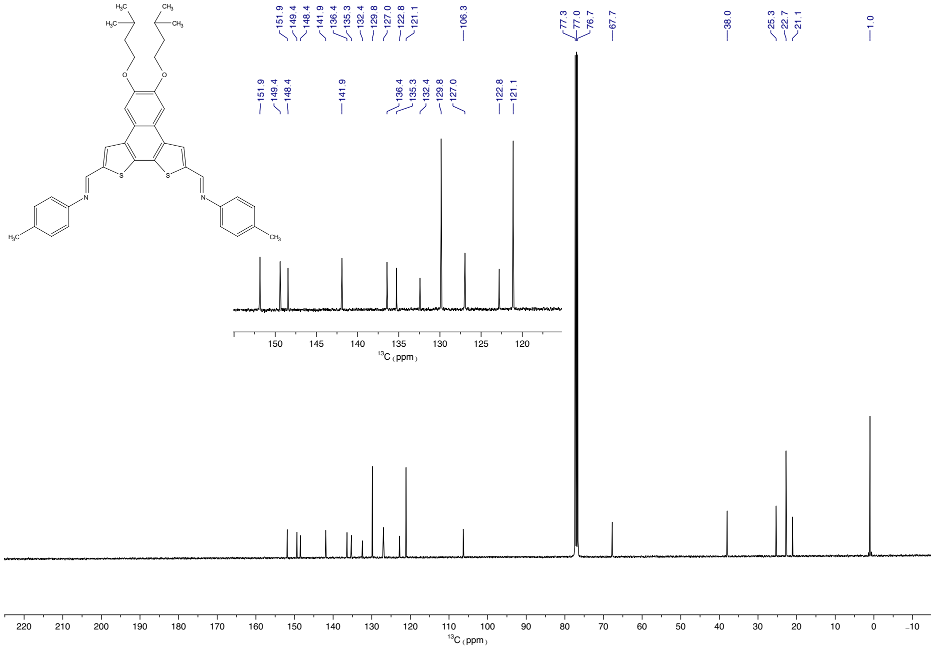

Figure S19. $^{13}C\{^{1}H\}$ NMR spectrum of **α(1b)₂** (CDCl₃, 101 MHz, 298 K).

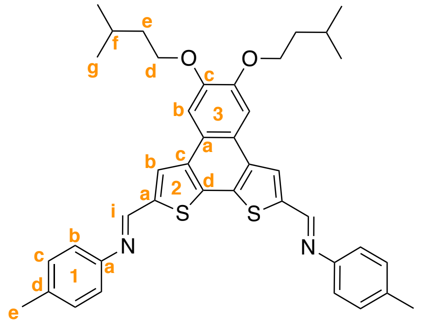

Assignment	$^{1}H\ \delta$	$^{13}C\ \delta$
1a	-	148.4
1b	7.23	121.1
1c	7.23	129.8
1d	-	136.4
1e	2.39	21.1
i	8.74	151.9
2a	-	141.9
2b	8.15	127.0
2c	-	135.2
2d	-	132.4
3a	-	122.8
3b	7.61	106.3
3c	-	149.4
3d	4.23	67.7
3e	1.84	37.9
3f	1.96	25.3
3g	1.04	22.7

Figure S20. **α(1b)$_2$** with assignment of ^{1}H and ^{13}C resonances.

5,6-bis(isopentyloxy)-2,9-di-(*N*-(*p*-methoxyphenyl)methaniminyl)naphtho[2,1-*b*:3,4-*b'*]dithiophene, **α(1c)₂**

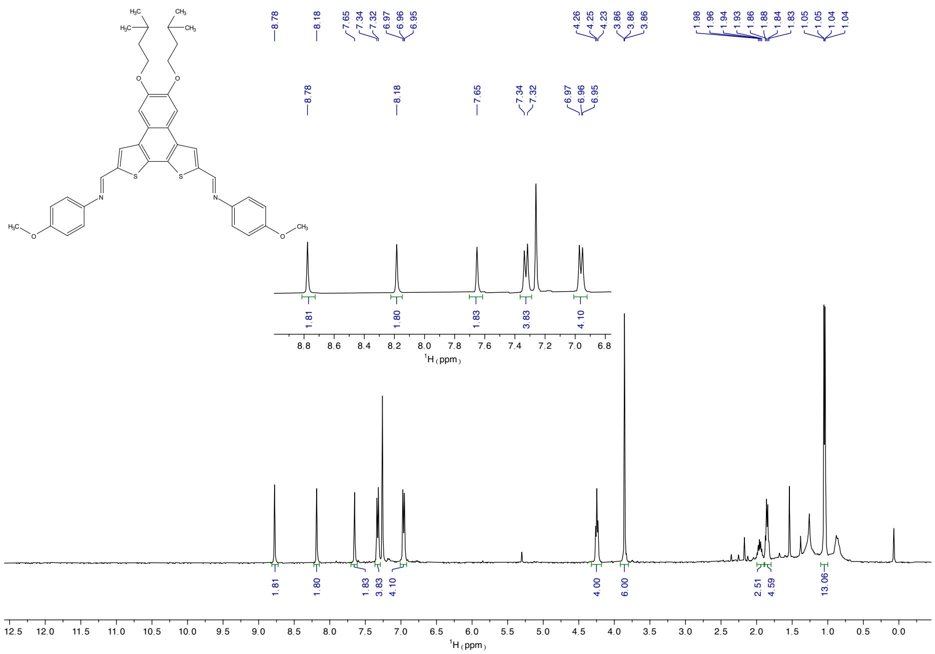

Figure S21. ^{1}H NMR spectrum of **α(1c)₂** ($CDCl_3$, 400 MHz, 298 K).

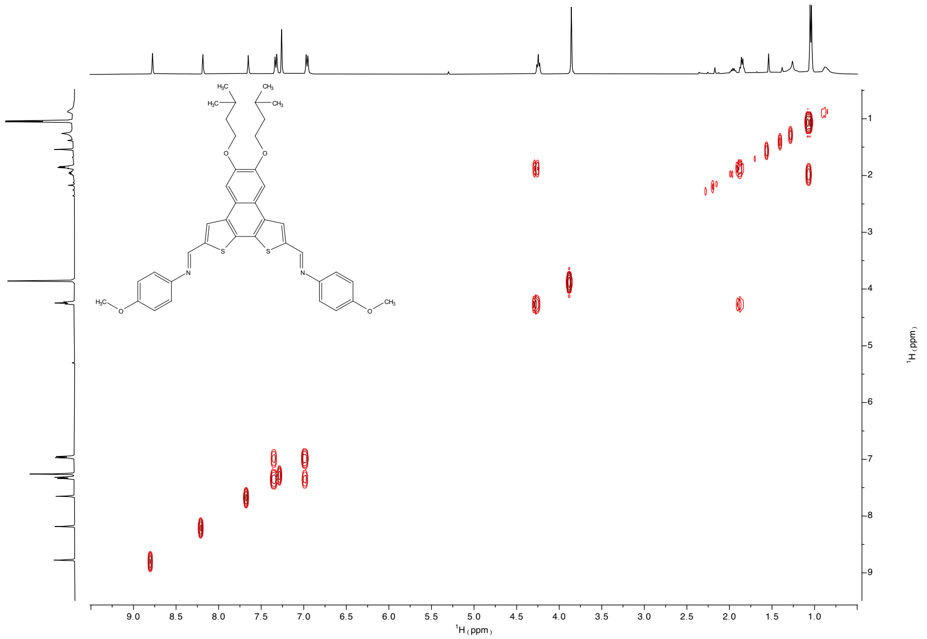

Figure S22. ^{1}H-^{1}H COSY spectrum of **α(1c)₂** ($CDCl_3$, 400 MHz, 298 K).

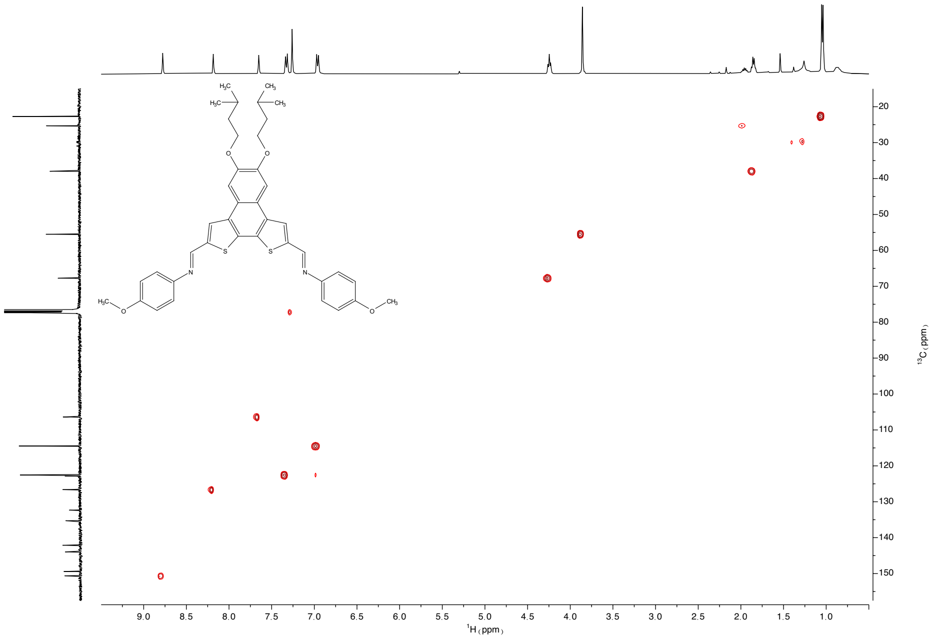

Figure S23. ^{1}H-^{13}C HSQC spectrum of **α(1c)₂** (CDCl$_3$, 400 MHz, 298 K).

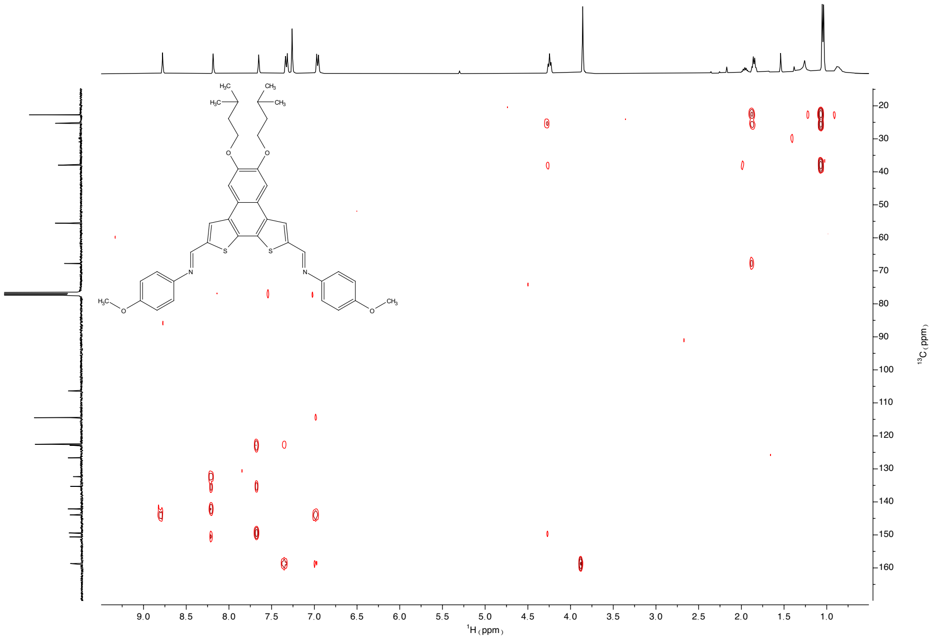

Figure S24. ^{1}H-^{13}C HMBC spectrum of **α(1c)₂** (CDCl$_3$, 400 MHz, 298 K).

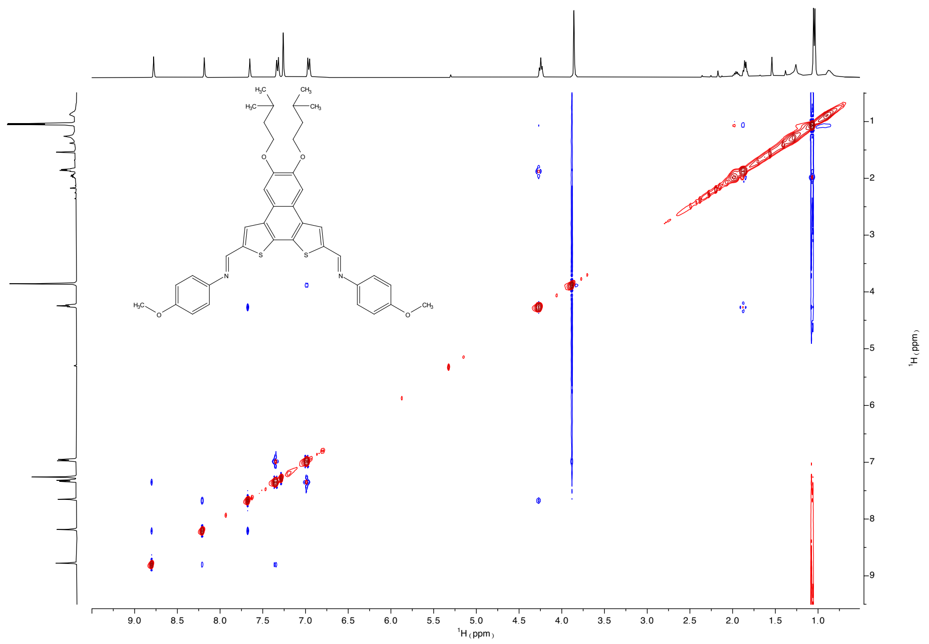

Figure S25. ^{1}H-^{1}H NOESY spectrum of **α(1c)₂** (CDCl$_3$, 400 MHz, 298 K).

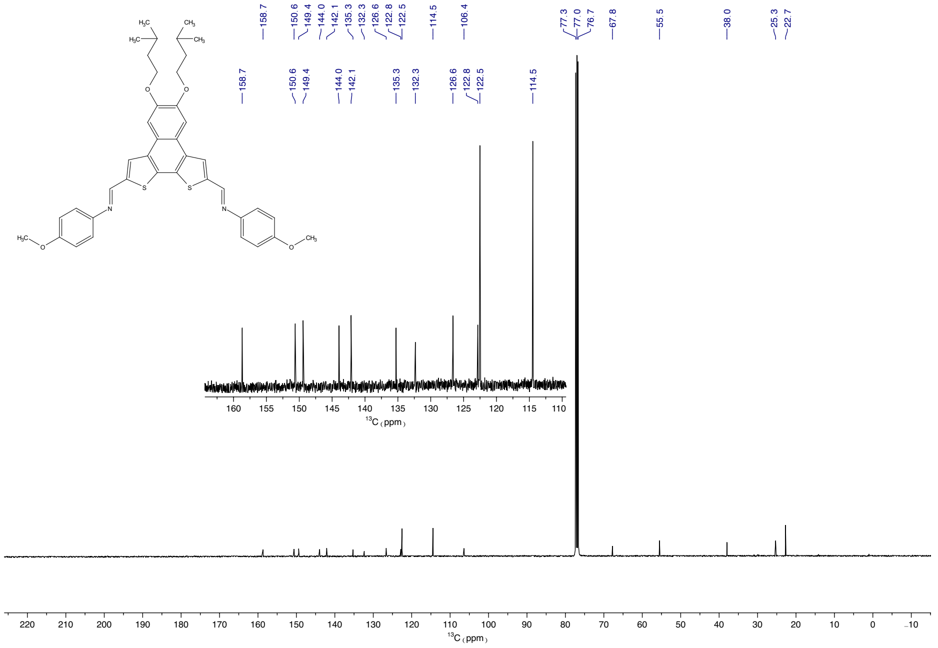

Figure S26. ^{13}C{^{1}H} NMR spectrum of **α(1c)₂** (CDCl$_3$, 101 MHz, 298 K).

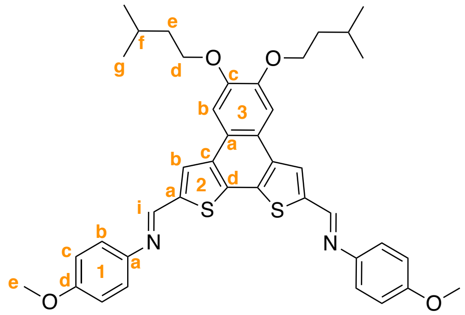

Assignment	^{1}H δ	^{13}C δ
1a	-	158.7
1b	7.33	122.5
1c	6.96	114.4
1d	-	158.7
1e	3.86	55.5
i	8.78	150.6
2a	-	142.1
2b	8.18	126.6
2c	-	135.3
2d	-	132.3
3a	-	122.8
3b	7.65	106.4
3c	-	149.4
3d	4.25	67.8
3e	1.85	38.0
3f	2.03-1.92	25.3
3g	1.04	22.7

Figure S27. **α(1c)$_2$** with assignment of ^{1}H and ^{13}C resonances.

5,6-bis(isopentyloxy)-2,9-di-(*N*-(*p*-trifluoromethylphenyl)methaniminyl)naphtho[2,1-*b*:3,4-*b'*]dithiophene, **α(1d)₂**

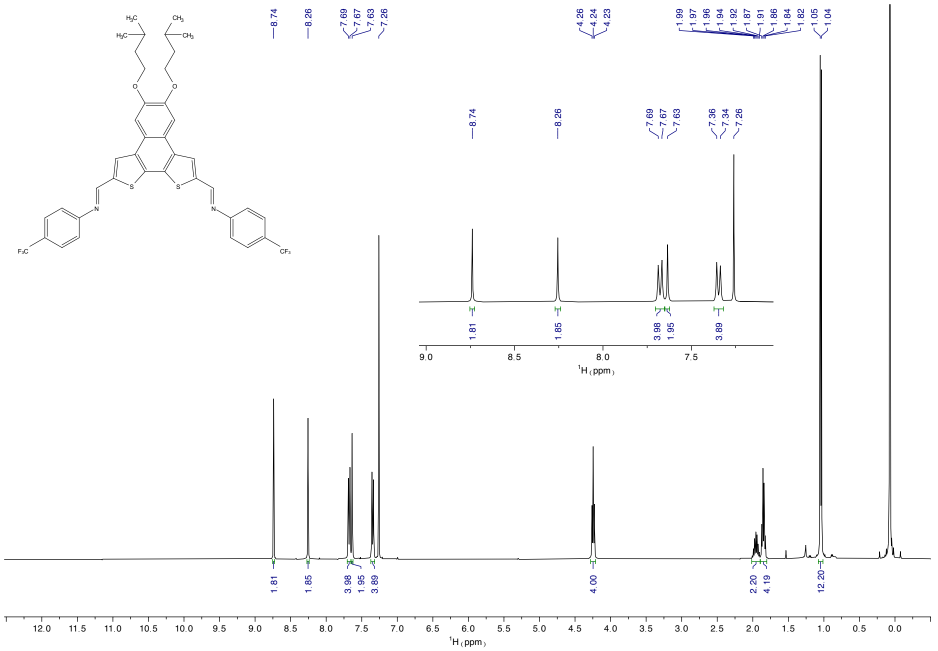

Figure S28. ¹H NMR spectrum of **α(1d)₂** ($CDCl_3$, 400 MHz, 298 K).

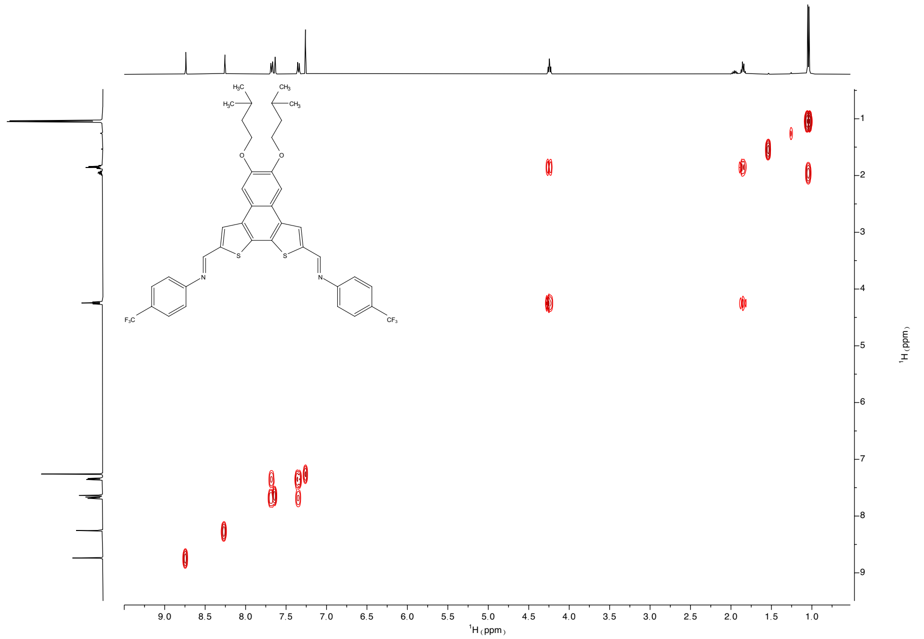

Figure S29. ¹H-¹H COSY spectrum of **α(1d)₂** ($CDCl_3$, 400 MHz, 298 K).

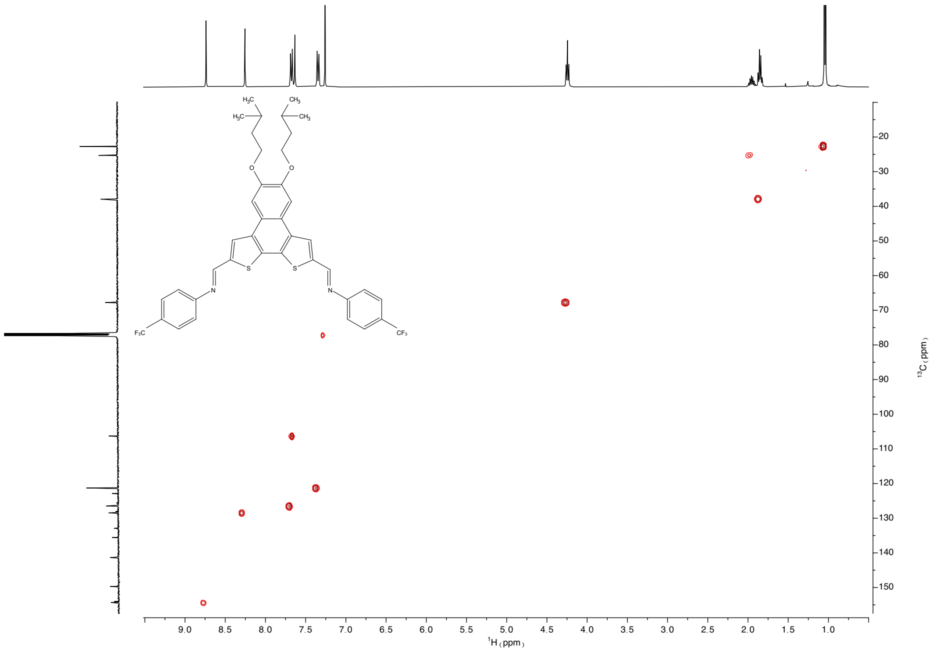

Figure S30. ^{1}H-^{13}C HSQC spectrum of **α(1d)₂** (CDCl₃, 400 MHz, 298 K).

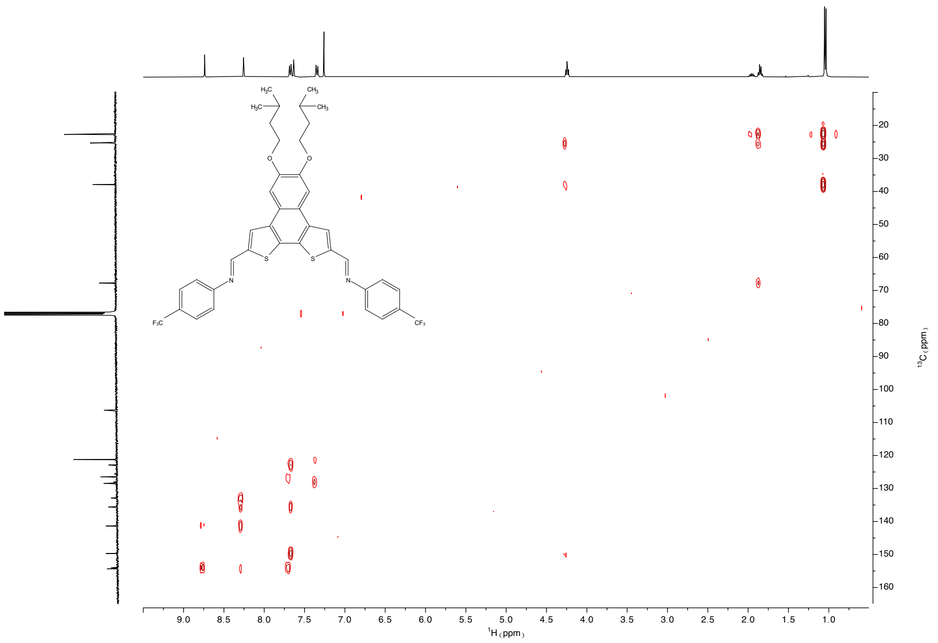

Figure S31. ^{1}H-^{13}C HMBC spectrum of **α(1d)₂** (CDCl₃, 400 MHz, 298 K).

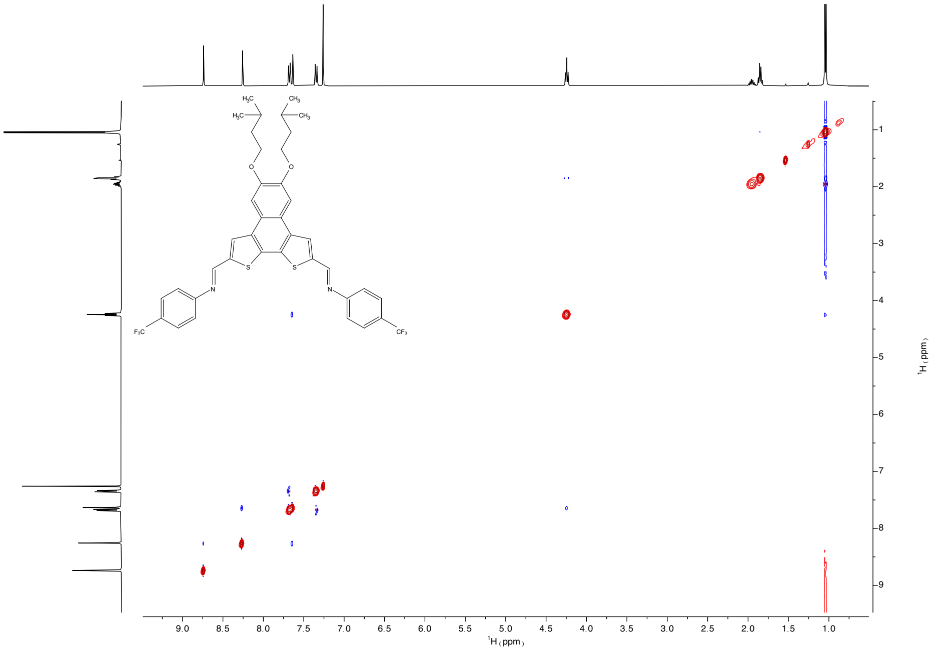

Figure S32. ^{1}H-^{1}H NOESY spectrum of **α(1d)₂** (CDCl$_3$, 400 MHz, 298 K).

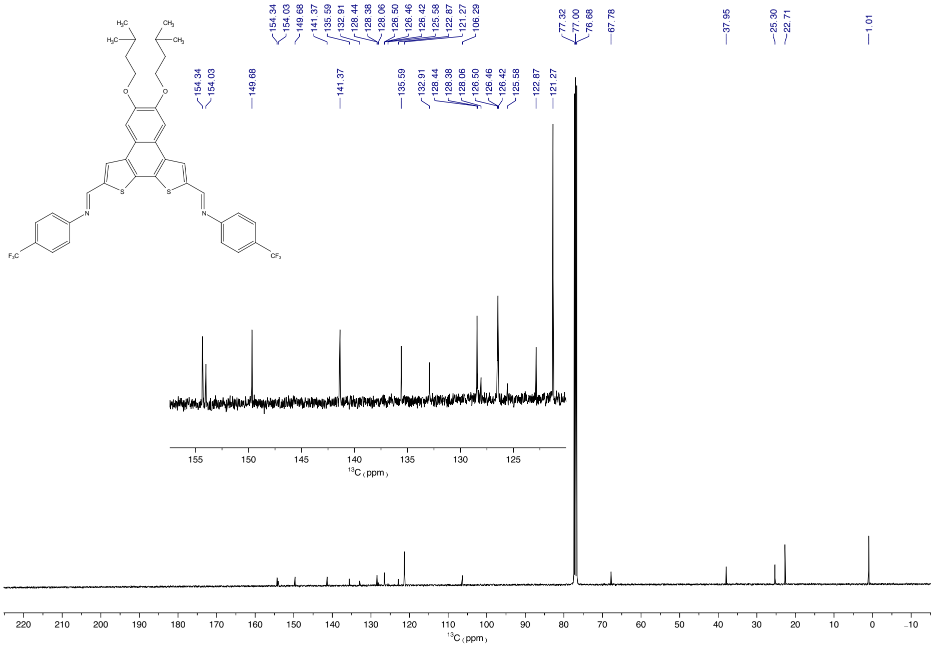

Figure S33. ^{13}C{^{1}H} NMR spectrum of **α(1d)₂** (CDCl$_3$, 101 MHz, 298 K).

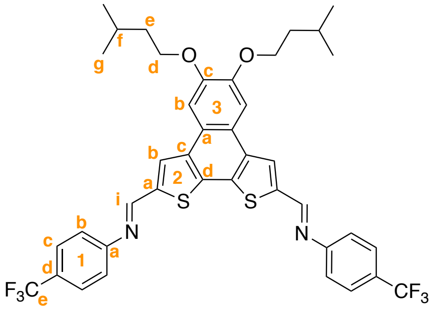

Assignment	^{1}H δ	^{13}C δ
1a	-	154.0
1b	7.68	125.6
1c	7.35	121.3
1d	-	128.1 (d, J=32 Hz)
1e	-	124.2 (J = 273 Hz)*
i	8.74	154.3
2a	-	141.4
2b	8.26	128.4
2c	-	135.6
2d	-	132.9
3a	-	122.9
3b	7.63	106.3
3c	-	149.7
3d	4.24	67.8
3e	1.85	37.9
3f	1.95	25.3
3g	1.04	22.7

Figure S34. **α(1d)₂** with assignment of ^{1}H and ^{13}C resonances. *Additional quartet signals are weak and overlapped with more prominent signals; with reduced line broadening an additional signal at ~122.9 is visible and provides ~273 Hz coupling constant.

5,6-bis(isopentyloxy)-2,9-di-(*N*-(3,5-dimethylphenyl)methaniminyl)naphtho[2,1-*b*:3,4-*b'*]dithiophene, **α(1bb)₂**

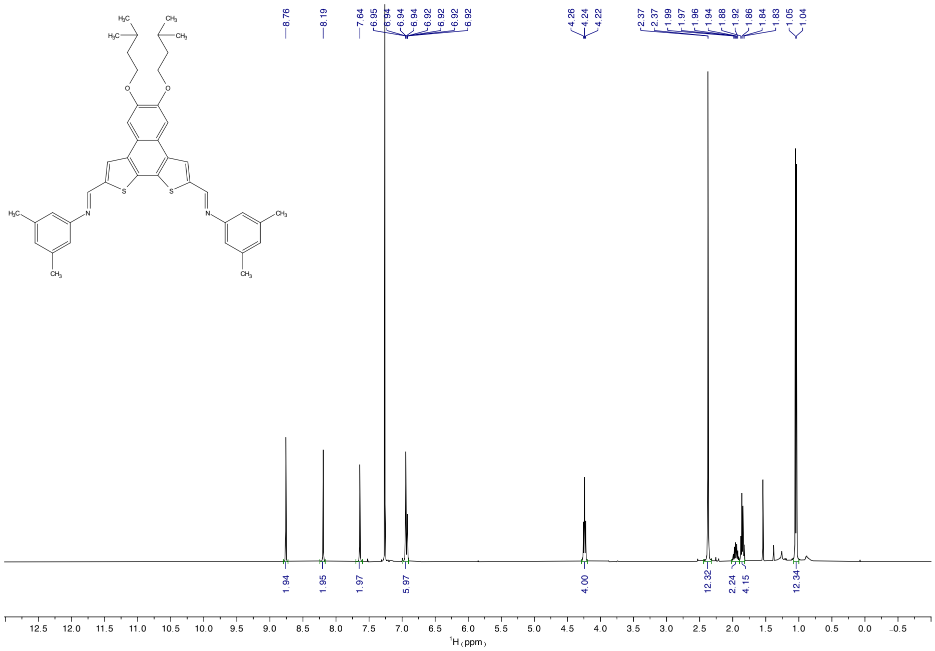

Figure S35. ^{1}H NMR spectrum of **α(1bb)₂** ($CDCl_3$, 400 MHz, 298 K).

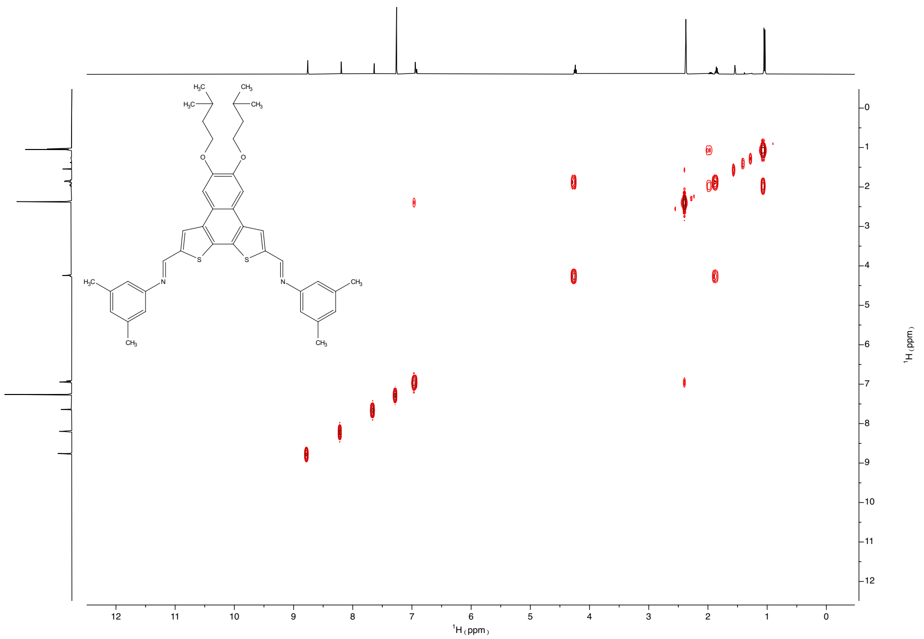

Figure S36. ^{1}H-^{1}H COSY spectrum of **α(1bb)₂** ($CDCl_3$, 400 MHz, 298 K).

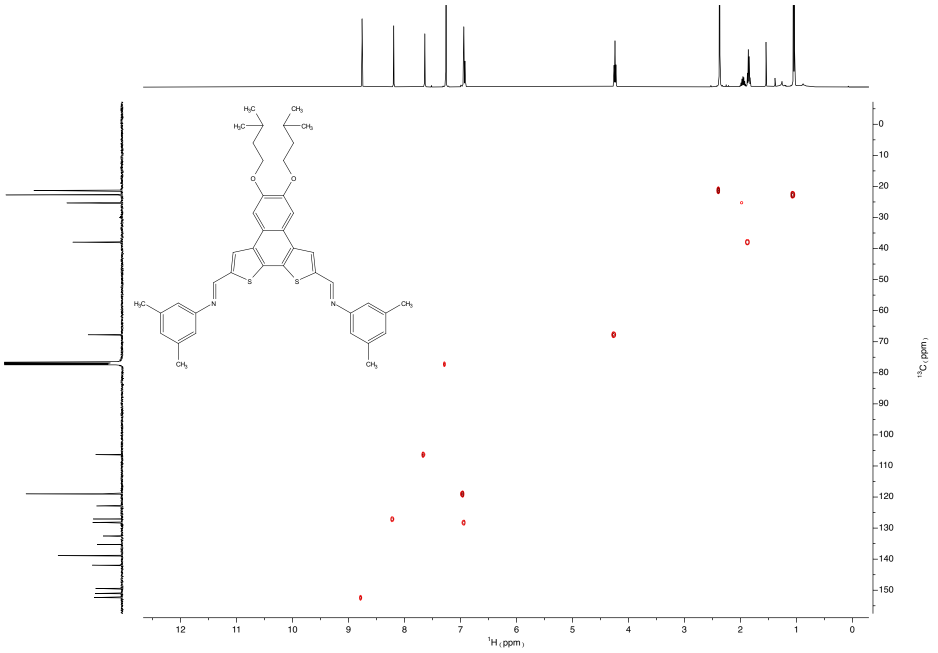

Figure S37. ^{1}H-^{13}C HSQC spectrum of **α(1bb)$_2$** ($CDCl_3$, 400 MHz, 298 K).

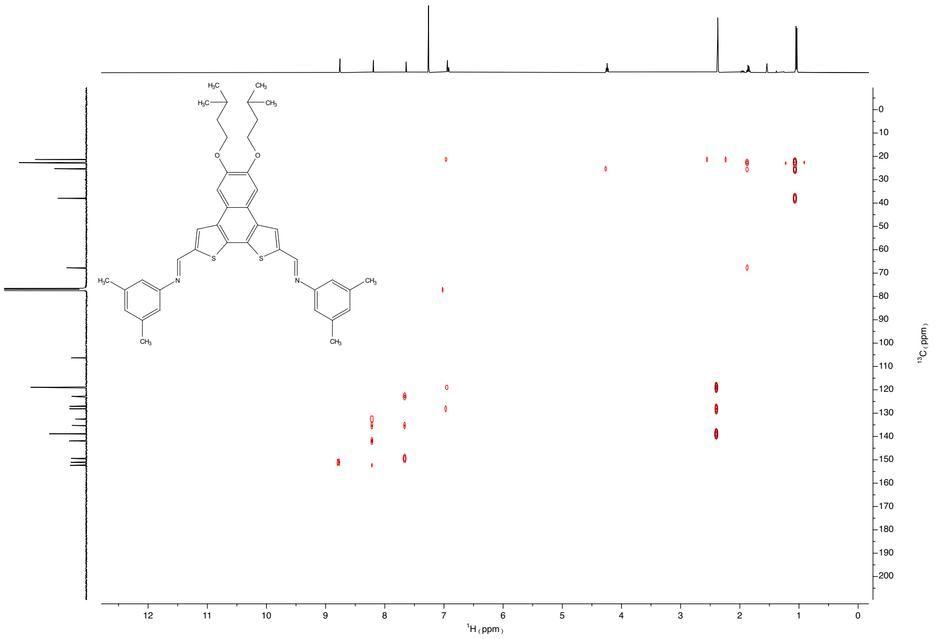

Figure S38. ^{1}H-^{13}C HMBC spectrum of **α(1bb)$_2$** ($CDCl_3$, 400 MHz, 298 K).

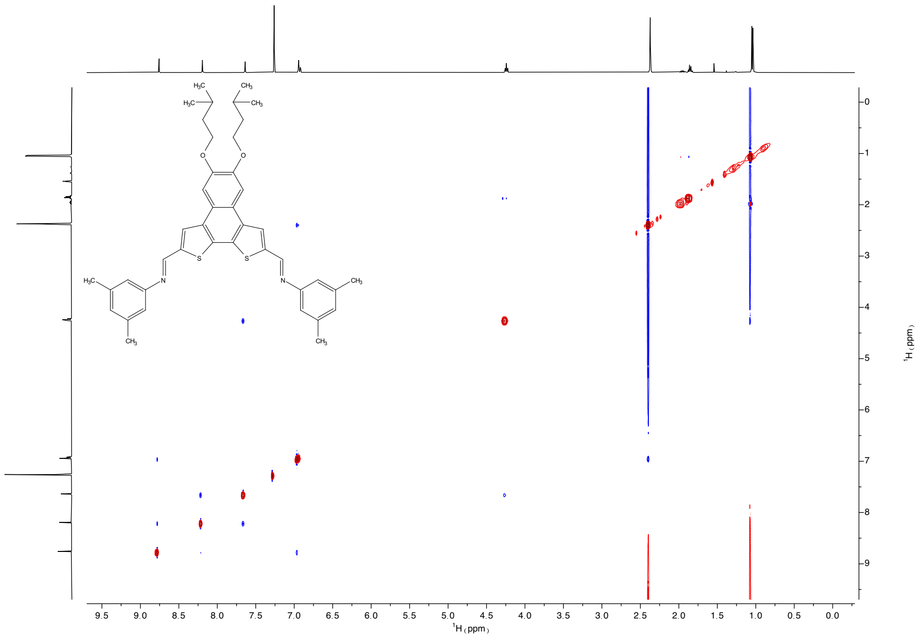

Figure S39. ^{1}H-^{1}H NOESY spectrum of **α(1bb)₂** (CDCl$_3$, 400 MHz, 298 K).

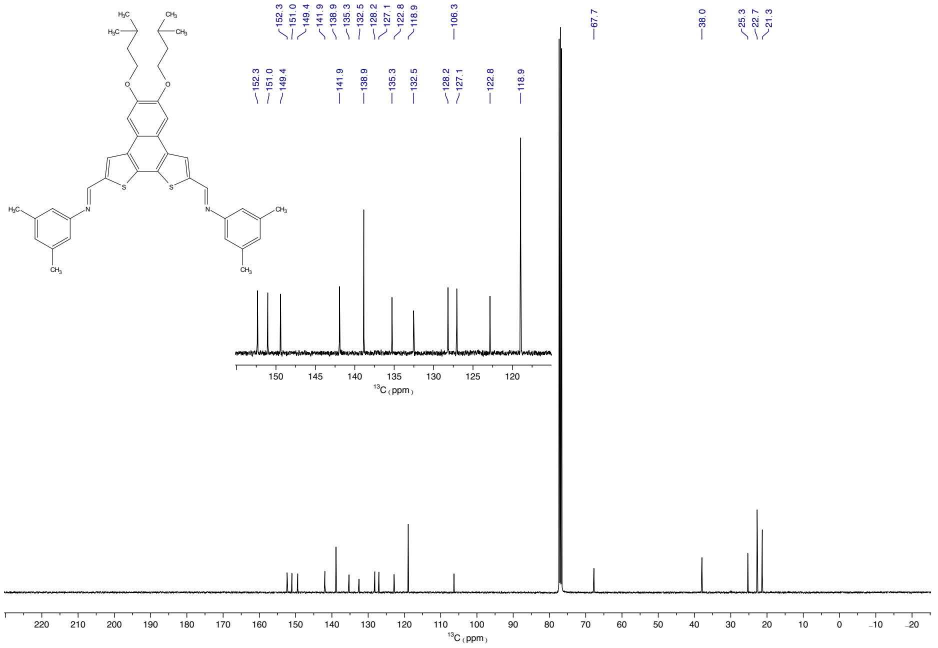

Figure S40. ^{13}C{^{1}H} NMR spectrum of **α(1bb)₂** (CDCl$_3$, 101 MHz, 298 K).

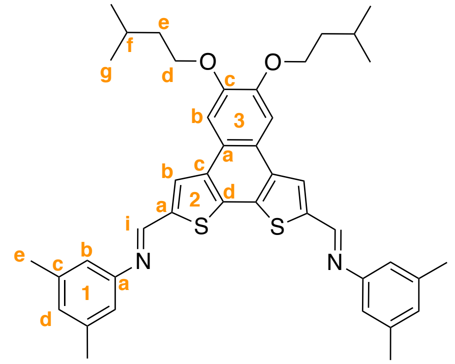

Assignment	$^{1}H\ \delta$	$^{13}C\ \delta$
1a	-	151.0
1b	6.94	118.9
1c	-	138.9
1d	6.92	128.2
1e	2.37	21.3
i	8.76	152.3
2a	-	141.9
2b	8.19	127.1
2c	-	135.3
2d	-	132.5
3a	-	122.8
3b	7.64	106.4
3c	-	149.4
3d	4.24	67.7
3e	1.85	38.0
3f	1.96	25.3
3g	1.04	22.7

Figure S41. **α(1bb)$_2$** with assignment of ^{1}H and ^{13}C resonances

5,6-bis(isopentyloxy)-2,9-di-(*N*-(3,5-dimethoxyphenyl)methaniminyl)naphtho[2,1-*b*:3,4-*b'*]dithiophene, **α(1cc)₂**

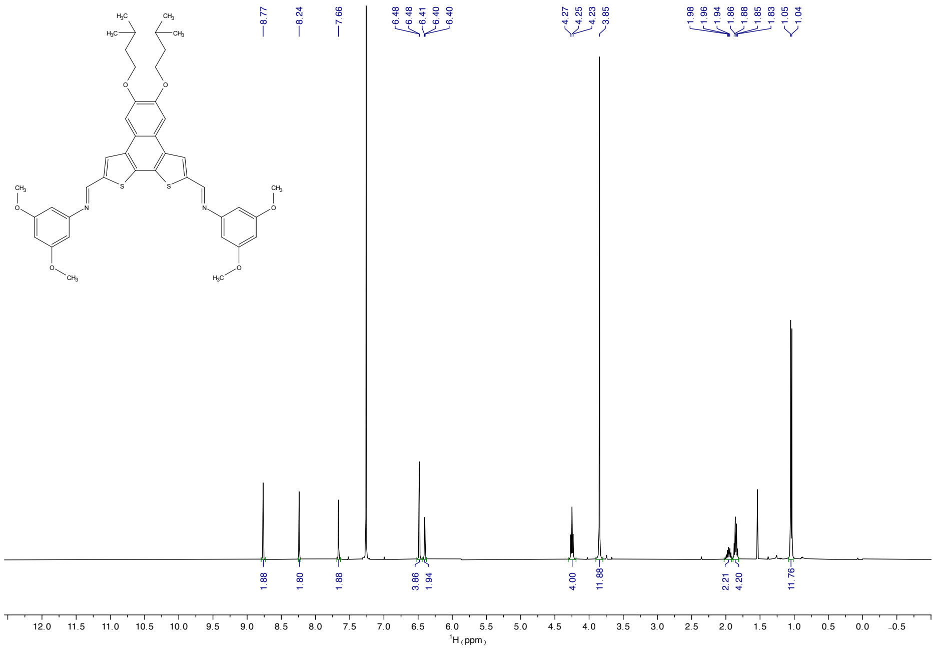

Figure S42. ^{1}H NMR spectrum of **α(1cc)₂** ($CDCl_3$, 400 MHz, 298 K).

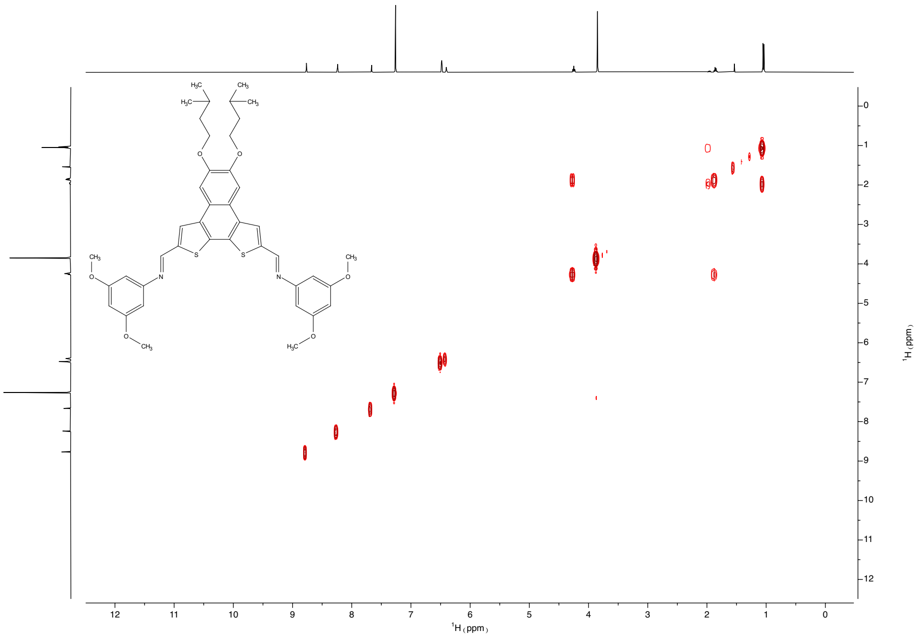

Figure S43. ^{1}H-^{1}H COSY spectrum of **α(1cc)₂** ($CDCl_3$, 400 MHz, 298 K).

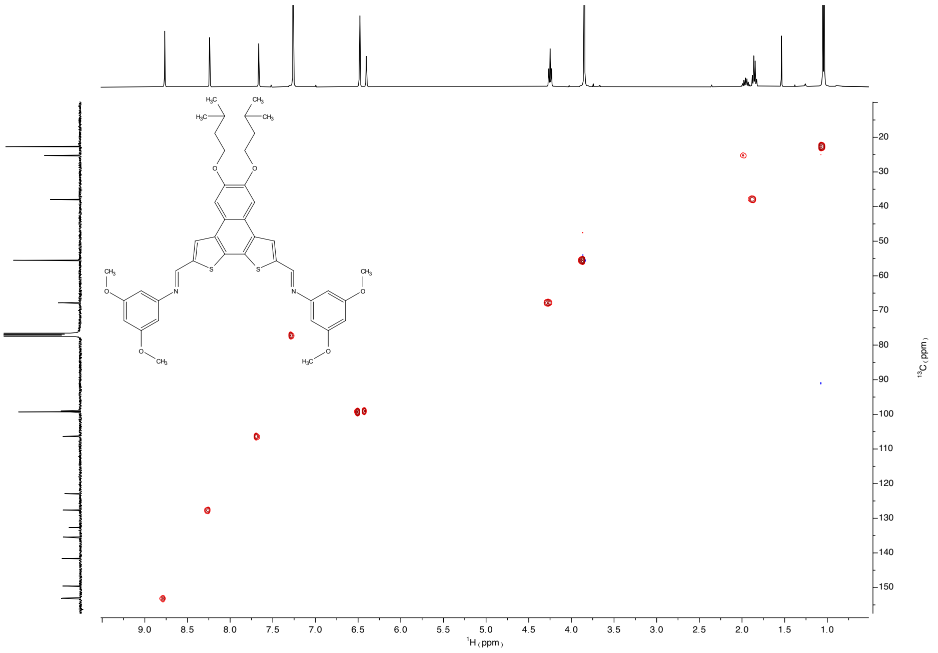

Figure S44. ^{1}H-^{13}C HSQC spectrum of **α(1cc)₂** ($CDCl_3$, 400 MHz, 298 K).

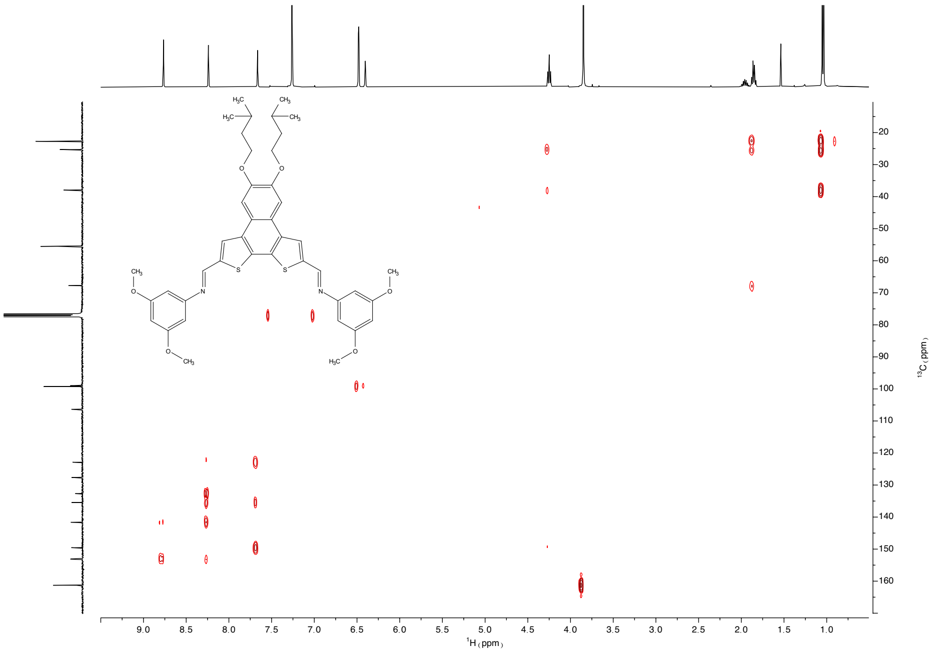

Figure S45. ^{1}H-^{13}C HMBC spectrum of **α(1cc)₂** ($CDCl_3$, 400 MHz, 298 K).

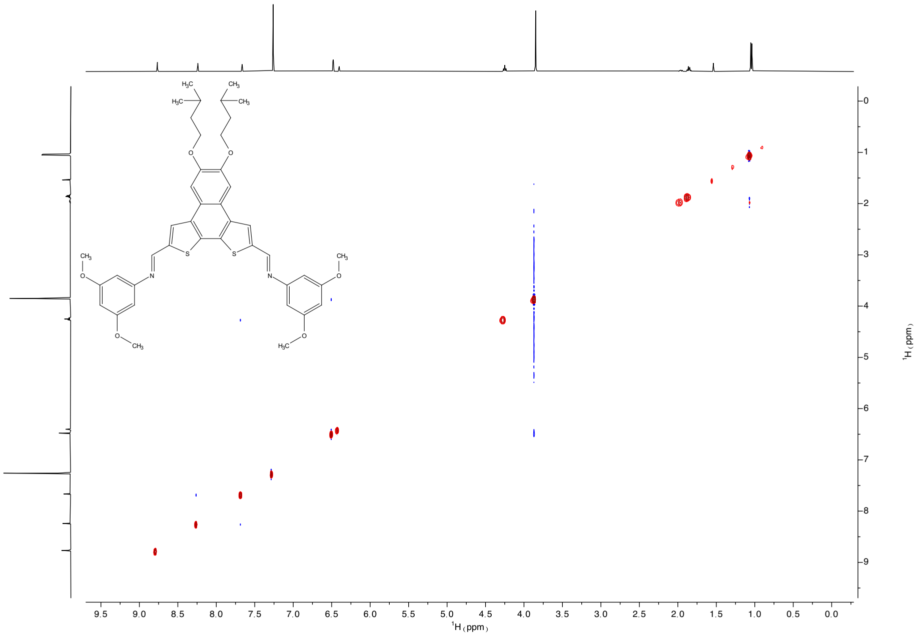

Figure S46. ^{1}H-^{1}H NOESY spectrum of **α(1cc)₂** (CDCl$_3$, 400 MHz, 298 K).

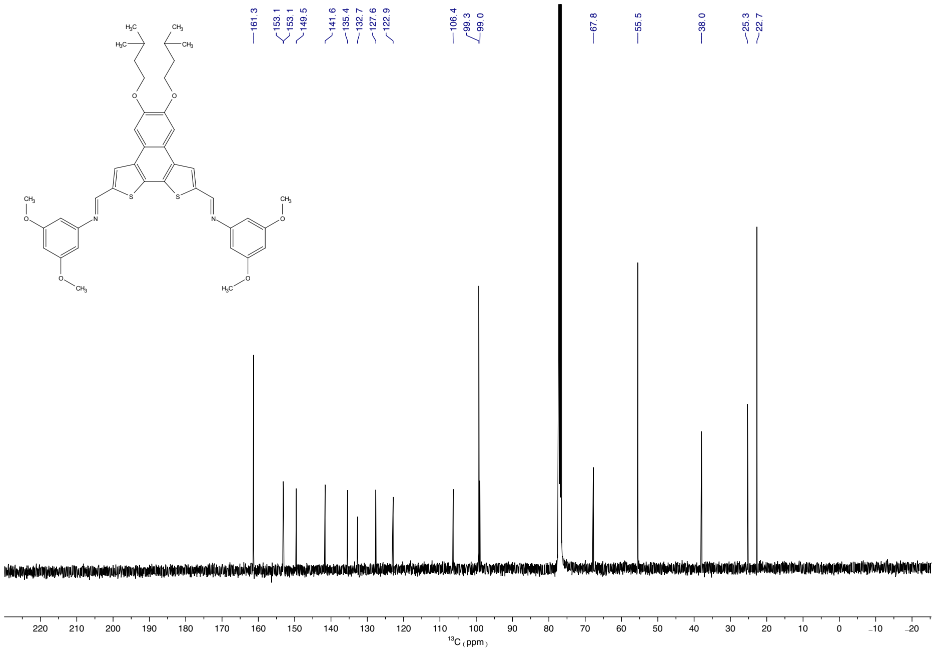

Figure S47. ^{13}C{^{1}H} NMR spectrum of **α(1cc)₂** (CDCl$_3$, 101 MHz, 298 K).

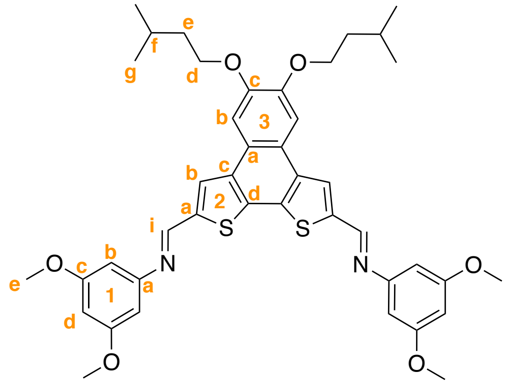

Assignment	^{1}H δ	^{13}C δ
1a	-	153.1*
1b	6.48	99.3
1c	-	161.3
1d	3.85	99.0
1e	6.40	55.5
i	8.77	153.1*
2a	-	141.6
2b	8.24	127.6
2c	-	135.4
2d	-	132.7
3a	-	122.9
3b	7.66	106.4
3c	-	149.5
3d	4.25	67.8
3e	1.85	38.0
3f	1.95	25.3
3g	1.04	22.7

Figure S48. **α(1cc)₂** with assignment of ^{1}H and ^{13}C resonances. *Signals are overlapped and resolved by < 0.1 ppm.

5,6-bis(isopentyloxy)-2,9-di-(*N*-(3,5-ditrifluoromethylphenyl)methaniminyl)naphtho[2,1-*b*:3,4-*b'*]dithiophene, **α(1dd)₂**

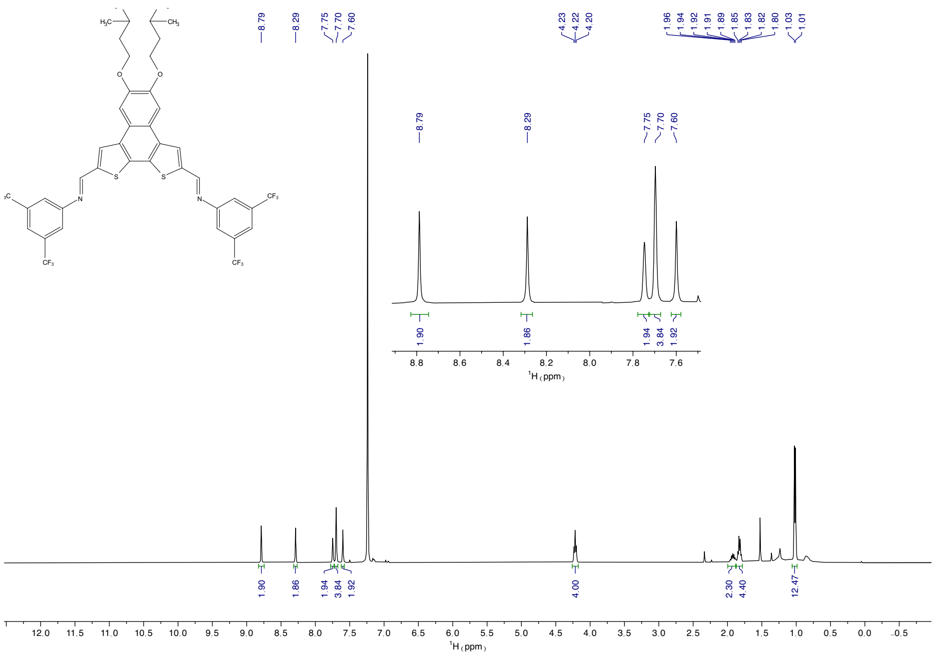

Figure S49. ^{1}H NMR spectrum of **α(1dd)₂** ($CDCl_3$, 400 MHz, 298 K).

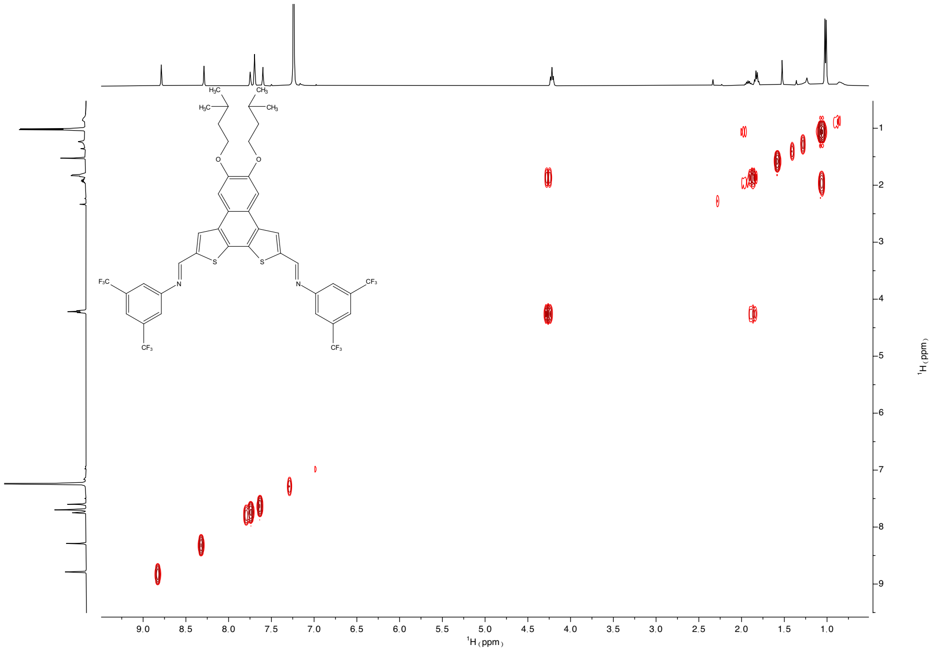

Figure S50. ^{1}H-^{1}H COSY spectrum of **α(1dd)₂** ($CDCl_3$, 400 MHz, 298 K).

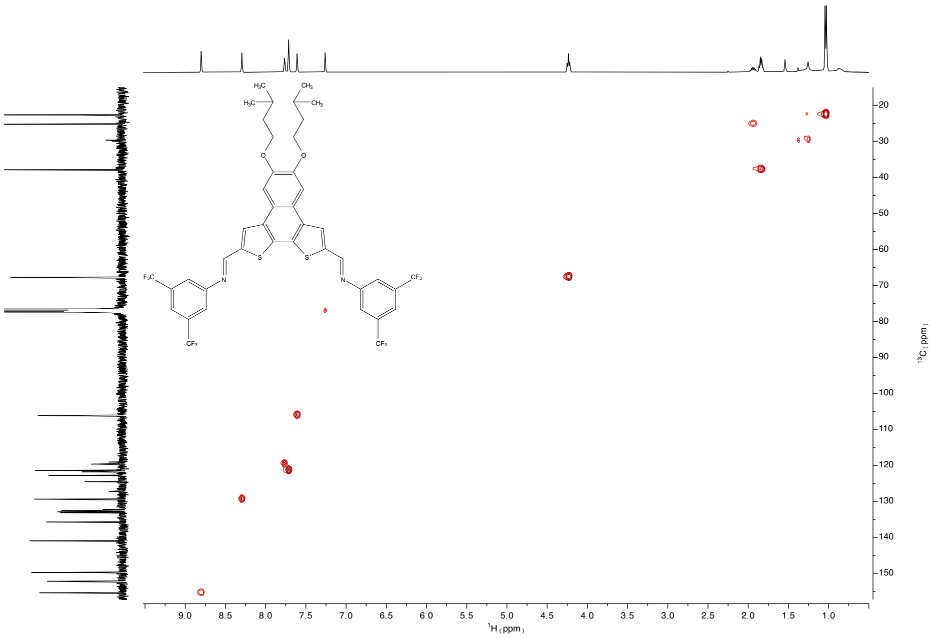

Figure S51. ^{1}H-^{13}C HSQC spectrum of **α(1dd)₂** (CDCl$_3$, 400 MHz, 298 K).

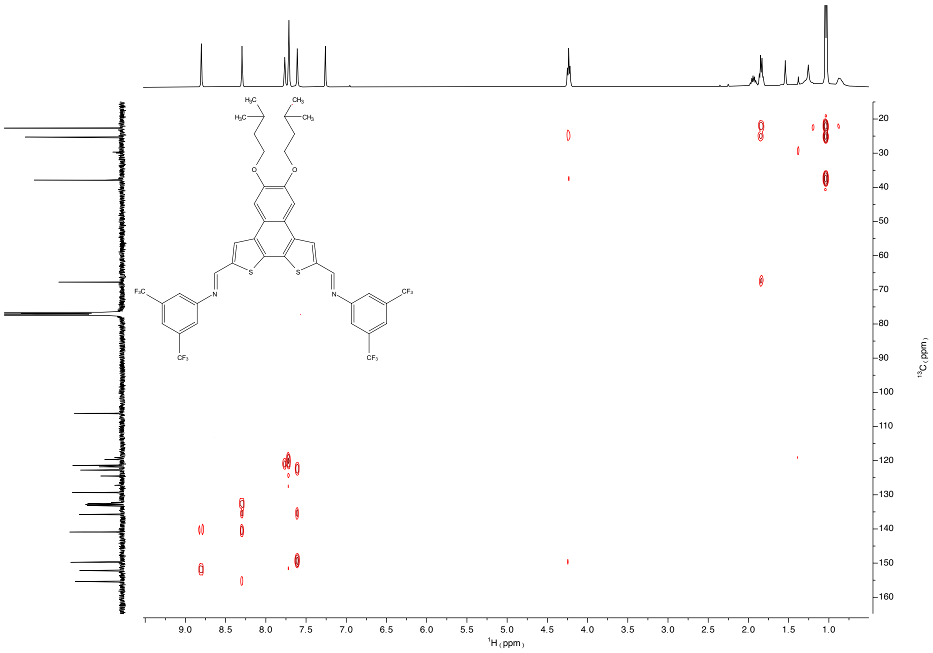

Figure S52. ^{1}H-^{13}C HMBC spectrum of **α(1dd)₂** (CDCl$_3$, 400 MHz, 298 K).

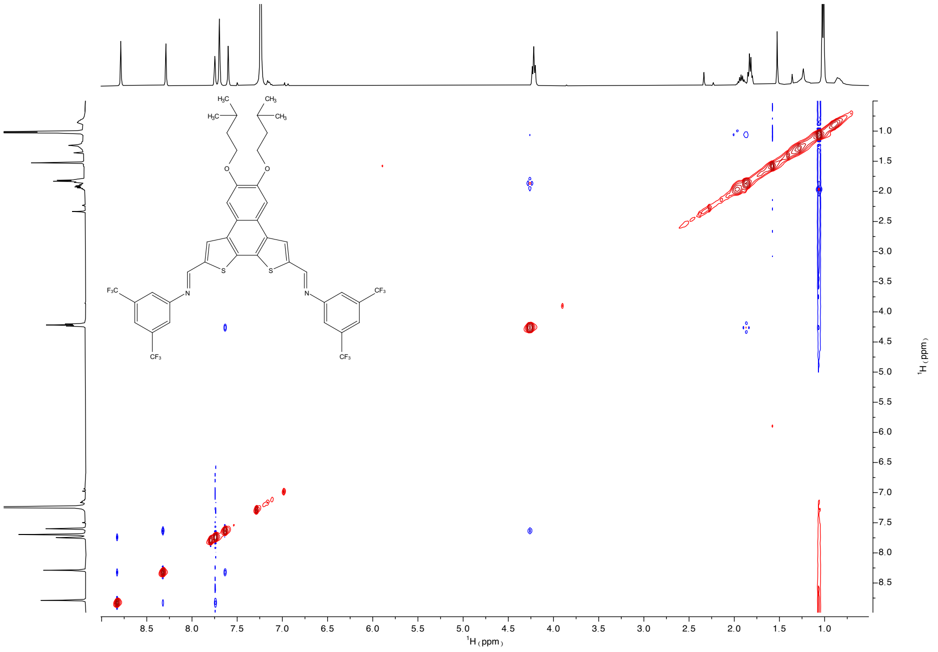

Figure S53. ^{1}H-^{1}H NOESY spectrum of **α(1dd)₂** (CDCl₃, 400 MHz, 298 K).

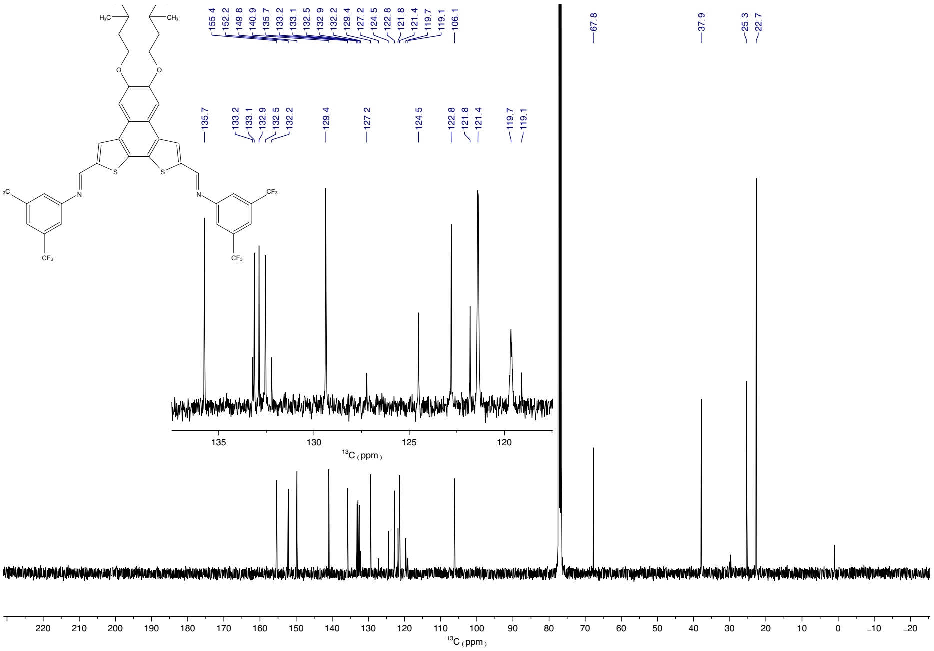

Figure S54. $^{13}C\{^{1}H\}$ NMR spectrum of **α(1dd)₂** (CDCl₃, 101 MHz, 298 K).

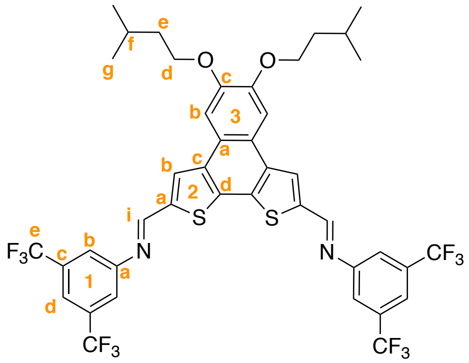

Assignment	^{1}H δ	^{13}C δ
1a	-	152.2
1b	7.71	121.4 (d, *J* = 3.6 Hz)
1c	-	132.9 (q, 33.6 Hz)
1d	7.77	119.7 (t, *J* = 3.8 Hz)
1e	-	122.4 (q, *J* = 273.2 Hz)
i	8.80	155.4
2a	-	140.9
2b	8.30	129.4
2c	-	135.7
2d	-	133.1
3a	-	122.8
3b	7.61	106.1
3c	-	149.8
3d	4.23	67.8
3e	1.84	37.9
3f	1.94	25.3
3g	1.04	22.7

Figure S55. **α(1dd)₂** with assignment of ^{1}H and ^{13}C resonances.

Comparison of remote ^{13}C chemical shifts

The chemical shifts of interior 2b ^{1}H and ^{13}C shift upfield for electron donating groups and downfield for electron withdrawing substituents. The Hammett parameters (σ) for both *para* and *meta* can be contrasted against these 2b resonances.

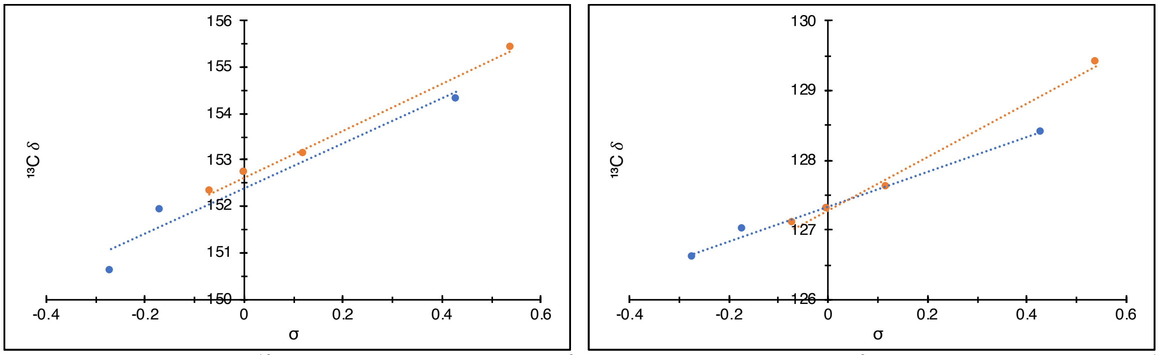

Figure S56. Left: Plot of i ^{13}C resonances vs σ_p (blue, $R^2 = 0.94$) and σ_m (orange, $R^2 = 0.99$). Right: Plot of 2b ^{13}C resonances vs σ_p (blue, $R^2 = 0.99$) and σ_m (orange, $R^2 = 0.99$).

3) Gel Permeation Chromatography (GPC) Traces for α(1)$_2$ Series

Purification of all di-imine products was accomplished with a Shimadzu Nexera Gel Permeation Chromatography (GPC) equipped with a Shodex organic size exclusion column (100 Å styrogel stationary phase, viable molecular weight range of 400–6000 g/mol) and a toluene mobile phase (flow rate 5 mL/min). The highlight region in the below chromatograms represents the target compounds. While this method removes any non-toluene soluble components, the chromatograms demonstrate good conversion to the di-imine species with only trace mono-coupled byproducts. The exceptions to these findings are the electron deficient amine end-caps, **1d** and **1dd**, which have elevated levels of partial coupling byproduct.

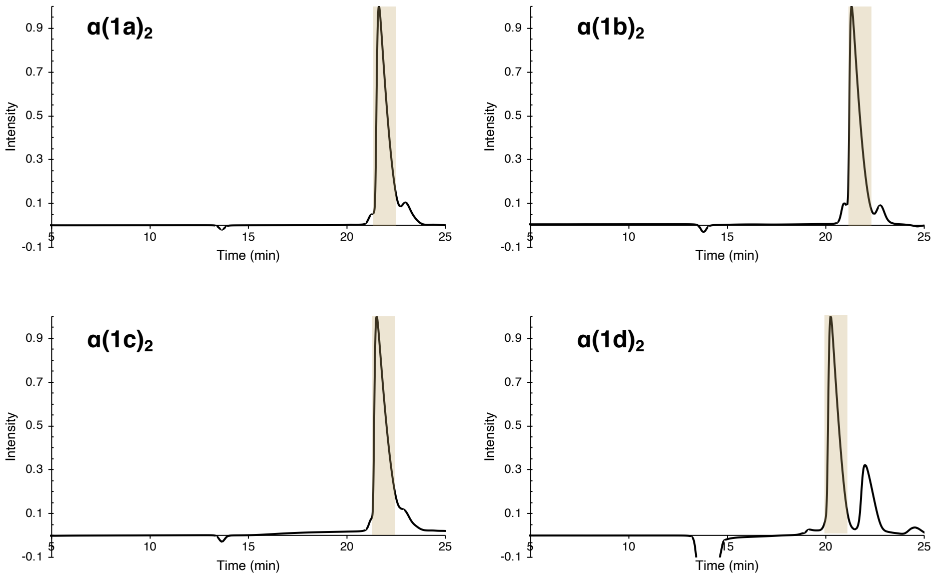

Figure S57. GPC traces of crude reaction mixtures for **α(1a–d)$_2$** series. Each trace shown corresponds to the preferred condensation conditions given in section 1. Highlighted region is the peak of interest.

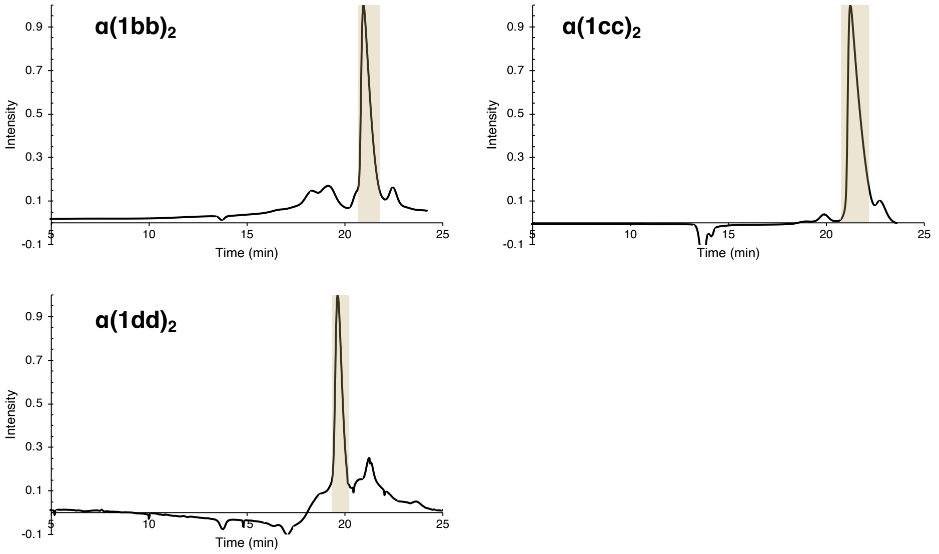

Figure S58. GPC traces of crude reaction mixtures for **α(1bb–dd)$_2$** series. Each trace shown corresponds to the preferred condensation conditions given in Table S1. Highlighted region is the peak of interest.

4) Photophysical Properties α(1)₂ Series

Absorbance spectra were collected on an Agilent Cary Bio100 spectrophotometer and corrected for solvent background absorbance and instrument drift. Emission spectra were recorded on a Horiba Jon-Yvon Flourolog-3 with excitation and emission slit widths set at 2 nm. **α(1a–d)$_2$** and **α(1bb–dd)$_2$**, were prepared with degassed, dry, solvents and degassed prior to sealing in 1 cm path length quartz cuvettes. Emission spectra were analyzed at λ_{ex} = 365 nm. Fluorescence quantum yields were determined for select compounds – **α(1c)$_2$**, **α(1d)$_2$**, **α(1cc)$_2$**, and **α(1dd)$_2$** – by reference to 9,10-diphenylanthracene in cyclohexane (ϕ = 0.91) which was cross-checked with quinine bisulfate in 0.5 M H_2SO_4 (aq) (ϕ = 0.54). Five solutions of varying concentration were prepared for each sample and good fits (R^2 ≥ 0.99) were obtained in all cases. The absorbance of all sample solutions was kept below 0.10 to avoid the inner-filter effect. Measurements were performed at room temperature with both sample and standard excited at the same wavelength (365 nm).[1] All quantum yields obtained were below 1%, with fits for the remainder of the derivatives proving to be of low quality. Plots are given below with UV-visible spectra as solid lines and emission spectra as dashed lines. UV-visible spectra are plotted as molar absorptivity (left vertical axis) vs wavelength (horizontal axis). Emission spectra are plotted as normalized intensities (right vertical axis) vs wavelength (horizontal axis).

***p*-Aryl Series: α(1a)$_2$ vs α(1b–d)$_2$**

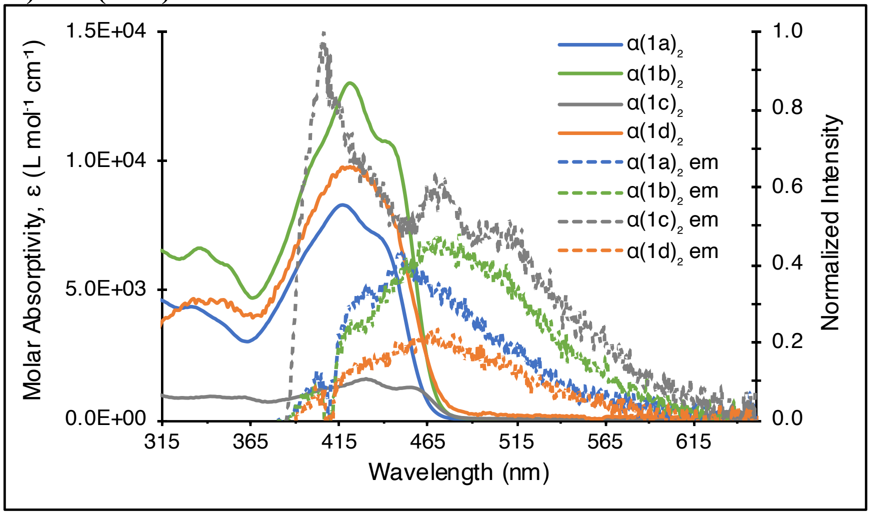

Figure S59. Absorbance and emission spectra for **α(1a)$_2$** and **α(1b–d)$_2$** in toluene.

***m*-Aryl Series: α(1a)$_2$ vs α(1bb–dd)$_2$**

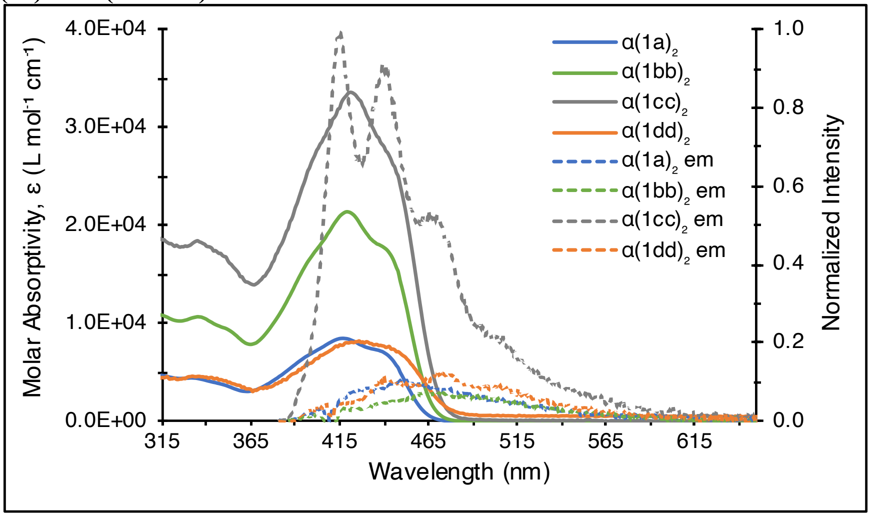

Figure S60. Absorbance and emission spectra for **α(1a)$_2$** and **α(1bb–dd)$_2$** in toluene.

5) Computational Analysis

For the computational analysis, we modeled the seven di-imine derivatives with methoxy solubilizing groups in place of the isopentoxy groups employed in the experimental work, with the abbreviation for the model compounds being *α(1)₂*. Due to the close proximity of the imine nitrogens to the σ-hole of the interior thiophenes sulfur atoms the formation of attractive non-covalent interactions is plausible (i.e. the *cis*-effect is likely to be a factor). With that in mind we carried out computations in Gaussian 16, utilizing the ωB97-XD functional with a cc-pVDZ basis set to accommodate the variety of light atoms employed in this work and account for the likelihood of dispersion interactions, to perform geometry optimizations and frequency calculations on the *α(1)₂* series.[4] Natural Bond Orbital (NBO) analysis was carried out to further validate the strength of the N···S–C interaction. The values obtained for single interactions are given in the tables below and correlate to approximately half of the relative energy difference between conformer-1 (two N···S–C interactions) and conformer-2 (no N···S–C interactions). Cartesian coordinates for all output geometries have been included as a separate .rtf file for ease.

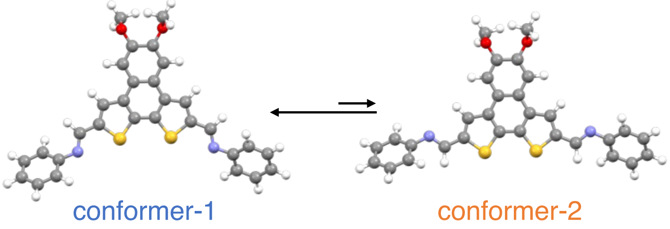

Figure S61. Ball-and-stick representations for optimized conformers of *α(1a)₂*.

Table S2. Computational data for optimized *α(1a)₂* models

Model Compound	Total Energy (E_h)	ZPC[a] (E_h)	Energy$_{\text{Total Energy + ZPC}}$ (E_h)	Rel. Energy[b] (kcal mol^{-1})	IF[c]	NBO$_{N\cdots S–C}$ (kcal mol^{-1})
α(1a)₂ - Conformer 1	-2212.36980437	0.447909	-2211.921895	0.00	0	0.94
α(1a)₂ - Conformer 2	-2212.36519223	0.447855	-2211.917337	+2.86	0	–

[a]ZPC = zero-point correction, [b]Rel. Energy = relative energetic difference between conformer-1 vs conformer-2, [c]IF = number of imaginary frequencies.

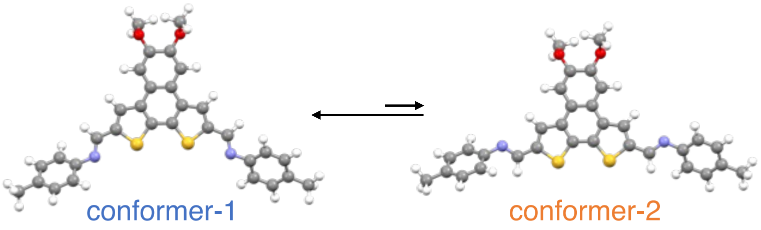

Figure S62. Ball-and-stick representations for optimized conformers of *α(1b)₂*.

Table S3. Computational data for optimized *α(1b)₂* models

Model Compound	Total Energy (E_h)	ZPC[a] (E_h)	Energy$_{\text{Total Energy + ZPC}}$ (E_h)	Rel. Energy[b] (kcal mol^{-1})	IF[c]	NBO$_{N\cdots S–C}$ (kcal mol^{-1})
α(1b)₂ - Conformer 1	-2290.9848472	0.502267	-2290.48258	0.00	0	0.95
α(1b)₂ - Conformer 2	-2290.98022711	0.502186	-2290.478041	+2.84	0	–

[a]ZPC = zero-point correction, [b]Rel. Energy = relative energetic difference between conformer-1 vs conformer-2, [c]IF = number of imaginary frequencies.

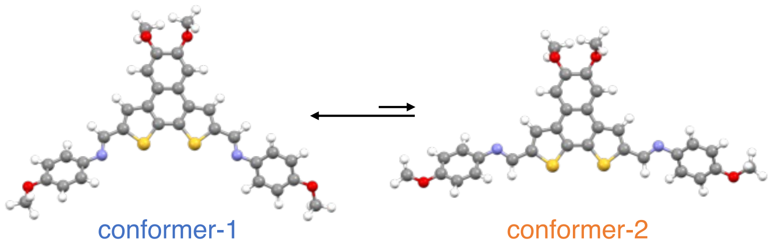

Figure S63. Ball-and-stick representations for optimized conformers of *α(1c)₂*.

Table S4. Computational data for optimized *α(1c)₂* models

Model Compound	Total Energy (E_h)	ZPC[a] (E_h)	$Energy_{Total\ Energy + ZPC}$ (E_h)	Rel. Energy[b] (kcal mol^{-1})	IF[c]	$NBO_{N\cdots S–C}$ (kcal mol^{-1})
α(1c)₂ - Conformer 1	-2441.35850602	0.513194	-2440.845312	0.00	0	0.99
α(1c)₂ - Conformer 2	-2441.35373844	0.513132	-2440.840606	+2.95	0	–

[a]ZPC = zero-point correction, [b]Rel. Energy = relative energetic difference between conformer-1 vs conformer-2, [c]IF = number of imaginary frequencies.

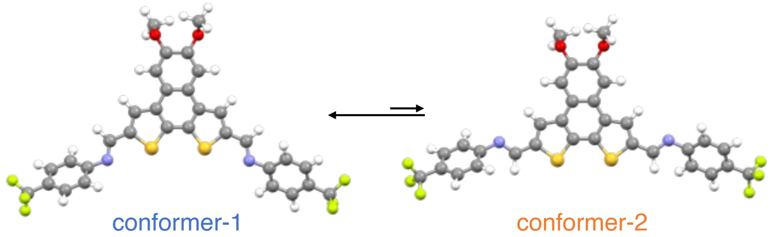

Figure S64. Ball-and-stick representations for optimized conformers of *α(1d)₂*.

Table S5. Computational data for optimized *α(1d)₂* models

Model Compound	Total Energy (E_h)	ZPC[a] (E_h)	$Energy_{Total\ Energy + ZPC}$ (E_h)	Rel. Energy[b] (kcal mol^{-1})	IF[c]	$NBO_{N\cdots S–C}$ (kcal mol^{-1})
α(1d)₂ - Conformer 1	-2886.32153091	0.457726	-2885.863805	0.00	0	0.90
α(1d)₂ - Conformer 2	-2886.31687113	0.457661	-2885.85921	+2.88	0	–

[a]ZPC = zero-point correction, [b]Rel. Energy = relative energetic difference between conformer-1 vs conformer-2, [c]IF = number of imaginary frequencies.

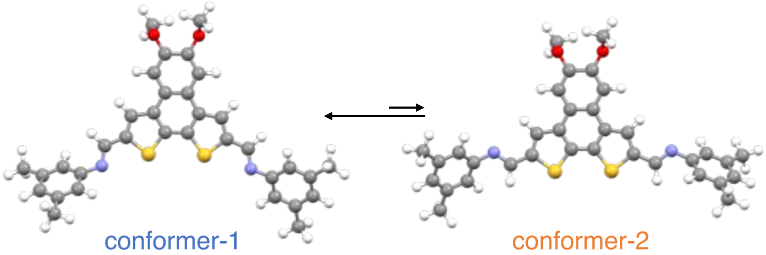

Figure S65. Ball-and-stick representations for optimized conformers of *α(1bb)₂*.

Table S6. Computational data for optimized *α(1bb)₂* models

Model Compound	Total Energy (E_h)	ZPC[a] (E_h)	$Energy_{Total\ Energy\ +\ ZPC}$ (E_h)	Rel. Energy[b] (kcal mol^{-1})	IF[c]	$NBO_{N\cdots S\text{–}C}$ (kcal mol^{-1})
α(1bb)₂ – Conformer 1	-2369.60097548	0.556987	-2369.043988	0.00	0	0.94
α(1bb)₂ – Conformer 2	-2369.59643228	0.556775	-2369.039657	+2.72	0	–

[a]ZPC = zero-point correction, [b]Rel. Energy = relative energetic difference between conformer-1 vs conformer-2, [c]IF = number of imaginary frequencies.

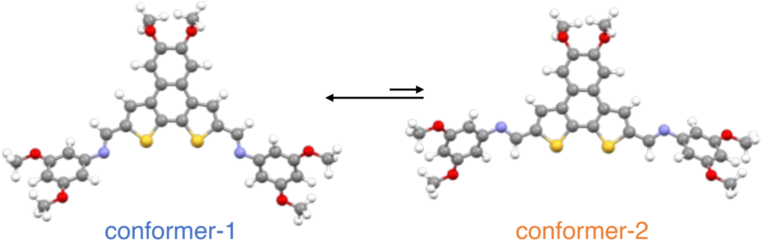

Figure S66. Ball-and-stick representations for optimized conformers of *α(1cc)₂*.

Table S7. Computational data for optimized *α(1cc)₂* models

Model Compound	Total Energy (E_h)	ZPC[a] (E_h)	$Energy_{Total\ Energy\ +\ ZPC}$ (E_h)	Rel. Energy[b] (kcal mol^{-1})	IF[c]	$NBO_{N\cdots S\text{–}C}$ (kcal mol^{-1})
α(1cc)₂ - Conformer 1	-2670.3487298	0.578006	-2669.770724	0.00	0	0.94
α(1cc)₂ - Conformer 2	-2670.34426905	0.578046	-2669.766223	+2.82	0	–

[a]ZPC = zero-point correction, [b]Rel. Energy = relative energetic difference between conformer-1 vs conformer-2, [c]IF = number of imaginary frequencies.

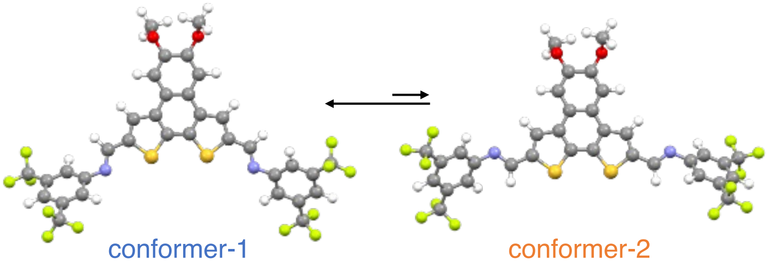

Figure S67. Ball-and-stick representations for optimized conformers of *α(1dd)₂*.

Table S8. Computational data for optimized *α(1dd)$_2$* models

Model Compound	Total Energy (E_h)	ZPC[a] (E_h)	Energy$_{Total\ Energy\ +\ ZPC}$ (E_h)	Rel. Energy[b] (kcal mol^{-1})	IF[c]	NBO$_{N\cdots S–C}$ (kcal mol^{-1})
α(1dd)$_2$ - Conformer 1	-3560.26816949	0.467056	-3559.801113	0.00	0	0.87
α(1dd)$_2$ - Conformer 2	-3560.26344817	0.467127	-3559.796321	+3.01	0	–

[a]ZPC = zero-point correction, [b]Rel. Energy = relative energetic difference between conformer-1 vs conformer-2, [c]IF = number of imaginary frequencies.

Experimental vs Calculated Chemical Shift Analysis

The optimized structures were subsequently used to calculate the ^{1}H and ^{13}C NMR spectra using the GIAO method with implicit solvent ($CHCl_3$). The isotropic shieldings for the model compounds with the alkoxy groups simplified to methoxy groups were computed for both protons and carbons using the GIAO method with the cc-pVDZ basis set and ωB97-XD functional in Gaussian 16 with implicit solvent ($CHCl_3$). The experimental chemical shifts (ppm) were extracted from ^{1}H–^{13}C HSQC.

α(1a)$_2$

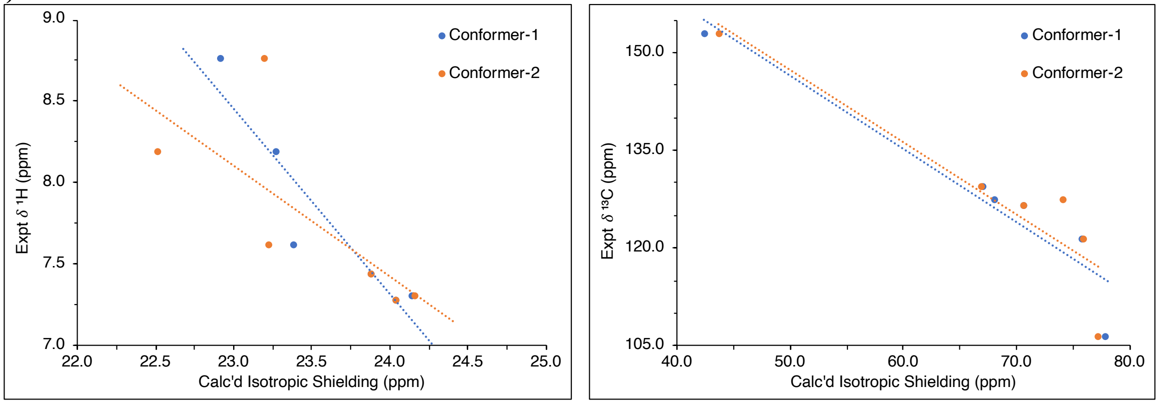

Figure S68. Comparison of experimental vs calculated ^{1}H (left) and ^{13}C (right) resonances (δ) for **α(1a)$_2$** (experimental) and *a(1a)$_2$* (calculated). Conformer 1 is given in blue (^{1}H $R^2 = 0.87$; ^{13}C $R^2 = 0.90$) and Conformer 2 is represented in orange (^{1}H $R^2 = 0.53$; ^{13}C $R^2 = 0.85$).

α(1b)$_2$

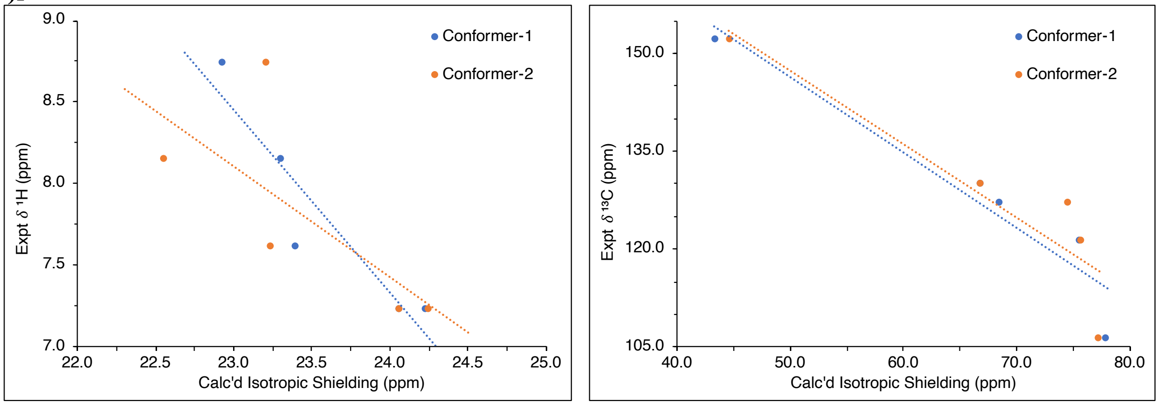

Figure S69. Comparison of experimental vs calculated ^{1}H (left) and ^{13}C (right) resonances (δ) for **α(1b)$_2$** (experimental) and *a(1b)$_2$* (calculated). Conformer 1 is given in blue (^{1}H $R^2 = 0.87$; ^{13}C $R^2 = 0.91$) and Conformer 2 is represented in orange (^{1}H $R^2 = 0.52$; ^{13}C $R^2 = 0.84$).

α(1c)$_2$

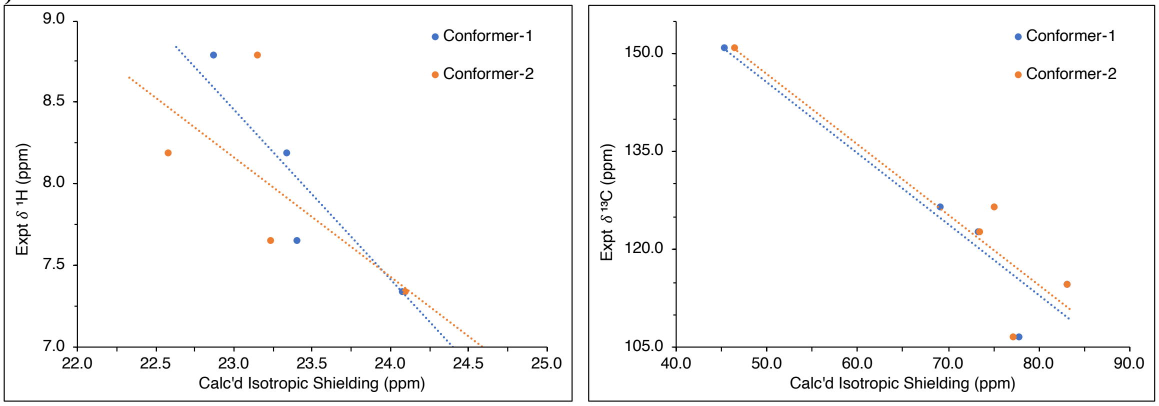

Figure S70. Comparison of experimental vs calculated ^{1}H (left) and ^{13}C (right) resonances (δ) for **α(1c)$_2$** (experimental) and *a(1c)$_2$* (calculated). Conformer 1 is given in blue (^{1}H $R^2 = 0.90$; ^{13}C $R^2 = 0.90$) and Conformer 2 is represented in orange (^{1}H $R^2 = 0.64$; ^{13}C $R^2 = 0.84$).

α(1d)$_2$

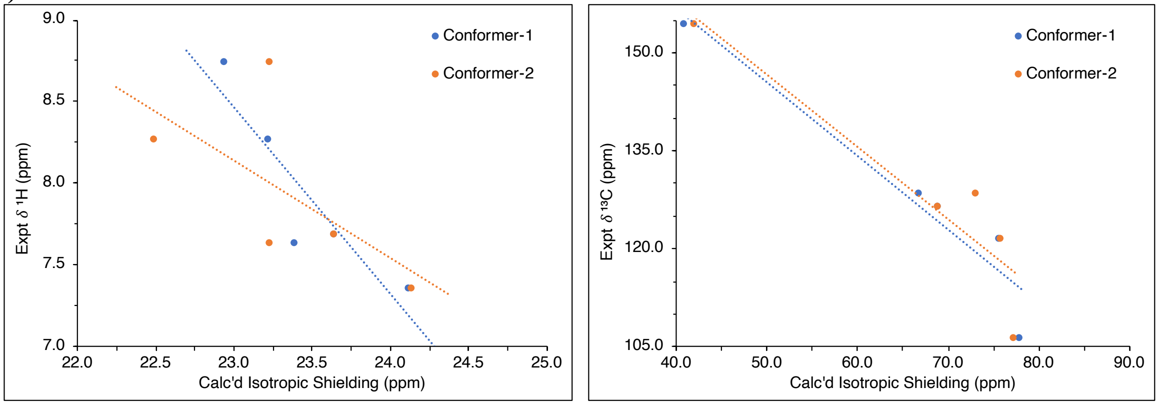

Figure S71. Comparison of experimental vs calculated ^{1}H (left) and ^{13}C (right) resonances (δ) for **α(1d)$_2$** (experimental) and *a(1d)$_2$* (calculated). Conformer 1 is given in blue (^{1}H $R^2 = 0.82$; ^{13}C $R^2 = 0.92$) and Conformer 2 is represented in orange (^{1}H $R^2 = 0.41$; ^{13}C $R^2 = 0.86$).

α(1bb)$_2$

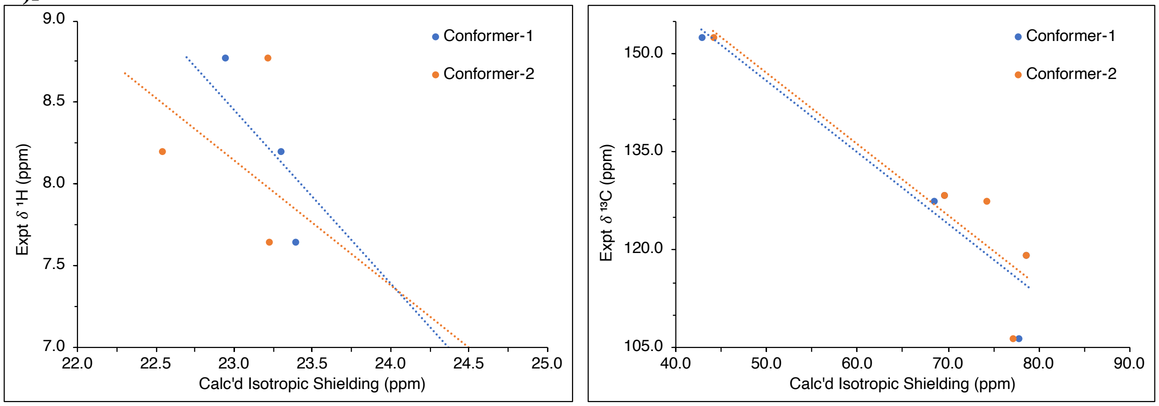

Figure S72. Comparison of experimental vs calculated ^{1}H (left) and ^{13}C (right) resonances (δ) for **α(1bb)$_2$** (experimental) and *a(1bb)$_2$* (calculated). Conformer 1 is given in blue (^{1}H $R^2 = 0.91$; ^{13}C $R^2 = 0.89$) and Conformer 2 is represented in orange (^{1}H $R^2 = 0.67$; ^{13}C $R^2 = 0.84$).

α(1cc)₂

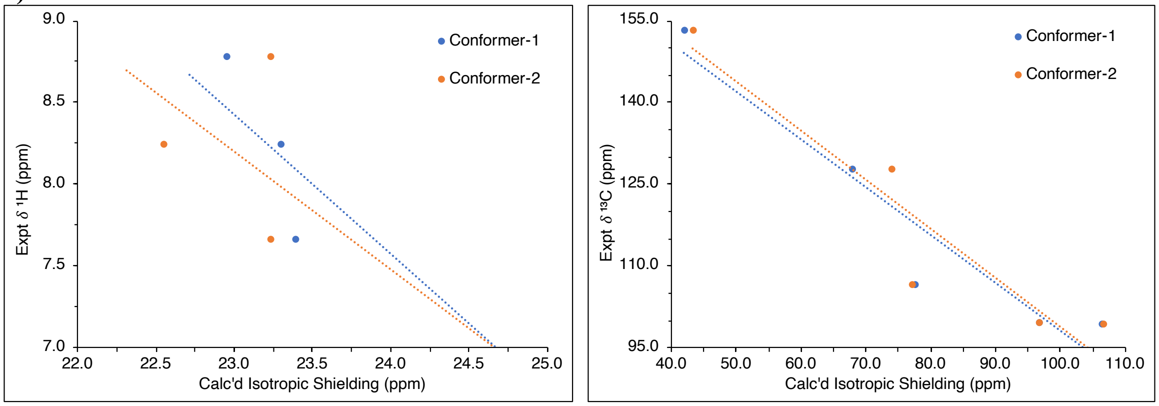

Figure S73. Comparison of experimental vs calculated ^{1}H (left) and ^{13}C (right) resonances (δ) for **α(1cc)₂** (experimental) and *a(1cc)₂* (calculated). Conformer 1 is given in blue (^{1}H R^2 = 0.93; ^{13}C R^2 = 0.91) and Conformer 2 is represented in orange (^{1}H R^2 = 0.82; ^{13}C R^2 = 0.88).

α(1dd)₂

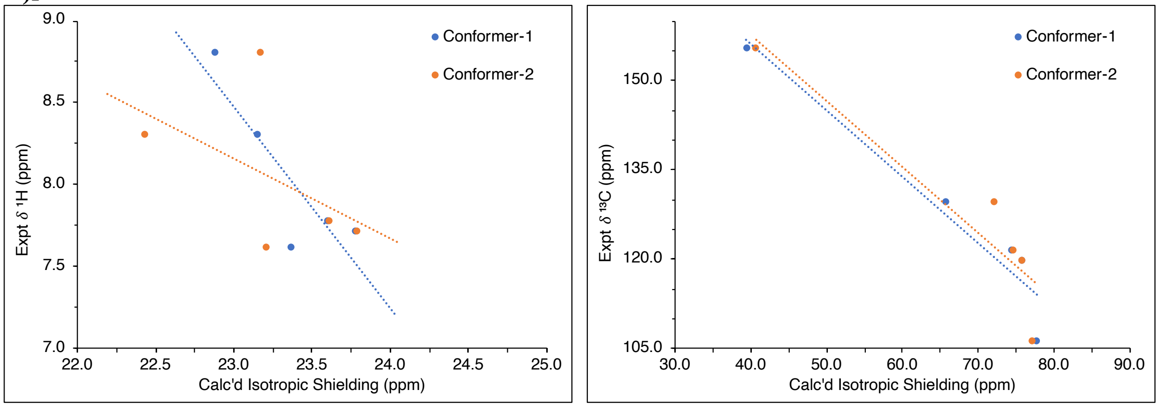

Figure S74. Comparison of experimental vs calculated ^{1}H (left) and ^{13}C (right) resonances (δ) for **α(1dd)₂** (experimental) and *a(1dd)₂* (calculated). Conformer 1 is given in blue (^{1}H R^2 = 0.76; ^{13}C R^2 = 0.93) and Conformer 2 is represented in orange (^{1}H R^2 = 0.25; ^{13}C R^2 = 0.87).

6) Crystallographic Analysis

Crystallographic analysis and data for **α(1b)₂**, **α(1c)₂**, **α(1d)₂**, **α(1cc)₂**, and **α(1a)(CHO)** are presented below. Data for **α(1b)₂**, **α(1c)₂**, **α(1d)₂**, and **α(1a)(CHO)** were collected from a single crystal at 150(2) K on a Bruker AXS D8 Quest four circle diffractometer with an I-mu-S 3.0 microsource X-ray tube using HELIOS multilayer Montel optics as monochromator and a PhotonIII_C14 charge-integrating and photon counting pixel array detector. The diffractometer used CuK_α radiation (λ = 1.54178 Å). Data for **α(1cc)₂** were collected from a single crystal at 150(2) K on a Bruker AXS D8 Quest three circle diffractometer with a fine focus sealed tube X-ray source using a Triumph curved graphite crystal as monochromator and a PhotonII charge-integrating pixel array (CPAD) detector. The diffractometer used MoK_α radiation (λ = 0.71073 Å). All data were integrated with SAINT V8.40B and a multi-scan absorption correction using SADABS 2016/2 was applied.[5,6] The structures were solved by dual methods with SHELXT and refined by full-matrix least-squares methods against F^2 using SHELXL-2019/2.[7,8] All non-hydrogen atoms were refined with anisotropic displacement parameters. All hydrogen atoms were refined isotropic on calculated positions using a riding model with their U_{iso} values constrained to 1.5 times the U_{eq} of their pivot atoms for terminal sp^3 carbon atoms and 1.2 times for all other carbon atoms. Tables and general reports were generated with FinalCif and edited accordingly.[9] Any additional refinement notes are detailed below, all structures have been deposited in the Cambridge Crystallographic Data Centre (CCDC)[10] with their deposition numbers given (2477200–2477204).

α(1b)₂

Single crystals of **α(1b)₂** were obtained through vapor diffusion of petroleum ether into a saturated solution of **α(1b)₂** in THF (Figure S75). The two alkyl substituents were disordered over each two orientations and were restrained to have similar geometries. U_{ij} components of ADPs for disordered atoms closer to each other than 2.0 Å were restrained to be similar. Subject to these conditions the occupancy ratio refined to 0.390(19) to 0.610(19) and to 0.709(4) to 0.291(4). The structure contains 191 Å^3 of solvent accessible pore areas. Due to the disorder of the solvate molecules (major components of petroleum ether are pentane, isopentane, 3-methylpentane and *n*-hexane) the structure factors were instead augmented via reverse Fourier transform methods using the SQUEEZE routine[11] as implemented in the program Platon. The resultant FAB file containing the structure factor contribution from the electron content of the void space was used in together with the original hkl file in the further refinement. (The FAB file with details of the Squeeze results is appended to the cif file). The Squeeze procedure corrected for 74 electrons within the solvent accessible voids. CCDC number: 2477202

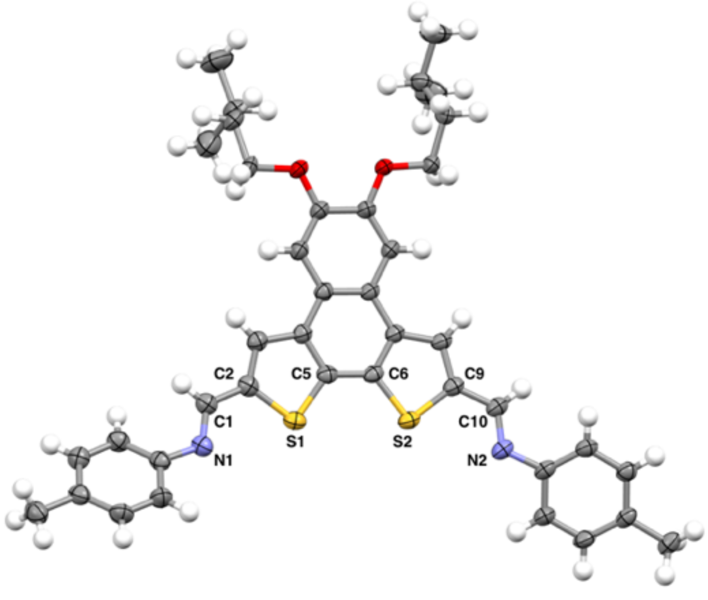

Figure S75. ORTEP diagram of **α(1b)₂** (ellipsoids drawn at 50% probability). Select bond lengths (Å), distances (Å), and angles (°): N1···S1, 3.045(2); N2···S2, 2.959(2); C5–S1···N1, 141.69(6); C9–S2···N2, 143.63(6); N1–C1–C2–S1, 1.0(2); N2–C10–C9–S2, 0.2(2).

Table S9. Crystallographic data and structural refinement parameters, α(1b)₂.

	**α(1b)$_2$**
Empirical formula	$C_{40}H_{42}N_2O_2S_2$
Formula weight	646.87
Temperature [K]	150(2)
Crystal system	triclinic
Space group (number)	$P\bar{1}$ (2)
a [Å]	5.1111(2)
b [Å]	17.6434(5)
c [Å]	21.0008(7)
α [°]	87.960(2)
β [°]	83.543(2)
γ [°]	83.451(2)
Volume [Å^3]	1869.00(11)
Z	2
ρ_{calc} [gcm^{-3}]	1.149
μ [mm^{-1}]	1.554
F(000)	688
Crystal size [mm^3]	0.040×0.050×0.660
Crystal color	yellow
Crystal shape	needle
Radiation	CuK_α (λ=1.54178 Å)
2θ range [°]	4.24 to 160.11 (0.78 Å)
Index ranges	$-6 \le h \le 5$<br>$-22 \le k \le 22$<br>$-26 \le l \le 26$
Reflections collected	34435
Independent reflections	7954<br>R_{int} = 0.0535<br>R_{sigma} = 0.0424
Completeness to θ = 25.242°	99.9 %
Data / Restraints / Parameters	7954 / 366 / 535
Absorption correction T_{min}/T_{max} (method)	0.5530 / 0.7543 (multi-scan)
Goodness-of-fit on F^2	1.077
Final *R* indexes<br>[$I \geq 2\sigma(I)$]	R_1 = 0.0441<br>wR_2 = 0.1189
Final *R* indexes<br>[all data]	R_1 = 0.0531<br>wR_2 = 0.1235
Largest peak/hole [eÅ^{-3}]	0.56/−0.22

α(1c)₂

Single crystals of **α(1c)₂** were obtained through vapor diffusion of petroleum ether into a saturated solution of **α(1c)₂** in THF (Figure S76). The structure contains additional 943 $Å^3$ of solvent accessible pores (~12% of the crystal volume). The residual electron density peaks are not arranged in an interpretable pattern (the antisolvent used was petroleum ether and the content of the voids appears to consist of a highly disordered mixture of various low chain alkanes). The structure factors were instead augmented via reverse Fourier transform methods using the SQUEEZE routine[11] as implemented in the program Platon. The resultant FAB file containing the structure factor contribution from the electron content of the void space was used in together with the original hkl file in the further refinement. (The FAB file with details of the Squeeze results is appended to the cif file). The Squeeze procedure corrected for 240 electrons within the solvent accessible pores. CCDC number: 2477203

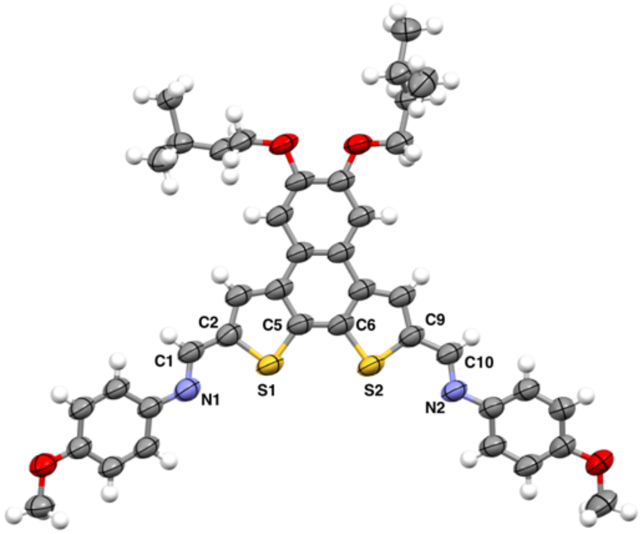

Figure S76. ORTEP diagram of **α(1c)₂** (ellipsoids drawn at 50% probability). Select bond lengths (Å), distances (Å), and angles (°): N1···S1, 2.967(5); N2···S2, 2.991(4); C5–S1···N1, 143.5(2); C6–S2···N2, 142.9(2); N1–C1–C2–S1, 0.0(7); N2–C10–C9–S2, -5.1(7).

Table S10. Crystallographic data and structural refinement parameters, α(1c)₂.

	**α(1c)$_2$**
Empirical formula	$C_{40}H_{42}N_2O_4S_2$
Formula weight	678.87
Temperature [K]	150(2)
Crystal system	orthorhombic
Space group (number)	*Pbca* (61)
a [Å]	20.903(3)
b [Å]	8.026(2)
c [Å]	45.905(10)
α [°]	90
β [°]	90
γ [°]	90
Volume [Å^3]	7701(3)
Z	8
ρ_{calc} [gcm^{-3}]	1.171
μ [mm^{-1}]	1.571
F(000)	2880
Crystal size [mm^3]	0.030×0.090×0.130
Crystal color	yellow
Crystal shape	needle
Radiation	CuK_α (λ=1.54178 Å)
2θ range [°]	5.72 to 159.97 (0.78 Å)
Index ranges	$-26 \le h \le 19$<br>$-9 \le k \le 9$<br>$-57 \le l \le 57$
Reflections collected	49479
Independent reflections	8204<br>R_{int} = 0.1764<br>R_{sigma} = 0.1533
Completeness to θ = 25.242°	99.9 %
Data / Restraints / Parameters	8204 / 0 / 440
Absorption correction T_{min}/T_{max} (method)	0.5426 / 0.7543 (multi-scan)
Goodness-of-fit on F^2	0.967
Final *R* indexes [$I \ge 2\sigma(I)$]	R_1 = 0.0840<br>wR_2 = 0.2305
Final *R* indexes [all data]	R_1 = 0.1489<br>wR_2 = 0.2911
Largest peak/hole [eÅ^{-3}]	0.34/−0.34
Extinction coefficient	0.00090(16)

α(1d)$_2$

Single crystals of **α(1d)$_2$** were obtained through vapor diffusion of petroleum ether into a saturated solution of **α(1d)$_2$** in dichloromethane (Figure S77). The structure was refined as a 2-component inversion twin. CCDC number: 2477200

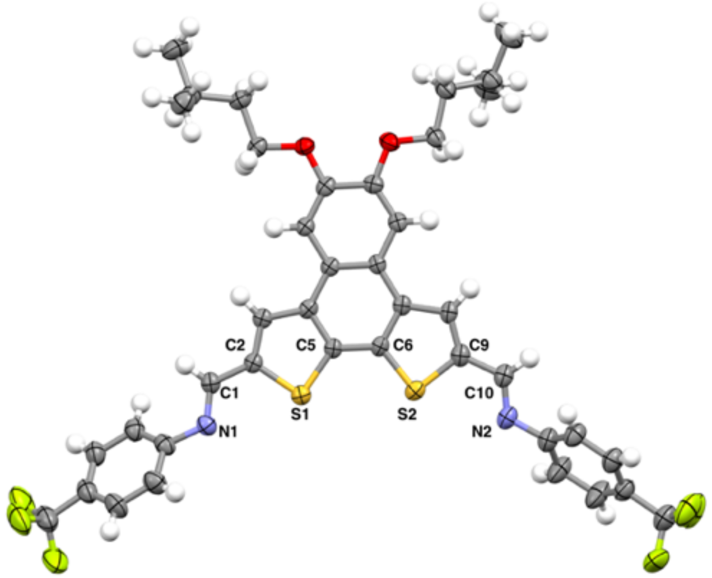

Figure S77. ORTEP diagram of **α(1d)$_2$** (ellipsoids drawn at 50% probability). Select bond lengths (Å), distances (Å), and angles (°): N1···S1, 2.993(7); N2···S2, 2.898(6); C5–S1···N1, 142.3(2); C9–S2···N2, 144.7(2); N1–C1–C2–S1, 2.4(7); N2–C10–C9–S2, 7.4(7).

Table S11. Crystallographic data and structural refinement parameters, α(1d)₂.

	**α(1d)$_2$**
Empirical formula	$C_{40}H_{36}F_6N_2O_2S_2$
Formula weight	754.83
Temperature [K]	150(2)
Crystal system	trigonal
Space group (number)	$P3_2$ (145)
a [Å]	17.665(2)
b [Å]	17.665(2)
c [Å]	10.1037(11)
α [°]	90
β [°]	90
γ [°]	120
Volume [Å^3]	2730.4(8)
Z	3
ρ_{calc} [gcm^{-3}]	1.377
μ [mm^{-1}]	1.923
F(000)	1176
Crystal size [mm^3]	0.030×0.040×0.230
Crystal color	yellow
Crystal shape	needle
Radiation	CuK_α (λ=1.54178 Å)
2θ range [°]	5.78 to 160.51 (0.78 Å)
Index ranges	$-20 \le h \le 22$<br>$-22 \le k \le 22$<br>$-12 \le l \le 9$
Reflections collected	22290
Independent reflections	6762<br>R_{int} = 0.0587<br>R_{sigma} = 0.0638
Completeness to θ = 25.242°	99.9 %
Data / Restraints / Parameters	6762 / 1 / 475
Absorption correction T_{min}/T_{max} (method)	0.5206 / 0.7543 (multi-scan)
Goodness-of-fit on F^2	1.066
Final R indexes<br>[$I \geq 2\sigma(I)$]	R_1 = 0.0522<br>wR_2 = 0.1254
Final R indexes<br>[all data]	R_1 = 0.0633<br>wR_2 = 0.1330
Largest peak/hole [eÅ^{-3}]	0.37/−0.26
Extinction coefficient	0.0019(3)
Flack X parameter	0.49(3)

α(1cc)₂

Single crystals of **α(1cc)₂** were obtained through vapor diffusion of petroleum ether into a saturated solution of **α(1cc)₂** in THF (Figure S78). CCDC number: 2477204

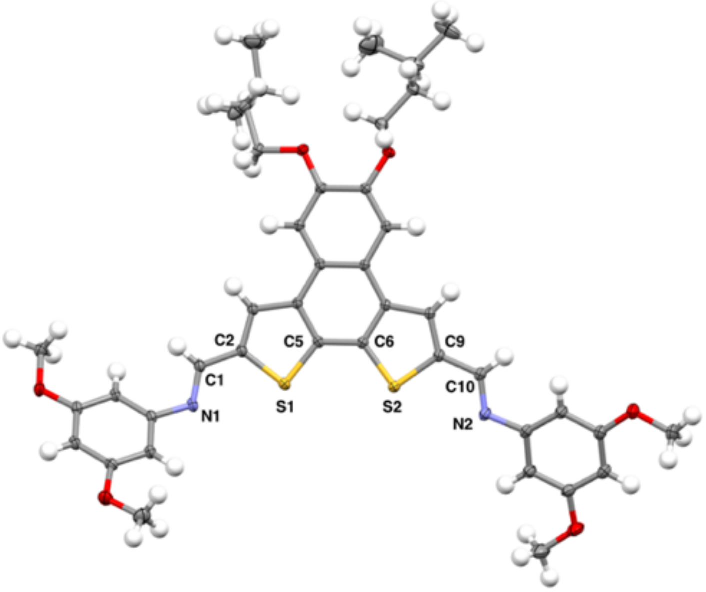

Figure S78. ORTEP diagram of **α(1cc)₂** (ellipsoids drawn at 50% probability). Select bond lengths (Å), distances (Å), and angles (°): N1···S1, 3.071(1); N2···S2, 3.030(1); C5–S1···N1, 140.12(4); C6–S2···N2, 142.44(4); N1–C1–C2–S1, 1.2(1); N2–C10–C9–S2, 1.3(1).

Table S12. Crystallographic data and structural refinement parameters, α(1cc)$_2$.

	**α(1cc)$_2$**
Empirical formula	$C_{42}H_{46}N_2O_6S_2$
Formula weight	738.93
Temperature [K]	150(2)
Crystal system	monoclinic
Space group (number)	$P2_1/c$ (14)
a [Å]	11.082(3)
b [Å]	36.517(9)
c [Å]	9.3470(16)
α [°]	90
β [°]	98.235(14)
γ [°]	90
Volume [Å^3]	3743.7(16)
Z	4
ρ_{calc} [gcm^{-3}]	1.311
μ [mm^{-1}]	0.193
F(000)	1568
Crystal size [mm^3]	0.080×0.130×0.410
Crystal color	yellow
Crystal shape	plate
Radiation	MoK_α (λ=0.71073 Å)
2θ range [°]	4.46 to 66.30 (0.65 Å)
Index ranges	$-17 \le h \le 16$<br>$-49 \le k \le 56$<br>$-14 \le l \le 9$
Reflections collected	65863
Independent reflections	13970<br>R_{int} = 0.0344<br>R_{sigma} = 0.0248
Completeness to θ = 25.242°	99.9 %
Data / Restraints / Parameters	13970 / 0 / 477
Absorption correction T_{min}/T_{max} (method)	0.6743 / 0.7465 (multi-scan)
Goodness-of-fit on F^2	1.029
Final *R* indexes<br>[$I \geq 2\sigma(I)$]	R_1 = 0.0352<br>wR_2 = 0.0954
Final *R* indexes<br>[all data]	R_1 = 0.0459<br>wR_2 = 0.1007
Largest peak/hole [eÅ^{-3}]	0.49/−0.23

Attempted crystallization of α(1a)₂: partial hydrolysis to α(1a)(CHO)

Single crystals of **α(1a)(CHO)** were obtained through vapor diffusion of petroleum ether into a saturated solution of **$α(1a)_2$** in chloroform (Figure S79). Over the course of two days crystals formed that were of the partially hydrolyzed **α(1a)(CHO)**. The structure was refined as a 2-component inversion twin. CCDC number: 2477201

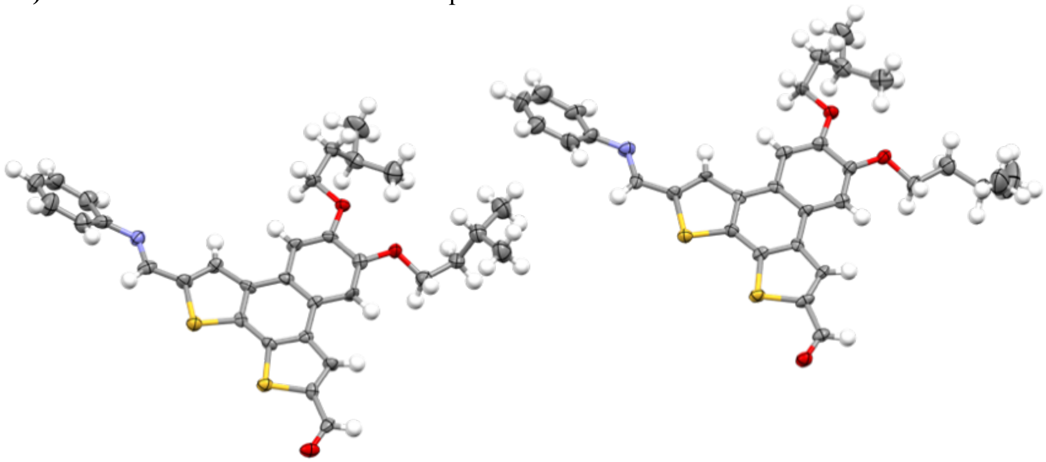

Figure S79. ORTEP diagram of **α(1a)(CHO)** (ellipsoids drawn at 50% probability). Average select bond lengths (Å), distances (Å), and angles (°): S1···O1, 3.028; C5–S1···O1, 141.5; O1–C1–C2–S1, 0.0; S2–C9–C10–N1, 175.9.

Table S13. Crystallographic data and structural refinement parameters, α(1a)(CHO).

	**α(1a)(CHO)**
Empirical formula	$C_{32}H_{33}NO_3S_2$
Formula weight	543.71
Temperature [K]	150(2)
Crystal system	monoclinic
Space group (number)	*Pn* (7)
a [Å]	5.0375(13)
b [Å]	37.547(7)
c [Å]	14.793(4)
α [°]	90
β [°]	95.803(14)
γ [°]	90
Volume [Å^3]	2783.7(11)
Z	4
ρ_{calc} [gcm^{-3}]	1.297
μ [mm^{-1}]	2.001
F(000)	1152
Crystal size [mm^3]	0.001×0.050×0.080
Crystal color	green
Crystal shape	flake
Radiation	CuK_α (λ=1.54178 Å)
2θ range [°]	4.71 to 160.40 (0.78 Å)
Index ranges	$-5 \le h \le 5$<br>$-47 \le k \le 46$<br>$-14 \le l \le 18$
Reflections collected	20114
Independent reflections	8745<br>R_{int} = 0.1225<br>R_{sigma} = 0.1758
Completeness to θ = 25.242°	98.4 %
Data / Restraints / Parameters	8745 / 2 / 694
Absorption correction T_{min}/T_{max} (method)	0.5806 / 0.7543 (multi-scan)
Goodness-of-fit on F^2	0.981
Final *R* indexes [$I \ge 2\sigma(I)$]	R_1 = 0.0711<br>wR_2 = 0.1430
Final *R* indexes [all data]	R_1 = 0.1700<br>wR_2 = 0.1841
Largest peak/hole [eÅ^{-3}]	0.37/−0.35
Flack X parameter	0.10(3)

7) Author Contributions

Emmanuel B.A. Adusei – Carried out the synthesis, characterization, and spectral analysis of all target compounds; wrote the original draft
Sarah Ibrahim – Characterization and spectral analysis of select target compounds
Kara Jenneker – Synthesis and characterization of select target compounds
Calvin D. Goldsmith – Synthesis and characterization of select target compounds
Danielle Dragoi – Synthesis and characterization of select target compounds
Matthias Zeller – Crystallographic analysis
Zacharias J. Kinney – Conceptualized the project; acquired funding; carried out characterization and spectral analysis of all target compounds; wrote the original draft

8) References

(1) Brouwer, A. M. Standards for Photoluminescence Quantum Yield Measurements in Solution (IUPAC Technical Report). *Pure Appl. Chem.* 2011, **83**, 2213–2228.

(2) Huang, C.; Zhen, C. G.; Ping Su, S.; Vijila, C.; Balakrisnan, B.; Joong Auch, M. D.; Ping Loh, K.; Chen, Z. K. Highly Efficient Electroluminescent Biphenylyl-Substituted Poly(p-Phenylenevinylene)s through Fine Tuning the Polymer Structure. *Polymer* 2006, **47**, 1820–1829.

(3) Adusei, E. B. A.; Casetti, V. T.; Goldsmith, C. D.; Caswell, M.; Alinj, D.; Park, J.; Zeller, M.; Rusakov, A. A.; Kinney, Z. J. Bent Naphthodithiophenes: Synthesis and Characterization of Isomeric Fluorophores. *RSC Adv.* 2024, **14**, 25120–25129.

(4) Frisch, M. J.; Trucks, G. W.; Schlegel, H. B.; Scuseria, G. E.; Robb, M. A.; Cheeseman, J. R.; Scalmani, G.; Barone, V.; Petersson, G. A.; Nakatsuji, H.; Li, X.; Caricato, M.; Marenich, A. V.; Bloino, J.; Janesko, B. G.; Gomperts, R.; Mennucci, B.; Hratchian, H. P.; Ortiz, J. V.; Izmaylov, A. F.; Sonnenberg, J. L.; Williams-Young, D.; Ding, F.; Lipparini, F.; Egidi, F.; Goings, J.; Peng, B.; Petrone, A.; Henderson, T.; Ranasinghe, D.; Zakrzewski, V. G.; Gao, J.; Rega, N.; Zheng, G.; Liang, W.; Hada, M.; Ehara, M.; Toyota, K.; Fukuda, R.; Hasegawa, J.; Ishida, M.; Nakajima, T.; Honda, Y.; Kitao, O.; Nakai, H.; Vreven, T.; Throssell, K.; Montgomery, J. A., J.; Peralta, J. E.; Ogliaro, F.; Bearpark, M. J.; Heyd, J. J.; Brothers, E. N.; Kudin, K. N.; Staroverov, V. N.; Keith, T. A.; Kobayashi, R.; Normand, J.; Raghavachari, K.; Rendell, A. P.; Burant, J. C.; Iyengar, S. S.; Tomasi, J.; Cossi, M.; Millam, J. M.; Klene, M.; Adamo, C.; Cammi, R.; Ochterski, J. W.; Martin, R. L.; Morokuma, K.; Farkas, O.; Foresman, J. B.; Fox, D. J. Gaussian 16. Gaussian, Inc.: Wallingford CT 2016.

(5) Bruker. SAINT.

(6) Krause, L.; Herbst-Irmer, R.; Sheldrick, G. M.; Stalke, D. Comparison of Silver and Molybdenum Microfocus X-Ray Sources for Single-Crystal Structure Determination. *J. Appl. Crystallogr.* 2015, **48**, 3–10.

(7) Sheldrick, G. M. SHELXT - Integrated Space-Group and Crystal-Structure Determination. *Acta Crystallogr. Sect. A Found. Crystallogr.* 2015, **71**, 3–8.

(8) Sheldrick, G. M. Crystal Structure Refinement with SHELXL. *Acta Crystallogr. Sect. C Struct. Chem.* 2015, **71**, 3–8.

(9) Kratzert, D. FinalCif, V123 https://dkratzert.de/finalcif.html.

(10) Groom, C. R.; Bruno, I. J.; Lightfoot, M. P.; Ward, S. C. The Cambridge Structural Database. *Acta Crystallogr. Sect. B Struct. Sci. Cryst. Eng. Mater.* 2016, **72**, 171–179.

(11) van der Sluis, P.; Spek, A. L. BYPASS: An Effective Method for the Refinement of Crystal Structures Containing Disordered Solvent Regions. *Acta Crystallogr. A46* 1990, 194–201.
