# Supplementary material for: Intramolecular Stabilization of Naphtho[2,1‑b:3,4‑b′]dithiophenes: Synthesis and Analysis of Imine-Based Chromophores
Source: J Org Chem. 2025 Dec 16;91(1):394–400. doi: 10.1021/acs.joc.5c02486 (PMC12797285; doi:10.1021/acs.joc.5c02486)
Supplement: Supplementary file 2 [file jo5c02486_si_002.rtf]

Intramolecular Stabilization of Naphtho[2,1-b:3,4-b’]dithiophenes: Synthesis and Analysis of Imine-Based ChromophoresSupporting InformationEmmanuel B. A. Adusei, Sarah Ibrahim, Kara Jenneker, Calvin D. Goldsmith, Danielle Dragoi, Matthias Zeller, and Zacharias J. Kinney*Optimized Geometries for ɑ1)2 Series. To locate a conformer use the 1a-d or 1bb-dd code and choose the conformation of interest. ɑ1a)2 Conformer-1C    0.00000000  0.00000000  0.00000000O    1.19971600 -0.11322700 -0.75806400C    1.19424700  0.59403400 -1.92912000C    1.19202600 -0.08696400 -3.12678400C    1.21052000  0.59401600 -4.36274500C    1.21317400  2.01273800 -4.36274500C    1.23166800  2.69371800 -3.12678400C    1.22944700  2.01272000 -1.92912000O    1.22397800  2.71998100 -0.75806400C    2.42369400  2.60675400  0.00000000H    2.28168300  3.22232600  0.89696600H    3.28259300  2.99283400 -0.57482700H    2.61756400  1.56295100  0.29186700H    1.25071400  3.78293600 -3.08388600C    1.21199800  2.71683600 -5.62450200C    1.21169600  2.01267400 -6.82982700C    1.21199800  0.59408000 -6.82982700C    1.21169600 -0.11008200 -5.62450200C    1.21531600 -1.52171300 -5.85222800H    1.21874400 -2.27403800 -5.06423400C    1.21710300 -1.85423400 -7.17924800S    1.21442800 -0.45144600 -8.21856100C    1.22221600 -3.19271200 -7.74518500H    1.22366000 -4.02193100 -7.01431000N    1.23908200 -3.37725000 -9.00811900C    1.19865500 -4.69193900 -9.50983100C    2.02103900 -5.00001900 -10.6010100C    2.02217100 -6.28303300 -11.1375350C    1.18193800 -7.26697600 -10.6135080C    0.33866800 -6.95592400 -9.54852600C    0.34442400 -5.67703800 -8.99526900H   -0.34040300 -5.42821600 -8.18182000H   -0.33772700 -7.71289000 -9.14699800H    1.17425800 -8.26929300 -11.0447960H    2.67782300 -6.51552500 -11.9786920H    2.66138500 -4.21583500 -11.0072060S    1.20926600  3.05820000 -8.21856100C    1.20659100  4.46098800 -7.17924800C    1.20837800  4.12846700 -5.85222800H    1.20495000  4.88079200 -5.06423400C    1.20147800  5.79946600 -7.74518500H    1.20003400  6.62868500 -7.01431000N    1.18461200  5.98400400 -9.00811900C    1.22503900  7.29869300 -9.50983100C    2.07927000  8.28379200 -8.99526900C    2.08502600  9.56267800 -9.54852600C    1.24175600  9.87373000 -10.6135080C    0.40152300  8.88978700 -11.1375350C    0.40265500  7.60677300 -10.6010100H   -0.23769100  6.82258900 -11.0072060H   -0.25412900  9.12227900 -11.9786920H    1.24943600  10.8760470 -11.0447960H    2.76142100  10.3196440 -9.14699800H    2.76409700  8.03497000 -8.18182000H    1.17298000 -1.17618200 -3.08388600H   -0.19387000  1.04380300  0.29186700H    0.14201100 -0.61557200  0.89696600H   -0.85889900 -0.38608000 -0.57482700ɑ1a)2 Conformer-2C    0.00000000  0.00000000  0.00000000O   -1.20078500  0.10801400 -0.75710300C   -1.19327400 -0.59783100 -1.92843800C   -1.19098400  0.08422100 -3.12578400C   -1.20846100 -0.59806100 -4.36053600C   -1.21142300 -2.01636500 -4.36053600C   -1.22890000 -2.69864700 -3.12578400C   -1.22661000 -2.01659500 -1.92843800O   -1.21909900 -2.72244000 -0.75710300C   -2.41988400 -2.61442600  0.00000000H   -2.27486200 -3.22749500  0.89814500H   -3.27608000 -3.00673300 -0.57451800H   -2.61962200 -1.57113600  0.28974300H   -1.24660300 -3.78785900 -3.08544300C   -1.21170400 -2.72383800 -5.62184500C   -1.21062200 -2.01632900 -6.82561700C   -1.20926200 -0.59809700 -6.82561700C   -1.20818000  0.10941200 -5.62184500C   -1.20981400  1.52011300 -5.84272800H   -1.21156200  2.28306000 -5.06679400C   -1.21040100  1.86062200 -7.16712000S   -1.20954100  0.45388900 -8.20574200C   -1.21007000  3.20208200 -7.74207900H   -1.23050800  3.27204500 -8.84493600N   -1.17519400  4.23441600 -6.99181000C   -1.22904600  5.50924200 -7.58721900C   -2.11454800  5.82663800 -8.62655400C   -2.13257000  7.11328400 -9.16149300C   -1.27081000  8.09268700 -8.67160200C   -0.39892400  7.78303800 -7.62623100C   -0.38730200  6.50575100 -7.07643000H    0.27876000  6.25130300 -6.25081100H    0.27219900  8.54720600 -7.22978300H   -1.28809300  9.09922900 -9.09254000H   -2.83301200  7.35240200 -9.96391100H   -2.81339900  5.07116900 -8.99170400S   -1.21034300 -3.06831500 -8.20574200C   -1.20948300 -4.47504800 -7.16712000C   -1.21007000 -4.13453900 -5.84272800H   -1.20832200 -4.89748600 -5.06679400C   -1.20981400 -5.81650800 -7.74207900H   -1.18937600 -5.88647100 -8.84493600N   -1.24469000 -6.84884200 -6.99181000C   -1.19083800 -8.12366800 -7.58721900C   -0.30533600 -8.44106400 -8.62655400C   -0.28731400 -9.72771000 -9.16149300C   -1.14907400 -10.7071130 -8.67160200C   -2.02096000 -10.3974640 -7.62623100C   -2.03258200 -9.12017700 -7.07643000H   -2.69864400 -8.86572900 -6.25081100H   -2.69208300 -11.1616320 -7.22978300H   -1.13179100 -11.7136550 -9.09254000H    0.41312800 -9.96682800 -9.96391100H    0.39351500 -7.68559500 -8.99170400H   -1.17328100  1.17343300 -3.08544300H    0.19973800 -1.04329000  0.28974300H   -0.14502200  0.61306900  0.89814500H    0.85619600  0.39230700 -0.57451800ɑ1b)2 Conformer-1C    0.00000000  0.00000000  0.00000000C   -0.01299900  1.37660100  0.61311400C    0.78605300  2.40016200  0.08466000C    0.77102600  3.67862300  0.62528600C   -0.02578900  3.96793600  1.74091600N    0.00149600  5.28145600  2.24637300C   -0.01712000  5.46456700  3.50957600C   -0.03731100  6.80275700  4.07611400C   -0.04272400  7.13550300  5.40302100C   -0.06190100  8.54714200  5.63070500C   -0.07080500  9.25126400  6.89233000C   -0.08158900  8.57016500  8.12840000C   -0.08688700  9.25116900  9.32599300C   -0.06786500  10.6700990  9.32599300C   -0.07316300  11.3511030  8.12840000C   -0.08394700  10.6700040  6.89233000C   -0.09285100  11.3741260  5.63070500C   -0.08528600  10.6698570  4.42550700C   -0.06946600  9.25141100  4.42550700S   -0.05581400  8.20562700  3.03682300S   -0.09893800  11.7156410  3.03682300C   -0.11744100  13.1185110  4.07611400C   -0.11202800  12.7857650  5.40302100H   -0.12373300  13.5379450  6.19107600C   -0.13763200  14.4567010  3.50957600H   -0.14878800  15.2858550  4.24041200N   -0.15624800  14.6398120  2.24637300C   -0.12896300  15.9533320  1.74091600C   -0.92577800  16.2426450  0.62528600C   -0.94080500  17.5211060  0.08466000C   -0.14175300  18.5446670  0.61311400C    0.67378300  18.2385750  1.70447900C    0.68439600  16.9632480  2.26716500H    1.35557000  16.7422400  3.09985800H    1.32402900  19.0106370  2.12310200C   -0.15475200  19.9212680  0.00000000H    0.19420200  19.8937850 -1.04438700H    0.49421700  20.6145230  0.55316700H   -1.17206300  20.3427650 -0.00690200H   -1.58103200  17.7301540 -0.77615600H   -1.53885400  15.4451420  0.20297800H   -0.06644000  12.4404770  8.17120100O   -0.08158900  11.3770540  10.4974130C    1.11966200  11.2778820  11.2547130H    1.32598700  10.2363410  11.5462340H    0.97100500  11.8914850  12.1519930H    1.97374200  11.6741330  10.6795750O   -0.07316300  8.54421400  10.4974130C   -1.27441400  8.64338600  11.2547130H   -1.12575700  8.02978300  12.1519930H   -2.12849400  8.24713500  10.6795750H   -1.48073900  9.68492700  11.5462340H   -0.08831200  7.48079100  8.17120100H   -0.03101900  6.38332300  6.19107600H   -0.00596400  4.63541300  4.24041200C   -0.83914800  2.95802000  2.26716500C   -0.82853500  1.68269300  1.70447900H   -1.47878100  0.91063100  2.12310200H   -1.51032200  3.17902800  3.09985800H    1.38410200  4.47612600  0.20297800H    1.42628000  2.19111400 -0.77615600H   -0.64896900 -0.69325500  0.55316700H    1.01731100 -0.42149700 -0.00690200H   -0.34895400  0.02748300 -1.04438700ɑ1b)2 Conformer-2C    0.00000000  0.00000000  0.00000000C   -0.00965700  1.39540400  0.56908500C   -0.85308300  1.72295400  1.64002400C   -0.86256500  2.99908100  2.18622200C   -0.04568200  4.00542700  1.65361100N   -0.09884700  5.28095100  2.24738200C   -0.06211700  6.31343300  1.49730000C   -0.05998000  7.65458400  2.07313100C   -0.05970300  7.99554800  3.39730900C   -0.06240500  9.40633800  3.61798400C   -0.06256200  10.1139040  4.87910600C   -0.07809900  9.43167100  6.11396000C   -0.07682300  10.1136660  7.31127900C   -0.04782900  11.5324900  7.31127900C   -0.04655300  12.2144850  6.11396000C   -0.06209000  11.5322520  4.87910600C   -0.06224700  12.2398180  3.61798400C   -0.06211700  11.5321610  2.41436800C   -0.06253500  10.1139950  2.41436800S   -0.06224700  9.06166700  1.03445600S   -0.06240500  12.5844890  1.03445600C   -0.06467200  13.9915720  2.07313100C   -0.06494900  13.6506080  3.39730900H   -0.06780000  14.4133970  4.17343700C   -0.06253500  15.3327230  1.49730000H   -0.08291500  15.4016340  0.39437300N   -0.02580500  16.3652050  2.24738200C   -0.07897000  17.6407290  1.65361100C    0.73791300  18.6470750  2.18622200C    0.72843100  19.9232020  1.64002400C   -0.11499500  20.2507520  0.56908500C   -0.94959400  19.2505700  0.06632800C   -0.93634200  17.9605930  0.59471100H   -1.62263100  17.2062920  0.20394900H   -1.63399700  19.4829390 -0.75324100C   -0.12465200  21.6461560  0.00000000H   -0.40785100  22.3855650  0.76556800H   -0.83485800  21.7359200 -0.83382000H    0.87216700  21.9285370 -0.37351300H    1.38476800  20.6895970  2.06016300H    1.38659900  18.4001080  3.02797700H   -0.03150600  13.3037970  6.15388300O   -0.05830300  12.2386050  8.48268400C    1.14400000  12.1381900  9.23819100H    0.99780300  12.7537510  10.1344820H    1.99778500  12.5320760  8.66107900H    1.34868500  11.0968340  9.53139100O   -0.06634900  9.40755100  8.48268400C   -1.26865200  9.50796600  9.23819100H   -1.12245500  8.89240500  10.1344820H   -2.12243700  9.11408000  8.66107900H   -1.47333700  10.5493220  9.53139100H   -0.09314600  8.34235900  6.15388300H   -0.05685200  7.23275900  4.17343700H   -0.04173700  6.24452200  0.39437300C    0.81169000  3.68556300  0.59471100C    0.82494200  2.39558600  0.06632800H    1.50934500  2.16321700 -0.75324100H    1.49797900  4.43986400  0.20394900H   -1.51125100  3.24604800  3.02797700H   -1.50942000  0.95655900  2.06016300H   -0.99681900 -0.28238100 -0.37351300H    0.28319900 -0.73940900  0.76556800H    0.71020600 -0.08976400 -0.83382000ɑ1c)2 Conformer-1C    0.00000000  0.00000000  0.00000000O    0.70764400 -0.29030700  1.18351900C    0.64753200 -1.55710900  1.66555200C    1.38546900 -1.80184600  2.83107900C    1.38799500 -3.06108900  3.41010700C    0.65457900 -4.11442700  2.83791500N    0.61730300 -5.42067200  3.36048600C    0.63201200 -5.59918600  4.62472800C    0.63656800 -6.93666200  5.19300100C    0.63756700 -7.26985800  6.51981300C    0.63872900 -8.68180900  6.74750800C    0.63861000 -9.38614200  8.00889100C    0.63405400 -10.8050820  8.00889100C    0.61486700 -11.4859090  9.24514300C    0.61808500 -10.8048840  10.4426400C    0.65457900 -9.38634000  10.4426400C    0.65779700 -8.70531500  9.24514300H    0.67807000 -7.61610300  9.28780800O    0.64959800 -8.67930900  11.6143810C    1.85010500 -8.79243500  12.3705700H    2.04438700 -9.83626900  12.6623080H    1.70969100 -8.17680400  13.2678250H    2.70844600 -8.40664500  11.7945860O    0.62306600 -11.5119150  11.6143810C   -0.57744100 -11.3987890  12.3705700H   -0.43702700 -12.0144200  13.2678250H   -1.43578200 -11.7845790  11.7945860H   -0.77172300 -10.3549550  12.6623080H    0.59459400 -12.5751210  9.28780800C    0.63393500 -11.5094150  6.74750800C    0.63511400 -10.8047620  5.54256200C    0.63755000 -9.38646200  5.54256200S    0.63800200 -8.34016900  4.15380800S    0.63466200 -11.8510550  4.15380800C    0.63609600 -13.2545620  5.19300100C    0.63509700 -12.9213660  6.51981300H    0.63733800 -13.6734500  7.30807200C    0.64065200 -14.5920380  4.62472800H    0.64488500 -15.4203640  5.35629000N    0.65536100 -14.7705520  3.36048600C    0.61808500 -16.0767970  2.83791500C   -0.11533100 -17.1301350  3.41010700C   -0.11280500 -18.3893780  2.83107900C    0.62513200 -18.6341150  1.66555200C    1.34333800 -17.5888260  1.07632900C    1.31996400 -16.3220400  1.65640000H    1.86644100 -15.4965580  1.19818300H    1.91588400 -17.7420140  0.16230100O    0.56502000 -19.9009170  1.18351900C    1.27266400 -20.1912240  0.00000000H    1.09152700 -21.2517460 -0.21127600H    0.91114500 -19.5870350 -0.85022800H    2.35759400 -20.0281680  0.12262200H   -0.69106900 -19.2086400  3.26001000H   -0.72332100 -16.9526290  4.29962400H    0.63532600 -6.51777400  7.30807200H    0.62777900 -4.77086000  5.35629000C   -0.04730000 -3.86918400  1.65640000C   -0.07067400 -2.60239800  1.07632900H   -0.64322000 -2.44921000  0.16230100H   -0.59377700 -4.69466600  1.19818300H    1.99598500 -3.23859500  4.29962400H    1.96373300 -0.98258400  3.26001000H    0.36151900 -0.60418900 -0.85022800H   -1.08493000 -0.16305600  0.12262200H    0.18113700  1.06052200 -0.21127600ɑ1c)2 Conformer-2C    0.00000000  0.00000000  0.00000000O    0.72766000  1.06065800 -0.57535300C    0.63685400  2.28324300  0.00586200C   -0.13025500  2.57423200  1.13837100C   -0.13480700  3.86825000  1.65552000C    0.58734100  4.89661300  1.04730700N    0.52077900  6.17686700  1.62901400C    0.54742000  7.20913900  0.87766500C    0.53317100  8.54976200  1.45439600C    0.52912700  8.89098100  2.77852900C    0.50973300  10.3017730  2.99937500C    0.50131200  11.0095560  4.26023700C    0.49263300  10.3272930  5.49530400C    0.48644000  11.0093010  6.69249700C    0.50131200  12.4282290  6.69249700C    0.49511900  13.1102370  5.49530400C    0.48644000  12.4279740  4.26023700C    0.47801900  13.1357570  2.99937500C    0.48587300  12.4276530  1.79606900C    0.50187900  11.0098770  1.79606900S    0.51507400  9.95721500  0.41599000S    0.47267800  13.4803150  0.41599000C    0.45458100  14.8877680  1.45439600C    0.45862500  14.5465490  2.77852900H    0.44641900  15.3094840  3.55435400C    0.44033200  16.2283910  0.87766500H    0.41674700  16.2936190 -0.22526600N    0.46697300  17.2606630  1.62901400C    0.40041100  18.5409170  1.04730700C    1.12255900  19.5692800  1.65552000C    1.11800700  20.8632980  1.13837100C    0.35089800  21.1542870  0.00586200C   -0.40689600  20.1378430 -0.59015800C   -0.38156600  18.8498260 -0.07884300H   -1.00514600  18.0802380 -0.53846700H   -1.02267500  20.3894300 -1.45449300O    0.26009200  22.3768720 -0.57535300C    0.98775200  23.4375300  0.00000000H    0.66905800  23.6352150  1.03826800H    0.77619400  24.3209070 -0.61405400H    2.07407100  23.2414300 -0.01256200H    1.70806700  21.6333080  1.63402300H    1.70832200  19.3350690  2.54555600H    0.49883200  14.1996340  5.53526000O    0.48459900  13.1337780  7.86454700C    1.68828400  13.0435610  8.61883100H    1.90392100  12.0033640  8.90855500H    1.53658400  13.6547520  9.51726400H    2.53761900  13.4479380  8.04234400O    0.50315300  10.3037520  7.86454700C   -0.70053200  10.3939690  8.61883100H   -0.54883200  9.78277800  9.51726400H   -1.54986700  9.98959200  8.04234400H   -0.91616900  11.4341660  8.90855500H    0.48892000  9.23789600  5.53526000H    0.54133300  8.12804600  3.55435400H    0.57100500  7.14391100 -0.22526600C    1.36931800  4.58770400 -0.07884300C    1.39464800  3.29968700 -0.59015800H    2.01042700  3.04810000 -1.45449300H    1.99289800  5.35729200 -0.53846700H   -0.72057000  4.10246100  2.54555600H   -0.72031500  1.80422200  1.63402300H    0.31869400 -0.19768500  1.03826800H    0.21155800 -0.88337700 -0.61405400H   -1.08631900  0.19610000 -0.01256200ɑ1d)2 Conformer-1C    0.00000000  0.00000000  0.00000000C   -0.04561600 -1.37055100  0.61493500C   -0.92094300 -2.33302900  0.10651400C   -0.92611500 -3.61601600  0.63540200C   -0.07797900 -3.94644400  1.70075900N   -0.12386000 -5.26028200  2.19528800C   -0.11033900 -5.44975100  3.45822600C   -0.10926800 -6.78815500  4.02082500C   -0.10898200 -7.12020000  5.34845600C   -0.10789300 -8.53117400  5.57611200C   -0.10803400 -9.23492700  6.83842300C   -0.08808900 -8.55384200  8.07393500C   -0.09124700 -9.23473800  9.27192300C   -0.12978500 -10.6535600  9.27192300C   -0.13294300 -11.3344560  8.07393500C   -0.11299800 -10.6533710  6.83842300C   -0.11313900 -11.3571240  5.57611200C   -0.11176400 -10.6534760  4.37022400C   -0.10926800 -9.23482200  4.37022400S   -0.10913200 -8.19036400  2.98131000S   -0.11190000 -11.6979340  2.98131000C   -0.11176400 -13.1001430  4.02082500C   -0.11205000 -12.7680980  5.34845600H   -0.10949600 -13.5203710  6.13649000C   -0.11069300 -14.4385470  3.45822600H   -0.10831200 -15.2664980  4.19044300N   -0.09717200 -14.6280160  2.19528800C   -0.14305300 -15.9418540  1.70075900C    0.70508300 -16.2722820  0.63540200C    0.69991100 -17.5552690  0.10651400C   -0.17541600 -18.5177470  0.61493500C   -1.04182900 -18.1899860  1.65608000C   -1.02755100 -16.9089690  2.19785900H   -1.72634700 -16.6465190  2.99417700H   -1.73246600 -18.9383640  2.04544700C   -0.22103200 -19.8882980  0.00000000F   -1.06965100 -19.9407330 -1.04426600F   -0.63029000 -20.8215650  0.87772500F    0.98232900 -20.2721240 -0.46145500H    1.37665100 -17.8123330 -0.70878500H    1.37428100 -15.5058770  0.24336800H   -0.15428500 -12.4235880  8.11740900O   -0.12612600 -11.3615640  10.4414340C   -1.32291600 -11.2424320  11.2043140H   -1.51009000 -10.1979690  11.4977770H   -1.18048100 -11.8597220  12.0998730H   -2.18556500 -11.6235860  10.6320240O   -0.09490600 -8.52673400  10.4414340C    1.10188400 -8.64586600  11.2043140H    0.95944900 -8.02857600  12.0998730H    1.96453300 -8.26471200  10.6320240H    1.28905800 -9.69032900  11.4977770H   -0.06674700 -7.46471000  8.11740900H   -0.11153600 -6.36792700  6.13649000H   -0.11272000 -4.62180000  4.19044300C    0.80651900 -2.97932900  2.19785900C    0.82079700 -1.69831200  1.65608000H    1.51143400 -0.94993400  2.04544700H    1.50531500 -3.24177900  2.99417700H   -1.59531300 -4.38242100  0.24336800H   -1.59768300 -2.07596500 -0.70878500F    0.40925800  0.93326700  0.87772500F   -1.20336100  0.38382600 -0.46145500F    0.84861900  0.05243500 -1.04426600ɑ1d)2 Conformer-2C    0.00000000  0.00000000  0.00000000C   -0.04260100  1.39859500  0.54820600C    0.84304500  2.36954600  0.08794700C    0.81439800  3.65192900  0.62853200C   -0.10261100  3.97276500  1.63794400N   -0.16186100  5.24299200  2.23480300C   -0.13127700  6.27701200  1.48553400C   -0.13371300  7.61725800  2.05942500C   -0.13494900  7.95709600  3.38449800C   -0.13751400  9.36710300  3.60542800C   -0.13784300  10.0741660  4.86725400C   -0.13567900  11.4922320  4.86725400C   -0.11808400  12.1745200  6.10162700C   -0.11979600  11.4926740  7.29936800C   -0.15372600  10.0737240  7.29936800C   -0.15543800  9.39187800  6.10162700H   -0.17328400  8.30268600  6.14266500O   -0.14704500  9.36682400  8.46899100C   -1.34491900  9.47979600  9.23130500H   -1.53835800  10.5235650  9.52320800H   -1.19889600  8.86452100  10.1276290H   -2.20478100  9.09229600  8.65918400O   -0.12647700  12.1995740  8.46899100C    1.07139700  12.0866020  9.23130500H    0.92537400  12.7018770  10.1276290H    1.93125900  12.4741020  8.65918400H    1.26483600  11.0428330  9.52320800H   -0.10023800  13.2637120  6.14266500C   -0.13600800  12.1992950  3.60542800C   -0.13666300  11.4923710  2.40096900C   -0.13685900  10.0740270  2.40096900S   -0.13567900  9.02333900  1.02061400S   -0.13784300  12.5430590  1.02061400C   -0.13980900  13.9491400  2.05942500C   -0.13857300  13.6093020  3.38449800H   -0.14055000  14.3717740  4.16097800C   -0.14224500  15.2893860  1.48553400H   -0.16033500  15.3604950  0.38273000N   -0.11166100  16.3234060  2.23480300C   -0.17091100  17.5936330  1.63794400C   -1.08792000  17.9144690  0.62853200C   -1.11656700  19.1968520  0.08794700C   -0.23092100  20.1678030  0.54820600C    0.67882100  19.8578840  1.56327100C    0.69800700  18.5855810  2.11463100H    1.39383200  18.3307550  2.91454300H    1.37100300  20.6194390  1.92440500C   -0.27352200  21.5663980  0.00000000F    0.96187600  22.0652960 -0.18900500F   -0.90360200  22.4121370  0.83745300F   -0.91637800  21.6322470 -1.17820000H   -1.83428200  19.4414940 -0.69499800H   -1.80181100  17.1633330  0.28586500H   -0.13297200  7.19462400  4.16097800H   -0.11318700  6.20590300  0.38273000C   -0.97152900  2.98081700  2.11463100C   -0.95234300  1.70851400  1.56327100H   -1.64452500  0.94695900  1.92440500H   -1.66735400  3.23564300  2.91454300H    1.52828900  4.40306500  0.28586500H    1.56076000  2.12490400 -0.69499800F    0.64285600 -0.06584900 -1.17820000F    0.63008000 -0.84573900  0.83745300F   -1.23539800 -0.49889800 -0.18900500ɑ1bb)2 Conformer-1C    0.00000000  0.00000000  0.00000000O   -1.19899600  0.11569100 -0.75856800C   -1.19446400 -0.59205900 -1.92960400C   -1.19136600  0.08884300 -3.12735600C   -1.21068400 -0.59191300 -4.36340700C   -1.21528400 -2.01073700 -4.36340700C   -1.23460200 -2.69149300 -3.12735600C   -1.23150400 -2.01059100 -1.92960400O   -1.22697200 -2.71834100 -0.75856800C   -2.42596800 -2.60265000  0.00000000H   -2.28299400 -3.21461400  0.89932000H   -3.28521500 -2.99138600 -0.57255800H   -2.62019900 -1.55785800  0.28811200H   -1.25501300 -3.78070400 -3.08446900C   -1.21532600 -2.71492400 -5.62503300C   -1.21404000 -2.01064600 -6.83019700C   -1.21192800 -0.59200400 -6.83019700C   -1.21064200  0.11227400 -5.62503300C   -1.21181700  1.52408100 -5.85273000H   -1.21425500  2.27636700 -5.06468700C   -1.21277100  1.85677100 -7.17955700S   -1.21184100  0.45381600 -8.21882800C   -1.21582500  3.19533900 -7.74641600H   -1.21702100  4.02562400 -7.01679700N   -1.23150400  3.37885600 -9.00922500C   -1.18932100  4.69408700 -9.51137700C   -2.02275100  5.00178400 -10.5952250C   -2.03243400  6.28078100 -11.1428420C   -1.16806000  7.24824500 -10.6099750C   -0.30748700  6.95688300 -9.55300900C   -0.32728500  5.66925600 -9.00396800H    0.36306700  5.41259600 -8.19626100C    0.64477500  7.99265300 -9.01094100H    0.51896600  8.11831100 -7.92444400H    1.69063100  7.69522900 -9.18682600H    0.48821300  8.97121800 -9.48557000H   -1.15810900  8.25137900 -11.0456300C   -2.95099000  6.63120200 -12.2855420H   -2.39656400  7.10887100 -13.1077060H   -3.45360300  5.73992900 -12.6857320H   -3.72926500  7.33994200 -11.9604660H   -2.66924100  4.21615100 -10.9907010S   -1.21412700 -3.05646600 -8.21882800C   -1.21319700 -4.45942100 -7.17955700C   -1.21415100 -4.12673100 -5.85273000H   -1.21171300 -4.87901700 -5.06468700C   -1.21014300 -5.79798900 -7.74641600H   -1.20894700 -6.62827400 -7.01679700N   -1.19446400 -5.98150600 -9.00922500C   -1.23664700 -7.29673700 -9.51137700C   -2.09868300 -8.27190600 -9.00396800C   -2.11848100 -9.55953300 -9.55300900C   -1.25790800 -9.85089500 -10.6099750C   -0.39353400 -8.88343100 -11.1428420C   -0.40321700 -7.60443400 -10.5952250H    0.24327300 -6.81880100 -10.9907010C    0.52502200 -9.23385200 -12.2855420H    1.30329700 -9.94259200 -11.9604660H   -0.02940400 -9.71152100 -13.1077060H    1.02763500 -8.34257900 -12.6857320H   -1.26785900 -10.8540290 -11.0456300C   -3.07074300 -10.5953030 -9.01094100H   -4.11659900 -10.2978790 -9.18682600H   -2.94493400 -10.7209610 -7.92444400H   -2.91418100 -11.5738680 -9.48557000H   -2.78903500 -8.01524600 -8.19626100H   -1.17095500  1.17805400 -3.08446900H    0.19423100 -1.04479200  0.28811200H   -0.14297400  0.61196400  0.89932000H    0.85924700  0.38873600 -0.57255800ɑ1bb)2 Conformer-2C    0.00000000  0.00000000  0.00000000O   -1.20198000  0.10282300 -0.75586200C   -1.19181400 -0.60296400 -1.92747100C   -1.19165900  0.07907900 -3.12475000C   -1.20666800 -0.60317500 -4.35963400C   -1.20510800 -2.02155900 -4.35963400C   -1.22011700 -2.70381300 -3.12475000C   -1.21996200 -2.02177000 -1.92747100O   -1.20979600 -2.72755700 -0.75586200C   -2.41177600 -2.62473400  0.00000000H   -2.26502700 -3.23709900  0.89839200H   -3.26569600 -3.02084400 -0.57534800H   -2.61627400 -1.58224200  0.28944500H   -1.23432800 -3.79311300 -3.08453600C   -1.20320300 -2.72913900 -5.62074800C   -1.20437900 -2.02144900 -6.82434000C   -1.20739700 -0.60328500 -6.82434000C   -1.20857300  0.10440500 -5.62074800C   -1.21046600  1.51526000 -5.84165400H   -1.21313100  2.27839500 -5.06585900C   -1.21102600  1.85596300 -7.16578000S   -1.20970800  0.44904300 -8.20439400C   -1.21600200  3.19759600 -7.74139000H   -1.20177600  3.26821300 -8.84430000N   -1.24697300  4.22975600 -6.99126900C   -1.19811200  5.50462800 -7.58858000C   -2.05065100  6.49394600 -7.07949100C   -2.05440000  7.77550500 -7.62086600C   -1.16480300  8.06853000 -8.66497800C   -0.28572200  7.11039900 -9.16653300C   -0.31176500  5.82128600 -8.62085900H    0.39218900  5.06571000 -8.97910700C    0.69192600  7.44372400 -10.2648840H    0.54425400  8.46627300 -10.6390080H    0.58505600  6.75235500 -11.1149500H    1.72984300  7.36173000 -9.90543400H   -1.15024800  9.07837000 -9.08467900C   -2.99395900  8.83576100 -7.10566100H   -2.45324400  9.76565700 -6.87247100H   -3.51369000  8.50565300 -6.19564800H   -3.75840300  9.08220000 -7.85973500H   -2.71740100  6.23024300 -6.25631900S   -1.20206800 -3.07377700 -8.20439400C   -1.20075000 -4.48069700 -7.16578000C   -1.20131000 -4.13999400 -5.84165400H   -1.19864500 -4.90312900 -5.06585900C   -1.19577400 -5.82233000 -7.74139000H   -1.21000000 -5.89294700 -8.84430000N   -1.16480300 -6.85449000 -6.99126900C   -1.21366400 -8.12936200 -7.58858000C   -0.36112500 -9.11868000 -7.07949100C   -0.35737600 -10.4002390 -7.62086600C   -1.24697300 -10.6932640 -8.66497800C   -2.12605400 -9.73513300 -9.16653300C   -2.10001100 -8.44602000 -8.62085900H   -2.80396500 -7.69044400 -8.97910700C   -3.10370200 -10.0684580 -10.2648840H   -2.95603000 -11.0910070 -10.6390080H   -2.99683200 -9.37708900 -11.1149500H   -4.14161900 -9.98646400 -9.90543400H   -1.26152800 -11.7031040 -9.08467900C    0.58218300 -11.4604950 -7.10566100H    0.04146800 -12.3903910 -6.87247100H    1.10191400 -11.1303870 -6.19564800H    1.34662700 -11.7069340 -7.85973500H    0.30562500 -8.85497700 -6.25631900H   -1.17744800  1.16837900 -3.08453600H    0.20449800 -1.04249200  0.28944500H   -0.14674900  0.61236500  0.89839200H    0.85392000  0.39611000 -0.57534800ɑ1cc)2 Conformer-1C    0.00000000  0.00000000  0.00000000O   -1.20103600  0.11330200 -0.75543200C   -1.19686000 -0.59358100 -1.92718100C   -1.19292900  0.08752700 -3.12472600C   -1.21290600 -0.59342700 -4.36073800C   -1.21891200 -2.01217500 -4.36073800C   -1.23888900 -2.69312900 -3.12472600C   -1.23495800 -2.01202100 -1.92718100O   -1.23078200 -2.71890400 -0.75543200C   -2.43181800 -2.60560200  0.00000000H   -2.29197300 -3.22130200  0.89729400H   -3.28979100 -2.99134000 -0.57651700H   -2.62616500 -1.56178900  0.29173700H   -1.26029200 -3.78232200 -3.08206900C   -1.21932700 -2.71633500 -5.62241700C   -1.21730800 -2.01218200 -6.82772000C   -1.21451000 -0.59342000 -6.82772000C   -1.21249100  0.11073300 -5.62241700C   -1.21290600  1.52245900 -5.85005300H   -1.21432900  2.27499800 -5.06226500C   -1.21397400  1.85495600 -7.17692000S   -1.21492800  0.45227700 -8.21627100C   -1.21594800  3.19408900 -7.74243600H   -1.21619200  4.02399800 -7.01275100N   -1.23001700  3.37817900 -9.00493700C   -1.19059100  4.69565600 -9.50215400C   -2.03782200  5.00580700 -10.5673430C   -2.03172400  6.29553000 -11.0955800C   -1.16598400  7.27572100 -10.5899810C   -0.30518900  6.93920800 -9.53902800C   -0.31419200  5.65648500 -8.98865100H    0.39270100  5.41914300 -8.19373000O    0.58367900  7.80422300 -8.98730600C    0.65291800  9.11271900 -9.50341800H   -0.30376400  9.65086200 -9.38482600H    0.94043100  9.11744500 -10.5692440H    1.42681700  9.62825400 -8.92242900H   -1.15351800  8.27462300 -11.0167050O   -2.90100300  6.51665500 -12.1134700C   -2.93281500  7.79504300 -12.7027270H   -3.21698200  8.57492900 -11.9746700H   -3.69429800  7.74778500 -13.4901890H   -1.96243900  8.06106800 -13.1570970H   -2.70534700  4.25087000 -10.9797460S   -1.21689000 -3.05787900 -8.21627100C   -1.21784400 -4.46055800 -7.17692000C   -1.21891200 -4.12806100 -5.85005300H   -1.21748900 -4.88060000 -5.06226500C   -1.21587000 -5.79969100 -7.74243600H   -1.21562600 -6.62960000 -7.01275100N   -1.20180100 -5.98378100 -9.00493700C   -1.24122700 -7.30125800 -9.50215400C   -2.11762600 -8.26208700 -8.98865100C   -2.12662900 -9.54481000 -9.53902800C   -1.26583400 -9.88132300 -10.5899810C   -0.40009400 -8.90113200 -11.0955800C   -0.39399600 -7.61140900 -10.5673430H    0.27352900 -6.85647200 -10.9797460O    0.46918500 -9.12225700 -12.1134700C    0.50099700 -10.4006450 -12.7027270H    0.78516400 -11.1805310 -11.9746700H    1.26248000 -10.3533870 -13.4901890H   -0.46937900 -10.6666700 -13.1570970H   -1.27830000 -10.8802250 -11.0167050O   -3.01549700 -10.4098250 -8.98730600C   -3.08473600 -11.7183210 -9.50341800H   -3.85863500 -12.2338560 -8.92242900H   -2.12805400 -12.2564640 -9.38482600H   -3.37224900 -11.7230470 -10.5692440H   -2.82451900 -8.02474500 -8.19373000H   -1.17152600  1.17672000 -3.08206900H   -0.13984500  0.61570000  0.89729400H    0.85797300  0.38573800 -0.57651700H    0.19434700 -1.04381300  0.29173700ɑ1cc)2 Conformer-2C    0.00000000  0.00000000  0.00000000O   -1.20254100  0.10260700 -0.75462500C   -1.19296500 -0.60279500 -1.92662300C   -1.19175700  0.07942000 -3.12379600C   -1.20754600 -0.60294600 -4.35858300C   -1.20812800 -2.02133200 -4.35858300C   -1.22391700 -2.70369800 -3.12379600C   -1.22270900 -2.02148300 -1.92662300O   -1.21313300 -2.72688500 -0.75462500C   -2.41567400 -2.62427800  0.00000000H   -2.26959400 -3.23624100  0.89878300H   -3.26902200 -3.02085400 -0.57585200H   -2.62085100 -1.58177700  0.28892500H   -1.23940000 -3.79295700 -3.08370600C   -1.20754600 -2.72890700 -5.61977800C   -1.20760200 -2.02126900 -6.82345000C   -1.20807200 -0.60300900 -6.82345000C   -1.20812800  0.10462900 -5.61977800C   -1.20754600  1.51540000 -5.84068700H   -1.20935600  2.27873600 -5.06511500C   -1.20698700  1.85582900 -7.16493500S   -1.20834800  0.44909300 -8.20350200C   -1.20814500  3.19766700 -7.74004500H   -1.19751400  3.26966700 -8.84261200N   -1.23108800  4.22876100 -6.98849800C   -1.18386100  5.50290300 -7.58812700C   -2.04353200  6.48230100 -7.08687100C   -2.02968600  7.75887300 -7.64587700C   -1.14400500  8.07954300 -8.68451500C   -0.27137900  7.09245600 -9.15650500C   -0.28779700  5.80458300 -8.61807900H    0.42781300  5.07000500 -8.98704700O    0.63667400  7.30010100 -10.1435560C    0.71061000  8.57975100 -10.7269360H   -0.23829300  8.86253600 -11.2152170H    1.49957600  8.52268200 -11.4861520H    0.97987000  9.35236400 -9.98581500H   -1.12482400  9.08050400 -9.10606600O   -2.91192100  8.64182200 -7.11380500C   -2.94033600  9.95314000 -7.62598200H   -1.97484400  10.4694350 -7.48430700H   -3.71499400  10.4838360 -7.05992100H   -3.20453800  9.96871400 -8.69781400H   -2.72795100  6.25217100 -6.27188100S   -1.20732600 -3.07337100 -8.20350200C   -1.20868700 -4.48010700 -7.16493500C   -1.20812800 -4.13967800 -5.84068700H   -1.20631800 -4.90301400 -5.06511500C   -1.20752900 -5.82194500 -7.74004500H   -1.21816000 -5.89394500 -8.84261200N   -1.18458600 -6.85303900 -6.98849800C   -1.23181300 -8.12718100 -7.58812700C   -0.37214200 -9.10657900 -7.08687100C   -0.38598800 -10.3831510 -7.64587700C   -1.27166900 -10.7038210 -8.68451500C   -2.14429500 -9.71673400 -9.15650500C   -2.12787700 -8.42886100 -8.61807900H   -2.84348700 -7.69428300 -8.98704700O   -3.05234800 -9.92437900 -10.1435560C   -3.12628400 -11.2040290 -10.7269360H   -2.17738100 -11.4868140 -11.2152170H   -3.91525000 -11.1469600 -11.4861520H   -3.39554400 -11.9766420 -9.98581500H   -1.29085000 -11.7047820 -9.10606600O    0.49624700 -11.2661000 -7.11380500C    0.52466200 -12.5774180 -7.62598200H   -0.44083000 -13.0937130 -7.48430700H    1.29932000 -13.1081140 -7.05992100H    0.78886400 -12.5929920 -8.69781400H    0.31227700 -8.87644900 -6.27188100H   -1.17627400  1.16867900 -3.08370600H    0.20517700 -1.04250100  0.28892500H   -0.14608000  0.61196300  0.89878300H    0.85334800  0.39657600 -0.57585200ɑ1dd)2 Conformer-1C    0.00000000  0.00000000  0.00000000O   -1.19084300 -0.16257200 -0.76488100C   -1.02235500 -0.85050100 -1.93351600C   -1.17805500 -0.18761700 -3.13163700C   -1.03883200 -0.85485000 -4.36677800C   -0.71299400 -2.23515200 -4.36677800C   -0.57377100 -2.90238500 -3.13163700C   -0.72947100 -2.23950100 -1.93351600O   -0.56098300 -2.92743000 -0.76488100C   -1.75182600 -3.09000200  0.00000000H   -1.46823000 -3.65839200  0.89424600H   -2.50313300 -3.66044800 -0.57177300H   -2.17572200 -2.11787200  0.29526200H   -0.34099300 -3.96655600 -3.08805700C   -0.54870100 -2.91909500 -5.62951300C   -0.71124200 -2.23499200 -6.83594200C   -1.04058400 -0.85501000 -6.83594200C   -1.20312500 -0.17090700 -5.62951300C   -1.53386000  1.20019500 -5.85672000H   -1.71128200  1.93107400 -5.06853600C   -1.61277900  1.52277200 -7.18473800S   -1.28578400  0.15975200 -8.22477900C   -1.92667700  2.82384000 -7.74388300H   -2.11838000  3.62738200 -7.00953600N   -1.98826300  3.00839400 -9.00684100C   -2.24846800  4.29826700 -9.49511000C   -3.14773600  4.42290500 -10.5643780C   -3.43513100  5.67415500 -11.0881160C   -2.81394900  6.82151300 -10.5862960C   -1.90239000  6.69160400 -9.54863300C   -1.61605100  5.43998000 -8.99920300H   -0.87641800  5.35127300 -8.20198300C   -1.17805500  7.89494900 -9.00378200F   -1.29427800  7.97075500 -7.66530600F    0.13742900  7.83868300 -9.27352200F   -1.64304100  9.04417700 -9.51321300H   -3.03101700  7.79995700 -11.0119420C   -4.43815000  5.82744600 -12.2024400F   -3.95962100  6.60218800 -13.1905060F   -4.78306000  4.65025700 -12.7432440F   -5.56703900  6.41177600 -11.7636460H   -3.61510800  3.52414300 -10.9646930S   -0.46604200 -3.24975400 -8.22477900C   -0.13904700 -4.61277400 -7.18473800C   -0.21796600 -4.29019700 -5.85672000H   -0.04054400 -5.02107600 -5.06853600C    0.17485100 -5.91384200 -7.74388300H    0.36655400 -6.71738400 -7.00953600N    0.23643700 -6.09839600 -9.00684100C    0.49664200 -7.38826900 -9.49511000C   -0.13577500 -8.52998200 -8.99920300C    0.15056400 -9.78160600 -9.54863300C    1.06212300 -9.91151500 -10.5862960C    1.68330500 -8.76415700 -11.0881160C    1.39591000 -7.51290700 -10.5643780H    1.86328200 -6.61414500 -10.9646930C    2.68632400 -8.91744800 -12.2024400F    3.81521300 -9.50177800 -11.7636460F    2.20779500 -9.69219000 -13.1905060F    3.03123400 -7.74025900 -12.7432440H    1.27919100 -10.8899590 -11.0119420C   -0.57377100 -10.9849510 -9.00378200F   -1.88925500 -10.9286850 -9.27352200F   -0.45754800 -11.0607570 -7.66530600F   -0.10878500 -12.1341790 -9.51321300H   -0.87540800 -8.44127500 -8.20198300H   -1.41083300  0.87655400 -3.08805700H    0.42389600 -0.97213000  0.29526200H   -0.28359600  0.56839000  0.89424600H    0.75130700  0.57044600 -0.57177300ɑ1dd)2 Conformer-2C    0.00000000  0.00000000  0.00000000O   -1.19660800  0.11578800 -0.76457900C   -1.19136200 -0.59161000 -1.93322400C   -1.18875800  0.09016400 -3.13119300C   -1.20747500 -0.59219700 -4.36523700C   -1.21168900 -2.01002900 -4.36523700C   -1.23040600 -2.69239000 -3.13119300C   -1.22780200 -2.01061600 -1.93322400O   -1.22255600 -2.71801400 -0.76457900C   -2.41916400 -2.60222600  0.00000000H   -2.27334200 -3.21923000  0.89507000H   -3.28087300 -2.98646100 -0.57138600H   -2.60889000 -1.55831900  0.29360400H   -1.25036600 -3.78151300 -3.08976400C   -1.21233100 -2.71669600 -5.62750800C   -1.21072000 -2.01036600 -6.83261900C   -1.20844400 -0.59186000 -6.83261900C   -1.20683300  0.11447000 -5.62750800C   -1.20424900  1.52388200 -5.84816200H   -1.20462100  2.28578900 -5.07113700C   -1.20364600  1.86349700 -7.17378300S   -1.20747500  0.45807300 -8.21299000C   -1.20305300  3.20310000 -7.74558900H   -1.19093800  3.27555700 -8.84841800N   -1.22835800  4.23697000 -6.99467900C   -1.17186900  5.50692600 -7.58989500C   -2.02777300  6.50045300 -7.09223300C   -2.00668200  7.77436000 -7.63956100C   -1.11564200  8.09880900 -8.66638600C   -0.24937200  7.12220100 -9.13639100C   -0.27191300  5.82983200 -8.60778200H    0.43401300  5.08086000 -8.96994900C    0.76042900  7.44100300 -10.2082110F    0.55769000  8.64742600 -10.7560880F    0.72302800  6.53471200 -11.2014670F    2.01272000  7.42723700 -9.72004900H   -1.09141600  9.10526200 -9.08124300C   -2.95979400  8.83379700 -7.14936000F   -2.32929400  10.0007020 -6.93435800F   -3.56380700  8.48431000 -6.00414500F   -3.92614100  9.07531400 -8.05241900H   -2.70765700  6.24929700 -6.27910300S   -1.21168900 -3.06029900 -8.21299000C   -1.21551800 -4.46572300 -7.17378300C   -1.21491500 -4.12610800 -5.84816200H   -1.21454300 -4.88801500 -5.07113700C   -1.21611100 -5.80532600 -7.74558900H   -1.22822600 -5.87778300 -8.84841800N   -1.19080600 -6.83919600 -6.99467900C   -1.24729500 -8.10915200 -7.58989500C   -0.39139100 -9.10267900 -7.09223300C   -0.41248200 -10.3765860 -7.63956100C   -1.30352200 -10.7010350 -8.66638600C   -2.16979200 -9.72442700 -9.13639100C   -2.14725100 -8.43205800 -8.60778200H   -2.85317700 -7.68308600 -8.96994900C   -3.17959300 -10.0432290 -10.2082110F   -2.97685400 -11.2496520 -10.7560880F   -3.14219200 -9.13693800 -11.2014670F   -4.43188400 -10.0294630 -9.72004900H   -1.32774800 -11.7074880 -9.08124300C    0.54063000 -11.4360230 -7.14936000F   -0.08987000 -12.6029280 -6.93435800F    1.14464300 -11.0865360 -6.00414500F    1.50697700 -11.6775400 -8.05241900H    0.28849300 -8.85152300 -6.27910300H   -1.16879800  1.17928700 -3.08976400H    0.18972600 -1.04390700  0.29360400H   -0.14582200  0.61700400  0.89507000H    0.86170900  0.38423500 -0.57138600
